# Supplementary material for: Stöber method to amorphous metal-organic frameworks and coordination polymers
Source: Nat Commun. 2024 Jun 27;15:5463. doi: 10.1038/s41467-024-49772-2 (PMC11211336; doi:10.1038/s41467-024-49772-2)
Supplement: Supplementary file 1 — Supplementary Information [file 41467_2024_49772_MOESM1_ESM.pdf]

--- Supplementary Information ---

Stöber Method to Amorphous Metal-Organic Frameworks and Coordination Polymers

Wei Zhang[1,2]*, Yanchen Liu[1], Henrik S. Jeppesen[3] and Nicola Pinna[1]*

1. Department of Chemistry, IRIS Adlershof & The Center for the Science of Materials Berlin, Humboldt-Universität zu Berlin, Brook-Taylor-Str. 2, 12489 Berlin, Germany.
2. Department of Colloid Chemistry, Max Planck Institute of Colloids and Interfaces, 14476 Potsdam, Germany.
3. Deutsches Elektronen-Synchrotron (DESY), Notkestrasse 85, 22607 Hamburg, Germany.

Table of contents

1. Supplementary Methods **1**

1.1 **MOFs Section** **2**

1.2 **Core-Nanoparticles Section** **11**

1.3 **Characterization** **14**

1.4 **Electrochemical test** **15**

2. Supplementary Figures and Tables **16**

3. Supplementary References **103**

1. Supplementary Methods

All chemical reagents in this work were bought from commercial sources and used without further purification.

1.1 MOFs Section:

Synthesis of amorphous ZIF-zni colloids

$Zn(NO_3)_2 \cdot 6H_2O$ (15 mg) and imidazole (4 mg) were dissolved in 10 mL of ethanol to form transparent mother solution in a 40 ml vial left uncapped. Then, a TEA solution was prepared by adding 0.5 mL of TEA to 11.5 mL of ethanol in a separate 40 ml vial left uncapped. Subsequently, the mother solution and the TEA solution were carefully sealed at room temperature in a 500 mL beaker. Throughout the reaction, the mother solution was gently stirred. The TEA vapor generated from the TEA solution gradually diffused into the mother solution. The amorphous ZIF-zni spheres were collected after 6h and washed with absolute ethanol for three times.

Synthesis of core-shell structures (amorphous ZIF-zni as shell): The synthesis protocol of core-shell structures is similar to that of amorphous ZIF-zni spheres. Typically, $Zn(NO_3)_2 \cdot 6H_2O$ (15 mg) and imidazole (4 mg) were dissolved in 10 mL of ethanol to form transparent mother solution in a 40 mL vial left uncapped followed by the addition of core-nanoparticles (0.5 mg). Then, a TEA solution was prepared by adding 0.5 mL of TEA to 11.5 mL of ethanol in a separate 40 mL vial left uncapped. Subsequently, the mother solution containing core-nanoparticles (0.5 mg) and the TEA solution were carefully sealed at room temperature in a 500 mL beaker. Throughout the reaction, the mother solution was gently stirred. The amorphous ZIF-zni-based core-shell colloids were obtained after 4h and washed with absolute ethanol for three times. For preparation of ZIF-zni-based core-shell structures, the following method is also effective. In a typical synthesis, $Zn(NO_3)_2 \cdot 6H_2O$ (4.5 mg) and imidazole (15 mg) were dissolved in 10 mL of ethanol to form transparent mother solution followed by the addition of core-nanoparticles (0.5 mg). The resulting suspension was aged for 3h. The obtained ZIF-zni-based core-shell colloids were collected by centrifugation.

Synthesis of amorphous Zn(2-ethylimidazole)$_2$ colloids

$Zn(NO_3)_2 \cdot 6H_2O$ (5 mg) and 2-ethylimidazole (10 mg) were dissolved in 10 mL of ethanol to form transparent mother solution in a 40 mL vial. It is closed using a cap with three holes (1 mm diameter). Then, a TEA solution was prepared by adding 3 mL of TEA to 9 mL of ethanol in a separate 40 mL vial. A cap punctured by three holes was used to close TEA solution. Subsequently, the mother solution and the TEA solution were carefully sealed at room temperature in a 500 mL beaker. Throughout the reaction, the mother solution was gently stirred. The TEA vapor generated from the TEA solution gradually diffused into the mother solution. The amorphous Zn(2-ethylimidazole)$_2$ spheres were collected after 24h and washed with absolute ethanol for three times.

Synthesis of core-shell structures (amorphous Zn(2-ethylimidazole)$_2$ as shell): The synthesis protocol of core-shell structures is similar to that of amorphous Zn(2-ethylimidazole)$_2$ spheres. $Zn(NO_3)_2 \cdot 6H_2O$ (5 mg) and 2-ethylimidazole (10 mg) were dissolved in 10 mL of ethanol to form transparent mother solution in a 40 mL vial followed by the addition of core-nanoparticles (0.5 mg). It is closed using a cap with three holes (1 mm diameter). Then, a TEA solution was prepared by adding 3 mL of TEA to 9 mL of ethanol in a separate 40 mL vial. A cap punctured by three holes was used to close TEA solution. Subsequently, the mother solution and the TEA solution were

carefully sealed at room temperature in a 500 mL beaker. Throughout the reaction, the mother solution was gently stirred. The TEA vapor generated from the TEA solution gradually diffused into the mother solution. The amorphous Zn(2-ethylimidazole)$_2$-based core-shell colloids were collected after 24h and washed with absolute ethanol for three times.

Synthesis of amorphous Co(2-ethylimidazole)$_2$ colloids

$Co(NO_3)_2 \cdot 6H_2O$ (10 mg) and 2-ethylimidazole (20 mg) were dissolved in 10 mL of ethanol to form transparent mother solution in a 40 mL vial left uncapped. Then, a TEA solution was prepared by adding 3 mL of TEA to 9 mL of ethanol in a separate 40 mL vial left uncapped. Subsequently, the mother solution and the TEA solution were carefully sealed at room temperature in a 500 mL beaker. Throughout the reaction, the mother solution was gently stirred. The TEA vapor generated from the TEA solution gradually diffused into the mother solution. The amorphous Co(2-ethylimidazole)$_2$ spheres were collected after 3h and washed with absolute ethanol for three times.

Synthesis of core-shell structures (amorphous Co(2-ethylimidazole)$_2$ as shell): The synthesis protocol of core-shell structures is similar to that of amorphous Co(2-ethylimidazole)$_2$ spheres. $Co(NO_3)_2 \cdot 6H_2O$ (10 mg) and 2-ethylimidazole (20 mg) were dissolved in 10 mL of ethanol to form transparent mother solution in a 40 mL vial left uncapped followed by the addition of core-nanoparticles (0.5 mg). Then, a TEA solution was prepared by adding 3 mL of TEA to 9 mL of ethanol in a separate 40 mL vial left uncapped. Subsequently, the mother solution and the TEA solution were carefully sealed at room temperature in a 500 mL beaker. Throughout the reaction, the mother solution was gently stirred. The TEA vapor generated from the TEA solution gradually diffused into the mother solution. The amorphous Co(2-ethylimidazole)$_2$-based core-shell colloids were collected after 3h and washed with absolute ethanol for three times.

Synthesis of amorphous Co(purine)$_2$ colloids

$CoCl_2 \cdot 6H_2O$ (15 mg) and purine (15 mg) were dissolved in 10 mL of methanol to form transparent mother solution in a 40 mL vial left uncapped. Then, a TEA solution was prepared by adding 3 mL of TEA to 9 mL of ethanol in a separate 40 mL vial. A cap punctured by two holes (1 mm diameter) was used to close TEA solution. Subsequently, the mother solution and the TEA solution were carefully sealed at room temperature in a 500 mL beaker. Throughout the reaction, the mother solution was gently stirred. The TEA vapor generated from the TEA solution gradually diffused into the mother solution. The amorphous Co(purine)$_2$ spheres were collected after 24h and washed with absolute ethanol for three times.

Synthesis of core-shell structures (amorphous Co(purine)$_2$ as shell): The synthesis protocol of core-shell structures is similar to that of amorphous Co(purine)$_2$ spheres. $CoCl_2 \cdot 6H_2O$ (15 mg) and purine (15 mg) were dissolved in 10 mL of methanol to form transparent mother solution in a 40 mL vial left uncapped followed by the addition of core-nanoparticles (0.5 mg). Then, a TEA solution was prepared by adding 3 mL of TEA to 9 mL of ethanol in a separate 40 mL vial. A cap punctured by two holes (1 mm diameter) was used to close TEA solution. Subsequently, the mother solution and the TEA solution were carefully sealed at room temperature in a 500 mL beaker. Throughout the reaction, the mother solution was gently stirred. The TEA vapor generated from the TEA solution gradually diffused into the mother solution. The amorphous Co(purine)$_2$-based core-shell colloids were collected after 24h and washed with absolute ethanol for three times.

Synthesis of amorphous Zn(5-chlorobenzimidazole)$_2$ colloids

$ZnCl_2$ (2 mg) and 5-chlorobenzimidazole (10 mg) were dissolved in 10 mL of ethanol to form transparent mother solution in a 40 mL vial. It is closed using a cap with three holes (1 mm diameter). Then, a TEA solution was prepared by adding 3 mL of TEA to 9 mL of ethanol in a separate 40 mL vial. A cap punctured by three holes was used to close TEA solution. Subsequently, the mother solution and the TEA solution were carefully sealed at room temperature in a 500 mL beaker. Throughout the reaction, the mother solution was gently stirred. The TEA vapor generated from the TEA solution gradually diffused into the mother solution. The amorphous Zn(5-chlorobenzimidazole)$_2$ spheres were collected after 48h and washed with absolute ethanol for three times.

Synthesis of core-shell structures (amorphous Zn(5-chlorobenzimidazole)$_2$ as shell): The synthesis protocol of core-shell structures is similar to that of amorphous Zn(5-chlorobenzimidazole)$_2$ spheres. $ZnCl_2$ (2 mg) and 5-chlorobenzimidazole (10 mg) were dissolved in 10 mL of ethanol to form transparent mother solution in a 40 mL vial followed by the addition of core-nanoparticles (0.5 mg). It is closed using a cap with three holes (1 mm diameter). Then, a TEA solution was prepared by adding 3 mL of TEA to 9 mL of ethanol in a separate 40 mL vial. A cap punctured by three holes was used to close TEA solution. Subsequently, the mother solution and the TEA solution were carefully sealed at room temperature in a 500 mL beaker. Throughout the reaction, the mother solution was gently stirred. The TEA vapor generated from the TEA solution gradually diffused into the mother solution. The amorphous Zn(5-chlorobenzimidazole)$_2$-based core-shell colloids were collected after 48h and washed with absolute ethanol for three times.

Synthesis of amorphous Co(5,6-dimethylbenzimidazole)$_2$ colloids

$CoCl_2 \cdot 6H_2O$ (8 mg) and 5,6-dimethylbenzimidazole (9 mg) were dissolved in a mixture of 1.7 mL of ethanol and 8.3 ml of methanol to form transparent mother solution in a 40 mL vial. It is closed using a cap with one hole (1 mm diameter). Then, a TEA solution was prepared by adding 0.5 mL of TEA to 11.5 mL of ethanol in a separate 40 mL vial. A cap punctured by one hole was used to close TEA solution. Subsequently, the mother solution and the TEA solution were carefully sealed at room temperature in a 500 mL beaker. Throughout the reaction, the mother solution was gently stirred. The TEA vapor generated from the TEA solution gradually diffused into the mother solution. The amorphous Co(5,6-dimethylbenzimidazole)$_2$ spheres were collected after 24h and washed with absolute ethanol for three times.

Synthesis of core-shell structures (amorphous Co(5,6-dimethylbenzimidazole)$_2$ as shell): The synthesis protocol of core-shell structures is similar to that of amorphous Co(5,6-dimethylbenzimidazole)$_2$ spheres. $CoCl_2 \cdot 6H_2O$ (8 mg) and 5,6-dimethylbenzimidazole (9 mg) were dissolved in a mixture of 1.7 mL of ethanol and 8.3 ml of methanol to form transparent mother solution in a 40 mL vial followed by the addition of core-nanoparticles (0.5 mg). It is closed using a cap with one hole (1 mm diameter). Then, a TEA solution was prepared by adding 0.5 mL of TEA to 11.5 mL of ethanol in a separate 40 mL vial. A cap punctured by one hole was used to close TEA solution. Subsequently, the mother solution and the TEA solution were carefully sealed at room temperature in a 500 mL beaker. Throughout the reaction, the mother solution was gently stirred. The TEA vapor generated from the TEA solution gradually diffused into the mother solution. The amorphous Co(5,6-dimethylbenzimidazole)$_2$-based core-shell colloids were collected after 24h and washed with absolute ethanol for three times.

Synthesis of amorphous SALEM-2 (Zn(imidazole)(2-methylimidazole)) colloids

$Zn(NO_3)_2 \cdot 6H_2O$ (15 mg), imidazole (1 mg) and 2-methylimidazole (3 mg) were dissolved in 10 mL of ethanol to form transparent mother solution in a 40 mL vial left uncapped. Then, a TEA solution was prepared by adding 1 mL of TEA to 11 mL of ethanol in a separate vial left uncapped. Subsequently, the mother solution and the TEA solution were carefully sealed at room temperature in a 500 mL beaker. Throughout the reaction, the mother solution was gently stirred. The TEA vapor generated from the TEA solution gradually diffused into the mother solution. The amorphous SALEM-2 spheres were collected after 3h and washed with absolute ethanol for three times.

Synthesis of amorphous Ni-BTC colloids

$NiCl_2 \cdot 6H_2O$ (5 mg) and Benzene-1,3,5-tricarboxylic acid (H_3BTC, 2 mg) were dissolved in 10 mL of methanol to form transparent mother solution in a 40 mL vial left uncapped. Then, a TEA solution was prepared by adding 0.5 mL of TEA to 11.5 mL of ethanol in a separate 40 mL vial left uncapped. Subsequently, the mother solution and the TEA solution were carefully sealed at room temperature in a 500 mL beaker. Throughout the reaction, the mother solution was gently stirred. The TEA vapor generated from the TEA solution gradually diffused into the mother solution. The amorphous Ni-BTC spheres were collected after 4h and washed with absolute ethanol for three times.

Synthesis of core-shell structures (amorphous Ni-BTC as shell): The synthesis protocol of core-shell structures is similar to that of amorphous Ni-BTC spheres. $NiCl_2 \cdot 6H_2O$ (5 mg) and Benzene-1,3,5-tricarboxylic acid (H_3BTC, 2 mg) were dissolved in 10 mL of methanol to form transparent mother solution in a 40 mL vial left uncapped followed by the addition of core-nanoparticles (0.5 mg). Then, a TEA solution was prepared by adding 0.5 mL of TEA to 11.5 mL of ethanol in a separate 40 mL vial left uncapped. Subsequently, the mother solution and the TEA solution were carefully sealed at room temperature in a 500 mL beaker. Throughout the reaction, the mother solution was gently stirred. The TEA vapor generated from the TEA solution gradually diffused into the mother solution. The amorphous Ni-BTC-based core-shell colloids were collected after 4h and washed with absolute ethanol for three times.

Synthesis of amorphous Ni-BPDC colloids

$NiCl_2 \cdot 6H_2O$ (4 mg) and biphenyl-4,4'-dicarboxylate (H_2bpdc, 4 mg) were dissolved in a mixture of 5 mL of ethanol and 5 ml of DMF to form transparent mother solution in a 40 mL vial left uncapped. Then, a TEA solution was prepared by adding 1 mL of TEA to 11 mL of ethanol in a separate 40 mL vial left uncapped. Subsequently, the mother solution and the TEA solution were carefully sealed at room temperature in a 500 mL beaker. Throughout the reaction, the mother solution was gently stirred. The TEA vapor generated from the TEA solution gradually diffused into the mother solution. The amorphous Ni-BPDC spheres were collected after 3h and washed with absolute ethanol for three times.

Synthesis of core-shell structures (amorphous Ni-BPDC as shell): The synthesis protocol of core-shell structures is similar to that of amorphous Ni-BPDC spheres. $NiCl_2 \cdot 6H_2O$ (4 mg) and biphenyl-4,4'-dicarboxylate (H_2bpdc, 4 mg) were dissolved in a mixture of 5 mL of ethanol and 5 ml of DMF to form transparent mother solution in a 40 mL vial left uncapped followed by the addition of core-nanoparticles (0.5 mg). Then, a TEA solution was prepared by adding 1 mL of TEA to 11 mL of ethanol in a separate 40 mL vial left uncapped. Subsequently, the mother solution and the TEA solution were carefully sealed at room temperature in a 500 mL beaker. Throughout the

reaction, the mother solution was gently stirred. The TEA vapor generated from the TEA solution gradually diffused into the mother solution. The amorphous Ni-BPDC-based core-shell colloids were collected after 3h and washed with absolute ethanol for three times.

Synthesis of amorphous Ni-BDC colloids

$NiCl_2 \cdot 6H_2O$ (4 mg) and terephthalic acid (H_2bdc, 4 mg) were dissolved in a mixture of 7 mL of ethanol and 3 ml of DMF to form transparent mother solution in a 40 mL vial left uncapped. Then, a TEA solution was prepared by adding 1 mL of TEA to 11 mL of ethanol in a separate 40 mL vial left uncapped. Subsequently, the mother solution and the TEA solution were carefully sealed at room temperature in a 500 mL beaker. Throughout the reaction, the mother solution was gently stirred. The TEA vapor generated from the TEA solution gradually diffused into the mother solution. The amorphous Ni-BDC spheres were collected after 3h and washed with absolute ethanol for three times.

Synthesis of core-shell structures (amorphous Ni-BDC as shell): The synthesis protocol of core-shell structures is similar to that of amorphous Ni-BDC spheres. $NiCl_2 \cdot 6H_2O$ (4 mg) and terephthalic acid (H_2bdc, 4 mg) were dissolved in a mixture of 7 mL of ethanol and 3 ml of DMF to form transparent mother solution in a 40 mL vial left uncapped followed by the addition of core-nanoparticles (0.5 mg). Then, a TEA solution was prepared by adding 1 mL of TEA to 11 mL of ethanol in a separate 40 mL vial left uncapped. Subsequently, the mother solution and the TEA solution were carefully sealed at room temperature in a 500 mL beaker. Throughout the reaction, the mother solution was gently stirred. The TEA vapor generated from the TEA solution gradually diffused into the mother solution. The amorphous Ni-BDC-based core-shell colloids were collected after 3h and washed with absolute ethanol for three times.

Synthesis of amorphous Nd-BDC colloids

$Nd(NO_3)_3 \cdot 6H_2O$ (5 mg) and terephthalic acid (H_2bdc, 2 mg) were dissolved in 10 mL of methanol to form transparent mother solution in a 40 mL vial left uncapped. Then, a TEA solution was prepared by adding 1 mL of TEA to 11 mL of ethanol in a separate 40 mL vial left uncapped. Subsequently, the mother solution and the TEA solution were carefully sealed at room temperature in a 500 mL beaker. Throughout the reaction, the mother solution was gently stirred. The TEA vapor generated from the TEA solution gradually diffused into the mother solution. The amorphous Nd-BDC spheres were collected after 1h and washed with absolute ethanol for three times.

Synthesis of amorphous Cr-NH_2-BDC colloids

$Cr(NO_3)_3 \cdot 9H_2O$ (10 mg) and 2-aminoterephthalic acid (NH_2-H_2BDC, 20 mg) were dissolved in a mixture of 5 mL of ethanol and 5 ml of H_2O to form transparent mother solution in a 40 mL vial. It is closed using a cap with three holes (1 mm diameter). Then, a TEA solution was prepared by adding 3 mL of TEA to 9 mL of ethanol in a separate 40 mL vial. A cap punctured by three holes was used to close TEA solution. Subsequently, the mother solution and the TEA solution were carefully sealed at room temperature in a 500 mL beaker. Throughout the reaction, the mother solution was gently stirred. The TEA vapor generated from the TEA solution gradually diffused into the mother solution. The amorphous Cr-NH_2-BDC spheres were collected after 20h and washed with absolute ethanol for three times.

Synthesis of core-shell structures (amorphous Cr-NH_2-BDC as shell): The synthesis protocol of core-shell structures is similar to that of amorphous Cr-NH2-BDC spheres. $Cr(NO_3)_3·9H_2O$ (10 mg) and 2-aminoterephthalic acid (NH_2-H_2BDC, 20 mg) were dissolved in a mixture of 5 mL of ethanol and 5 ml of H_2O to form transparent mother solution in a 40 mL vial followed by the addition of core-nanoparticles (0.5 mg). It is closed using a cap with three holes (1 mm diameter). Then, a TEA solution was prepared by adding 3 mL of TEA to 9 mL of ethanol in a separate 40 mL vial. A cap punctured by three holes was used to close TEA solution. Subsequently, the mother solution and the TEA solution were carefully sealed at room temperature in a 500 mL beaker. Throughout the reaction, the mother solution was gently stirred. The TEA vapor generated from the TEA solution gradually diffused into the mother solution. The amorphous Cr-NH_2-BDC-based core-shell colloids were collected after 20h and washed with absolute ethanol for three times.

Synthesis of amorphous Al-NH_2-BDC colloids

$Al(NO_3)_3·9H_2O$ (5 mg) and 2-aminoterephthalic acid (NH_2-H_2BDC, 5 mg) were dissolved in a mixture of 5 mL of ethanol and 5 ml of H_2O to form transparent mother solution in a 40 mL vial. It is closed using a cap with three holes (1 mm diameter). Then, a TEA solution was prepared by adding 3 mL of TEA to 9 mL of ethanol in a separate 40 mL vial. A cap punctured by three holes was used to close TEA solution. Subsequently, the mother solution and the TEA solution were carefully sealed at room temperature in a 500 mL beaker. Throughout the reaction, the mother solution was gently stirred. The TEA vapor generated from the TEA solution gradually diffused into the mother solution. The amorphous Al-NH_2-BDC spheres were collected after 20h and washed with absolute ethanol for three times.

Synthesis of amorphous In-IPA colloids

$InCl_3$ (6mg) and 1,3-isophthalic acid (H_2ipa, 2.6 mg) were dissolved in a mixture of 9 mL of ethanol and 1 ml of DMF to form transparent mother solution in a 40 mL vial left uncapped. Then, a TEA solution was prepared by adding 1 mL of TEA to 11 mL of ethanol in a separate 40 mL vial left uncapped. Subsequently, the mother solution and the TEA solution were carefully sealed at room temperature in a 500 mL beaker. Throughout the reaction, the mother solution was gently stirred. The TEA vapor generated from the TEA solution gradually diffused into the mother solution. The amorphous In-IPA spheres were collected after 3h and washed with absolute ethanol for three times.

Synthesis of core-shell structures (amorphous In-IPA as shell): The synthesis protocol of core-shell structures is similar to that of amorphous In-IPA spheres. $InCl_3$ (6mg) and 1,3-isophthalic acid (H_2ipa, 2.6 mg) were dissolved in a mixture of 9 mL of ethanol and 1 ml of DMF to form transparent mother solution in a 40 mL vial left uncapped followed by the addition of core-nanoparticles (0.5 mg). Then, a TEA solution was prepared by adding 1 mL of TEA to 11 mL of ethanol in a separate 40 mL vial left uncapped. Subsequently, the mother solution and the TEA solution were carefully sealed at room temperature in a 500 mL beaker. Throughout the reaction, the mother solution was gently stirred. The TEA vapor generated from the TEA solution gradually diffused into the mother solution. The amorphous In-IPA-based core-shell colloids were collected after 3h and washed with absolute ethanol for three times.

Synthesis of amorphous Al-BTC colloids

$Al(NO_3)_3 \cdot 9H_2O$ (5 mg) and Benzene-1,3,5-tricarboxylic acid (H_3BTC, 5 mg) were dissolved in a mixture of 2 mL of ethanol and 8 ml of H_2O to form transparent mother solution in a 40 mL vial. It is closed using a cap with three holes (1 mm diameter). Then, a TEA solution was prepared by adding 2 mL of TEA to 10 mL of ethanol in a separate 40 mL vial. A cap punctured by three holes was used to close TEA solution. Subsequently, the mother solution and the TEA solution were carefully sealed at room temperature in a 500 mL beaker. Throughout the reaction, the mother solution was gently stirred. The TEA vapor generated from the TEA solution gradually diffused into the mother solution. The amorphous Al-BTC spheres were collected after 24h and washed with absolute ethanol for three times.

Synthesis of core-shell structures (amorphous Al-BTC as shell): The synthesis protocol of core-shell structures is similar to that of amorphous Al-BTC spheres. $Al(NO_3)_3 \cdot 9H_2O$ (5 mg) and Benzene-1,3,5-tricarboxylic acid (H_3BTC, 5 mg) were dissolved in a mixture of 2 mL of ethanol and 8 ml of H_2O to form transparent mother solution in a 40 mL vial followed by the addition of core-nanoparticles (0.5 mg). It is closed using a cap with three holes (1 mm diameter). Then, a TEA solution was prepared by adding 2 mL of TEA to 10 mL of ethanol in a separate 40 mL vial. A cap punctured by three holes was used to close TEA solution. Subsequently, the mother solution and the TEA solution were carefully sealed at room temperature in a 500 mL beaker. Throughout the reaction, the mother solution was gently stirred. The TEA vapor generated from the TEA solution gradually diffused into the mother solution. The amorphous Al-BTC-based core-shell colloids were collected after 24h and washed with absolute ethanol for three times.

Synthesis of amorphous Mn-DSBDC colloids

$MnCl_2 \cdot 6H_2O$ (6 mg) and 2,5-disulfhydrylbenzene-1,4-dicarboxylic acid (H_4DSBDC, 2.6 mg) were dissolved in a mixture of 8 mL of methanol and 2 ml of DMF to form transparent mother solution in a 40 mL vial left uncapped. Then, a TEA solution was prepared by adding 1 mL of TEA to 11 mL of ethanol in a separate 40 mL vial left uncapped. Subsequently, the mother solution and the TEA solution were carefully sealed at room temperature in a 500 mL beaker. Throughout the reaction, the mother solution was gently stirred. The TEA vapor generated from the TEA solution gradually diffused into the mother solution. The amorphous Mn-DSBDC spheres were collected after 1h and washed with absolute ethanol for three times.

Synthesis of core-shell structures (amorphous Mn-DSBDC as shell): The synthesis protocol of core-shell structures is similar to that of amorphous Mn-DSBDC spheres. $MnCl_2 \cdot 6H_2O$ (6 mg) and 2,5-disulfhydrylbenzene-1,4-dicarboxylic acid (H_4DSBDC, 2.6 mg) were dissolved in a mixture of 8 mL of methanol and 2 ml of DMF to form transparent mother solution in a 40 mL vial left uncapped followed by the addition of core-nanoparticles (0.5 mg). Then, a TEA solution was prepared by adding 1 mL of TEA to 11 mL of ethanol in a separate 40 mL vial left uncapped. Subsequently, the mother solution and the TEA solution were carefully sealed at room temperature in a 500 mL beaker. Throughout the reaction, the mother solution was gently stirred. The TEA vapor generated from the TEA solution gradually diffused into the mother solution. The amorphous Mn-DSBDC-based core-shell colloids were collected after 1h and washed with absolute ethanol for three times.

Synthesis of amorphous Nd-PYDC colloids

$Nd(NO_3)_3 \cdot 6H_2O$ (5 mg) and 2,5-pyridinedicarboxylic acid (2,5-H_2pydc, 5 mg) were dissolved in 10 mL of methanol to form transparent mother solution in a 40 mL vial left uncapped. Then, a TEA solution was prepared by adding 2 mL of TEA to 10 mL of ethanol in a separate 40 mL vial left uncapped. Subsequently, the mother solution and TEA solution were carefully sealed at room temperature in a 500 mL beaker. Throughout the reaction, the mother solution was gently stirred. The TEA vapor generated from the TEA solution gradually diffused into the mother solution. The amorphous Nd-PYDC spheres were collected after 2h and washed with absolute ethanol for three times.

Synthesis of core-shell structures (amorphous Nd-PYDC as shell): The synthesis protocol of core-shell structures is similar to that of amorphous Nd-PYDC spheres. $Nd(NO_3)_3 \cdot 6H_2O$ (5 mg) and 2,5-pyridinedicarboxylic acid (2,5-H_2pydc, 5 mg) were dissolved in 10 mL of methanol to form transparent mother solution in a 40 mL vial left uncapped followed by the addition of core-nanoparticles (0.5 mg). Then, a TEA solution was prepared by adding 2 mL of TEA to 10 mL of ethanol in a separate 40 mL vial left uncapped. Subsequently, the mother solution and TEA solution were carefully sealed at room temperature in a 500 mL beaker. Throughout the reaction, the mother solution was gently stirred. The TEA vapor generated from the TEA solution gradually diffused into the mother solution. The amorphous Nd-PYDC-based core-shell colloids were collected after 2h and washed with absolute ethanol for three times.

Synthesis of amorphous Eu-PYDC colloids

$EuCl_3$ (10 mg) and 2,5-pyridinedicarboxylic acid (2,5-H_2pydc, 10 mg) were dissolved in a mixture of 5 mL of methanol and 5 ml of H_2O to form transparent mother solution in a 40 mL vial left uncapped. Then, a TEA solution was prepared by adding 2 mL of TEA to 10 mL of ethanol in a separate 40 mL vial left uncapped. Subsequently, the mother solution and the TEA solution were carefully sealed at room temperature in a 500 mL beaker. Throughout the reaction, the mother solution was gently stirred. The TEA vapor generated from the TEA solution gradually diffused into the mother solution. The amorphous Eu-PYDC spheres were collected after 4h and washed with absolute ethanol for three times.

Synthesis of core-shell structures (amorphous Eu-PYDC as shell): The synthesis protocol of core-shell structures is similar to that of amorphous Eu-PYDC spheres. $EuCl_3$ (10 mg) and 2,5-pyridinedicarboxylic acid (2,5-H_2pydc, 10 mg) were dissolved in a mixture of 5 mL of methanol and 5 ml of H_2O to form transparent mother solution in a 40 mL vial left uncapped followed by the addition of core-nanoparticles (0.5 mg). Then, a TEA solution was prepared by adding 2 mL of TEA to 10 mL of ethanol in a separate 40 mL vial left uncapped. Subsequently, the mother solution and the TEA solution were carefully sealed at room temperature in a 500 mL beaker. Throughout the reaction, the mother solution was gently stirred. The TEA vapor generated from the TEA solution gradually diffused into the mother solution. The amorphous Eu-PYDC-based core-shell colloids were collected after 4h and washed with absolute ethanol for three times.

Synthesis of amorphous Er-PYDC colloids

$Er(NO_3)_3$ (5 mg) and 2,5-pyridinedicarboxylic acid (2,5-H_2pydc, 5 mg) were dissolved in a mixture of 5 mL of methanol and 5 ml of H_2O to form transparent mother solution in a 40 mL vial left uncapped. Then, a TEA solution was prepared by adding 2 mL of TEA to 10 mL of ethanol in a

separate 40 mL vial left uncapped. Subsequently, the mother solution and the TEA solution were carefully sealed at room temperature in a 500 mL beaker. Throughout the reaction, the mother solution was gently stirred. The TEA vapor generated from the TEA solution gradually diffused into the mother solution. The amorphous Er-PYDC spheres were collected after 20h and washed with absolute ethanol for three times.

Synthesis of amorphous Gd-FDA colloids

$Gd(NO_3)_3 \cdot 6H_2O$ (10 mg) and furan-2,5-dicarboxylic acidic acid (H_2FDA, 5 mg) were dissolved in a mixture of 7 mL of methanol and 3 ml of ethanol to form transparent mother solution in a 40 mL vial left uncapped. Then, a TEA solution was prepared by adding 1 mL of TEA to 11 mL of ethanol in a separate 40 mL vial left uncapped. Subsequently, the mother solution and the TEA solution were carefully sealed at room temperature in a 500 mL beaker. Throughout the reaction, the mother solution was gently stirred. The TEA vapor generated from the TEA solution gradually diffused into the mother solution. The amorphous Gd-FDA spheres were collected after 3h and washed with absolute ethanol for three times.

Synthesis of amorphous In-BTC colloids

$InCl_3$ (12 mg) and benzene-1,3,5-tricarboxylic acid (H_3BTC, 5.3 mg) were dissolved in a mixture of 7 mL of ethanol and 3 ml of DMF to form transparent mother solution in a 40 mL vial left uncapped. Then, a TEA solution was prepared by adding 2 mL of TEA to 10 mL of ethanol in a separate 40 mL vial left uncapped. Subsequently, the mother solution and the TEA solution were carefully sealed at room temperature in a 500 mL beaker. Throughout the reaction, the mother solution was gently stirred. The TEA vapor generated from the TEA solution gradually diffused into the mother solution. The amorphous In-BTC spheres were collected after 3h and washed with absolute ethanol for three times.

1.2 Core-Nanoparticles Section:

All core-nanoparticles in this work were synthesized based on previous reports or bought from commercial sources and used without further purification.

Preparation of Ag-NPs: Silver nanoparticles were synthesized according to a previous report with slight modifications.[1] Polyvinylpyrrolidone (K30, 660 mg), $AgNO_3$ (288 mg) and NaCl (10mg) were dissolved in 7 ml ethylene glycol to form a clear solution. Then, 2 ml of glycerol was added and then the resulting solution was transferred into a 20 mL Teflon-lined autoclave. The Teflon container was sealed and heated it in an oven at 160 °C for 2 h. The final products were obtained through centrifugation of the reaction solution.

Preparation of Au-NPs: Gold nanoparticles were synthesized according to a previous report with slight modifications.[2] Briefly, 20 μl of 0.2 M $HAuCl_4$ and 32μl of 0.2 M ascorbic acid were added into 10 ml of 0.06 M CTAB aqueous solution in a vial at room temperature. After 1 min, 20 μl of 0.2 M $AgNO_3$ was added into the above solution and aged for 5h. The obtained products were collected by centrifugation.

Preparation of Pd-NCs: Platinum nanoparticles were synthesized according to a previous report with slight modifications.[3] Briefly, PVP (240 mg), ascorbic acid (120 mg), and KBr (800 mg) were dissolved in 16 mL of deionized water, resulting in a transparent solution. This mixed solution was then sealed in a glass vial and heated to 80 °C under magnetic stirring for 5 minutes. Subsequently, 6.0 mL of an aqueous solution containing 120 mg of $Pd(OAc)_2$ was introduced, and the mixture was stirred for 3 h. The obtained products were collected by centrifugation.
The synthesis of Pt-NCs resembles that of Pd NCs, differing only in the substitution of 120 mg of K_2PtCl_4 for 120 mg of $Pd(OAc)_2$.

Preparation of ZIF-67 nanocube:[4] $Co(NO_3)_2·6H_2O$ (59 mg) and CTAB (1 mg) were first dissolved in 2 ml of H_2O. Then, 908 mg of 2-Methylimidazole was dissolved in 14 ml of H_2O to form another solution. The two solutions were mixed and left to stir at room temperature for 20 minutes. The obtained products were collected by centrifugation.

Preparation of Prussian blue nanocube:[5] 132 mg of $K_3[Fe(CN)_6]·3\ H_2O$ and 3 g of PVP were initially dissolved in 40 ml of a 0.1 M HCl solution. Then, the mixed solution was sealed and heated at 80 °C for 24 h. The obtained products were collected by centrifugation.

Preparation of $Ni_3[Fe(CN)6]_2$ nanocube:[6] 143 mg of $NiCl_2·6H_2O$ and 300 mg of trisodium citrate dihydrate were dissolved in 20 ml of H_2O to form solution A. 132 mg of $K_3[Fe(CN)_6]·3\ H_2O$ was dissolved in 20 ml of H_2O to form solution B. Then, solution A was added into B and aged for 24h at room temperature. The obtained products were collected by centrifugation.

Preparation of Fe_2O_3 ellipsoids: The Fe_2O_3 nanoparticles were synthesized according to a previous report with slight modifications.[7] An aqueous solution containing 32 mg of $FeCl_3$ and 0.7 mg of KH_2PO_4 was sealed in a 20 mL Teflon-lined autoclave and heated at 105°C for 48h. The obtained products were collected by centrifugation.

Preparation of spindle-shaped β-FeOOH: The spindle-shaped β-FeOOH nanoparticles were synthesized according to a previous report with slight modifications.[8] 628 mg of $FeCl_3$ and 1g of PVP were dissolved in 40 ml of H_2O in a vial. Subsequently, the vial was sealed with a cap and stirred at 85°C for 10 h. The obtained products were obtained by centrifugation.

Preparation of α-Mn_2O_3 octahedra: The α-Mn_2O_3 octahedra were synthesized according to previous report.[9] 494 mg of PVP and 2.02 g of $Mn(NO_3)_2·4H_2O$ were dissolved in a mixture of 0.9 ml of H_2O and 10.5 ml of DMF to form a clear solution. This solution was transferred into a 20 mL Teflon-lined autoclave and maintained at 180 °C for 6 h. The obtained products were obtained by centrifugation.

Preparation of SnO_2 NPs: The SnO_2 NPs were synthesized according to a previous report with slight modifications.[10] 350 mg of $SnCl_4·5H_2O$, 315 mg of PVP and 0.4 ml of concentrated HCl acid were added into a mixture of 3 ml of H_2O and 3 ml of ethanol. The solution was transferred into a 20 mL Teflon-lined autoclave and maintained at 180 °C for 12 h. The obtained products were obtained by centrifugation.

Preparation of $H_2TiO_{2n+1}·xH_2O$ and TiO_2 nanowires: The synthesis is based on a previous report with slight modifications.[11] For $H_2TiO_{2n+1}·xH_2O$, 1 g of commercial TiO_2 was added into 10 ml of 12 M NaOH aqueous solution. The resulting suspension was sealed in a 20 ml Teflon-lined autoclave and maintained at 180 °C for 24 h. The product was initially isolated by centrifugation and subsequently immersed in 20 mL of 0.5 M HCl for 5h. Finally, the $H_2TiO_{2n+1}·xH_2O$ was collected by centrifugation.
The obtained $H_2TiO_{2n+1}·xH_2O$ (20 mg) were placed in a furnace and kept at 400 °C for 4 h for the preparation of TiO_2 nanowires.

Preparation of Cu_2O nanocubes: The Cu_2O nanocubes was prepared according to a previous work with slight modifications.[12] 50 mg of $Cu(OAc)_2$ and 1 mg of PVP were dissolved in 100 ml of H_2O. Then, 20 ml of 0.25 M NaOH solution was added into the copper acetate solution. After that, 15 ml of 50 mM ascorbic acid solution was dropwise added under vigorous stirring at room temperature. The final product was obtained after 30 min and isolated by centrifugation.

Preparation of $MnFe_2O_4$ spheres:[13] 150 mg of $MnCl_2·4H_2O$ and 240 mg of $FeCl_3$ were dissolved in 12 ml of ethylene glycol, subsequently followed by the addition of 1.08 g of NaAc and 300 mg PEG to form a clear solution under vigorous stirring. The solution was sealed in a 20 ml Teflon-lined autoclave and heated at 200 °C for 8h. The final product was isolated by centrifugation.

Preparation of BiOCl sheets: The BiOCl sheets were synthesized based on a previous report.[14] 485 mg of $Bi(NO_3)_3·5H_2O$ and 74 mg of KCl were dissolved in 21 ml of H_2O to form clear solution. Then, the resulting mixture solution was transferred into a 40 mL Teflon-lined stainless autoclave and maintained at 160 °C for 24 h. The final product was collected by centrifugation.

Preparation of $Ca_5(PO_4)_3OH$ nanorods: Hydroxyapatite (HAP) nanorods were synthesized according to a previous report with sight modifications.[15] 0.25 g of PEG and 2 ml of oleic acid were dissolved in 7.5 ml of ethanol. Subsequently, the $Ca(NO_3)_2·4H_2O$ solution (250 mg in 3.75 ml of H_2O), NaF solution (25 mg in 2.5 ml of H_2O) and $Na_3PO_4·12H_2O$ solution (250 mg in 3.75 ml of H_2O) were added into the above solution under vigorous stirring. The resulting mixture was placed in a 20 ml Teflon-line stainless autoclave and heated at 100 °C for 10 h. The final product was collected by centrifugation.

Preparation of $In(OH)_3$ NPs: Indium hydroxide nanoparticles were obtained based on a previous report with sight modifications.[16] 37 mg of $InCl_3$ and 353 mg of $C_6H_5Na_3O_7·2H_2O$ (sodium citrate) were dissolved in 12 ml of deionized water, followed by the addition of 60 mg urea under vigorous stirring. The mixture solution was then transferred into a 20 ml Teflon-lined stainless autoclave and maintained at 140 °C for 24h. The final product was collected by centrifugation.

Preparation of doped-$NaYF_4$ nanorods: The doped-$NaYF_4$ nanorods were synthesized based on a previous work.[17] 300 mg of NaOH were dissolved in a mixture of 1.5 ml of H_2O, 5 ml ethanol and 5 ml oleic acid. Then, 1ml of 2M NH_4F solution and 2 ml of salt solution containing 15.6 mg of YCl_3, 8.9 mg of Gd $(NO_3)_3·6H_2O$, 20 mg of $YbCl_3·6H_2O$ and 22 mg of $ErCl_3$ were added into the

above solution under vigorous stirring. The mixture solution was then transferred into a 20 ml Teflon-lined stainless autoclave and maintained at 200 °C for 2h. The final product was collected by centrifugation.

Preparation of PbS:[18] 2.57 ml of 0.1M CTAB, 2.04 ml of 0.5 M $Pb(Ac)_2$ and 4.1 ml of 1M HAc solution were mixed and followed by the addition of 10 ml of 0.5 M thioacetamide. The obtained mixture solution was then placed at 80 °C for 24h. The final product was collected by centrifugation.

Preparation of AgCl: Silver chloride nanoparticles were synthesized according to a previous report with slight modification.[19] 350 mg of PVP and 26 mg of NaCl were dissolved in 12 ml of DMSO and kept at 60 °C for 0.5h under magnetic stirring. After that, 60 mg of CH_3COOAg were dissolved in 30 ml of DMSO and added dropwise into the above solution. The mixture was maintained at 60 °C for 1 hour. The final product was collected by centrifugation.

Preparation of Polydopamine (PDA) spheres: The synthesis of PDA spheres was based on a previous report with slight modification.[20] A solution A was prepared by mixing 2 ml of concentrated ammonia solution, 40 ml ethanol and 90 ml H_2O. Then, 0.5 g of dopamine (dissolved in 10 ml H_2O) was added in to above mixture solution and kept stirring for 24h at room temperature. The final product was collected by centrifugation.

Preparation of HKUST-1 octahedron: The synthesis of HKUST-1 can be easily found in many previous reports.[26] 60 mg of Cu $(NO_3)_2$ were dissolved in 10 ml of ethanol to form solution A. 10 mg of Trimesic acid (H_3BTC) was dissolved in another 10 ml of ethanol to form solution B. Subsequently, solution B was slowly added into A under mild stirring for 10 h. The final product was collected by centrifugation.

Preparation of MIL-96 (Al): MiL-96 (Al) was synthesized according to a previous report with some modifications.[21] 2 mmol Al $(NO_3)_3{\cdot}9H_2O$ and 1 mmol H_3BTC were dissolved in a mixture of 2 ml of H_2O and 10 ml of DMF. The obtained mixture solution was then transferred into a 20 ml Teflon-lined stainless autoclave and maintained at 160 °C for 24 h. After it cooled to room temperature, the final product was collected by centrifugation.

Preparation of Hollow SiO_2 spheres: The hollow SiO_2 spheres were prepared according to a previous report.[22] 1. 08 g of CTAB was dissolved in a mixture of 380 ml of H_2O and 125 ml of ethanol. Then, 1ml of ammonia aqueous solution and 1 ml of TEOS were added to form a clear solution. The resulting solution was maintained at 35 °C under mild stirring for 24h. The silica spheres were collected by centrifugation. Hollow spheres were synthesized by incubated the obtained silica sphere in 160 ml of H_2O at 50 °C for 24 h and then isolated by centrifugation.

1.3 Characterization

The powder X-ray diffraction (XRD) patterns were obtained using a STOE MP diffractometer in transmission mode with Mo Kα radiation (λ=0.07093 nm). The microstructure of the samples was studied by transmission electron microscopy on a Philips CM200 and a FEI Talos F200S. A Phenom Pharos Desktop SEM was applied to test morphology of samples. FT-IR spectra were recorded using

a Nicolet iS5 FTIR Spectrometer from Thermo Scientific. The Raman spectra were acquired on an XPLORA plus Raman microscope with a 532 nm laser. The battery performance was tested by using a CT2001A battery system (Land Instruments) at room temperature. The N_2 adsorption-desorption isotherm and the CO_2 sorption isotherm was tested on a Micromeritics ASAP instrument. X-ray total scattering information was obtained at beamline P02.1, PETRA III,[24] located at the Deutsches Elektronen-Synchrotron (DESY) in Hamburg, Germany. All samples were measured for 10 min in full ring configuration on a VAREX XRD 4343CT detector at a sample detector distance of 290 mm and a wavelength of 0.2073 Å. Patterns were integrated using pyFAI[25] and transformed using xPDFsuite based on the PDFgetX3 engine.[26] A Q_{max} of 18.6 Å^{-1} was achieved.

1.4 Electrochemical test

All electrochemical measurements were conducted employing coin cells (CR 2032) type half cells. Si@C or Si as active material, carbon black and polyvinylidene fluoride (PVDF) were mixed with N-methyl-2-pyrrolidone (NMP) with mass ratio of 7:2:1 to fabricate the working electrode. Then, the slurry was spread onto a copper foil. The mass loading was around 1 mg cm^{-2}. Lithium metal foil was used as both counter and reference electrodes. The electrolyte used was 1 M $LiPF_6$ dissolved in a mixture of ethylene carbonate, diethyl carbonate, and dimethyl carbonate with 1:1:1 volume ratio. Glass fibers from Whatman were employed as a separator.

2. Supplementary Figures and Tables

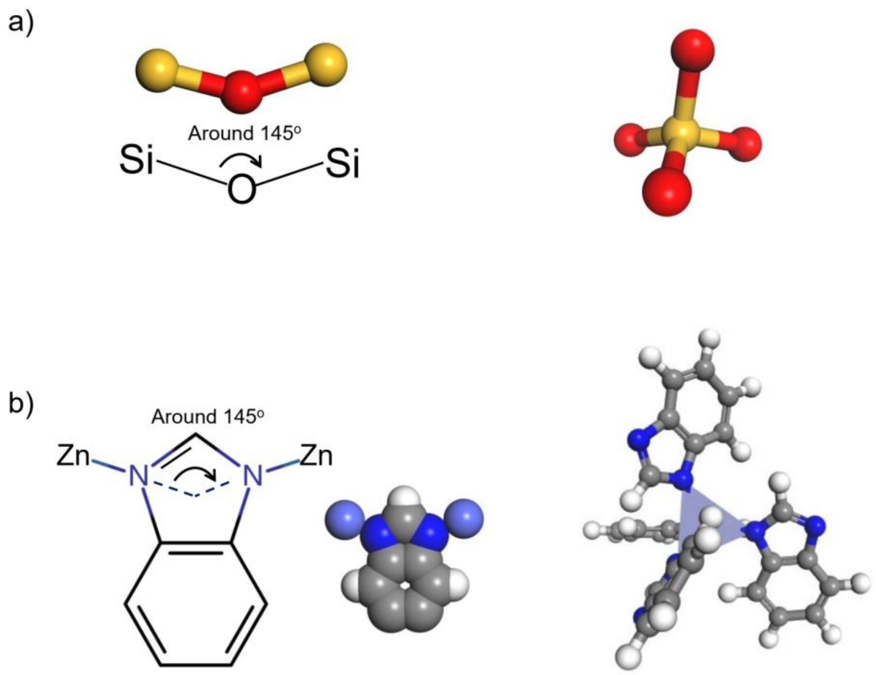

Supplementary Figure 1. (a) Schematic illustration of SiO_2 local structure. (b) Schematic illustration of ZIF-7 structure. A Zn atom is bonded by four benzimidazole molecules.

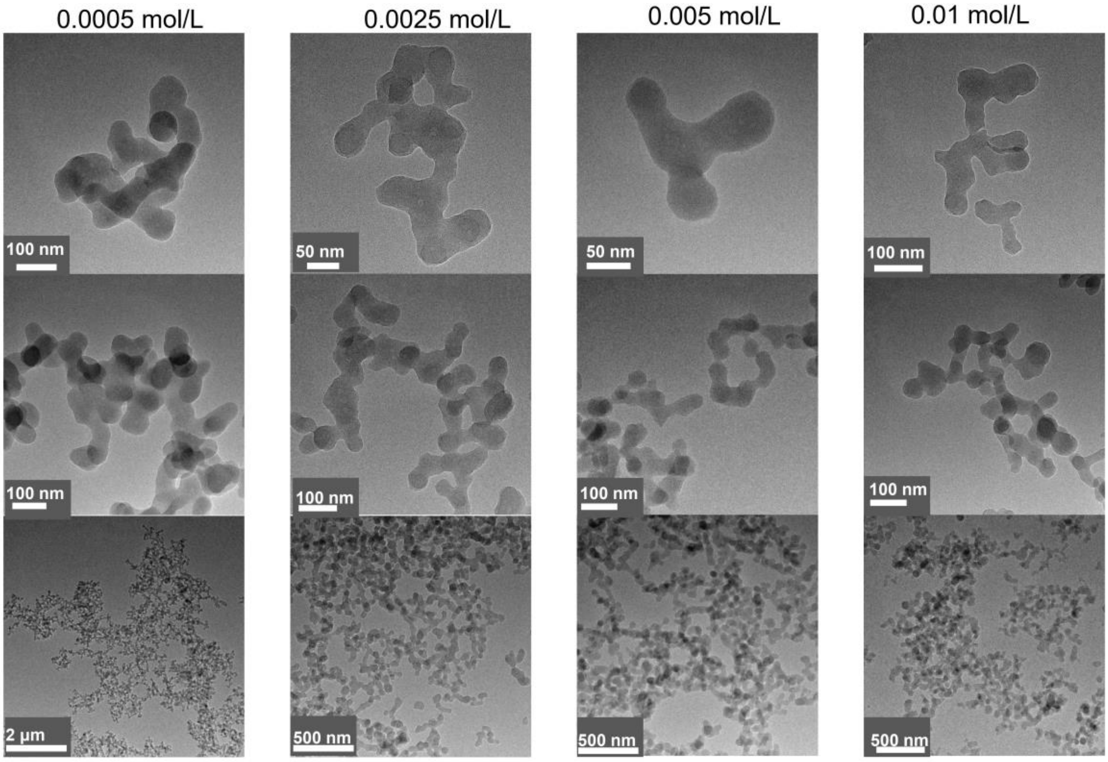

Supplementary Figure 2. TEM images of amorphous ZIF-7 obtained by adding directly TEA to the mother solution. The TEA concentration is varied from 0.0005 to 0.01 mol L^{-1}.

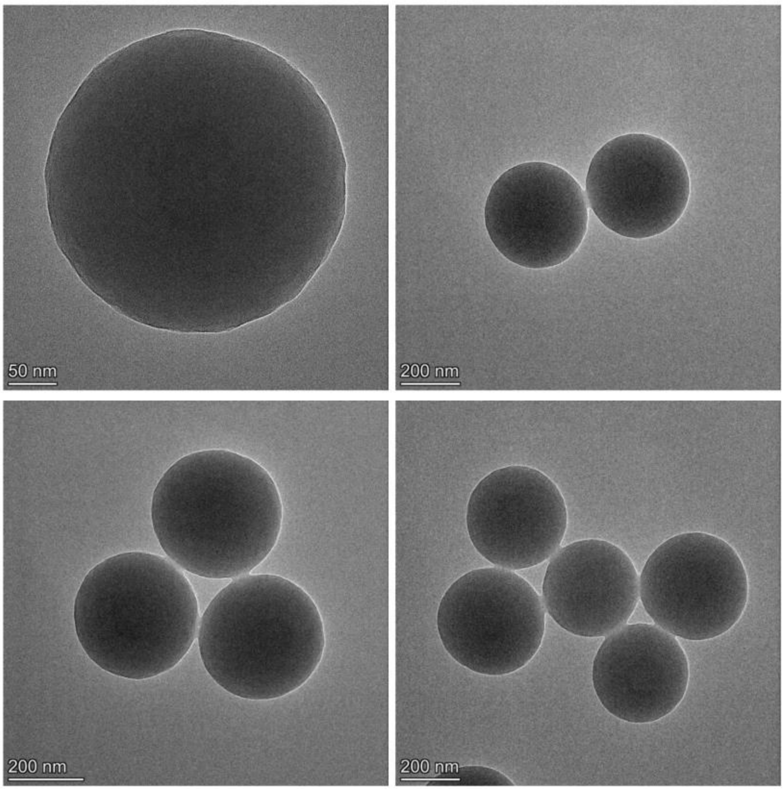

Supplementary Figure 3. TEM images of amorphous ZIF-7 with different magnifications obtained by TEA vapor diffusion method.

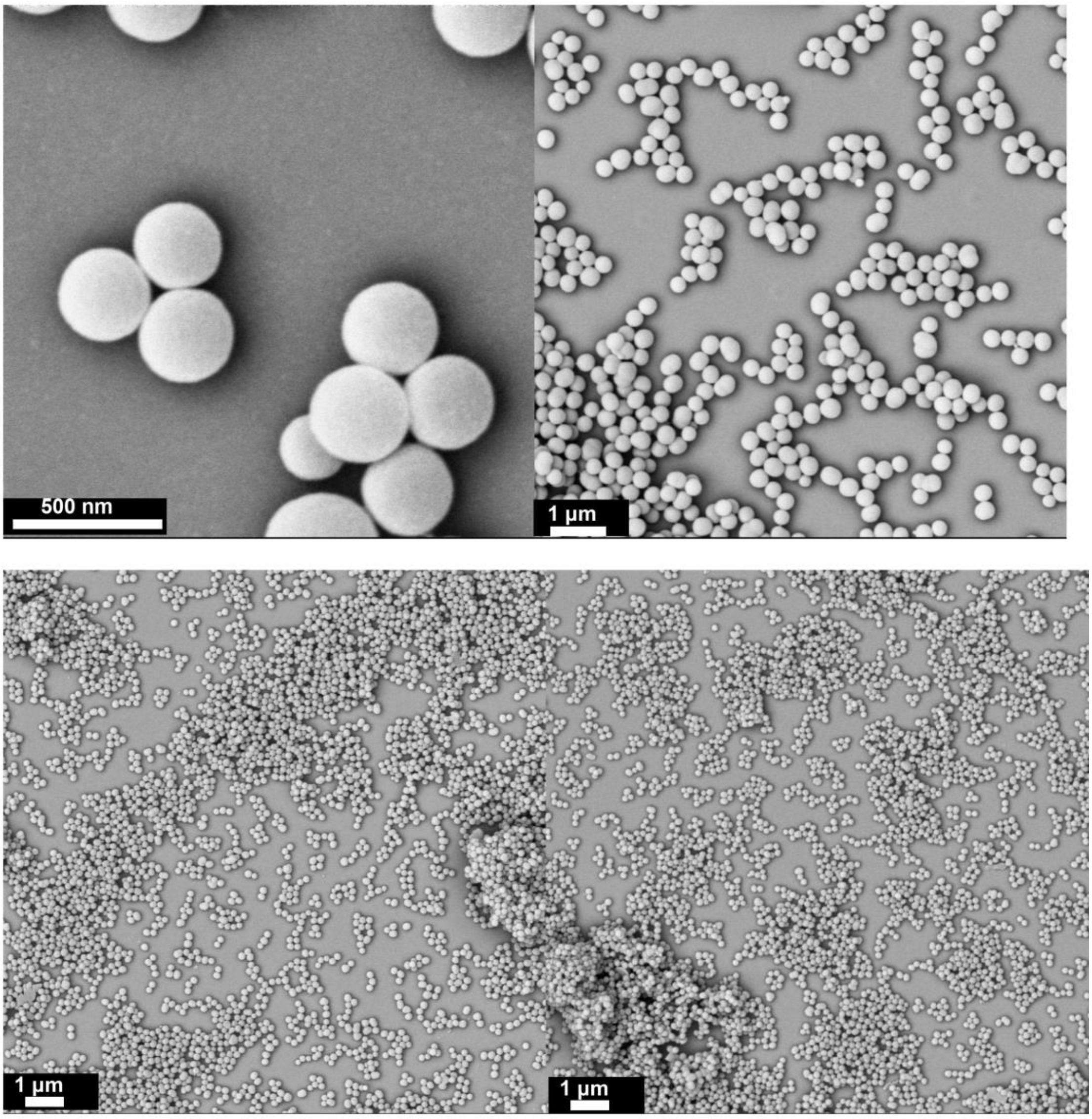

Supplementary Figure 4. SEM images of amorphous ZIF-7 with different magnifications obtained by TEA vapor diffusion method.

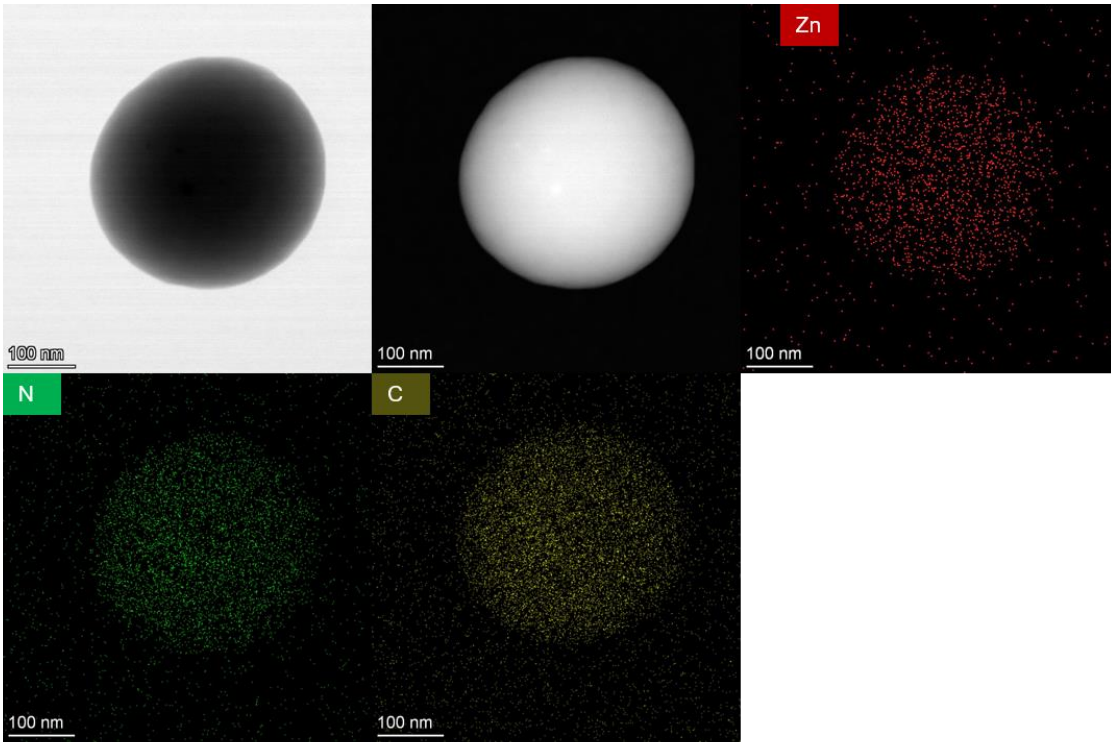

Supplementary Figure 5. Bright Field STEM image, HAADF-STEM image and element mapping images of a-ZIF-7 spheres.

a)

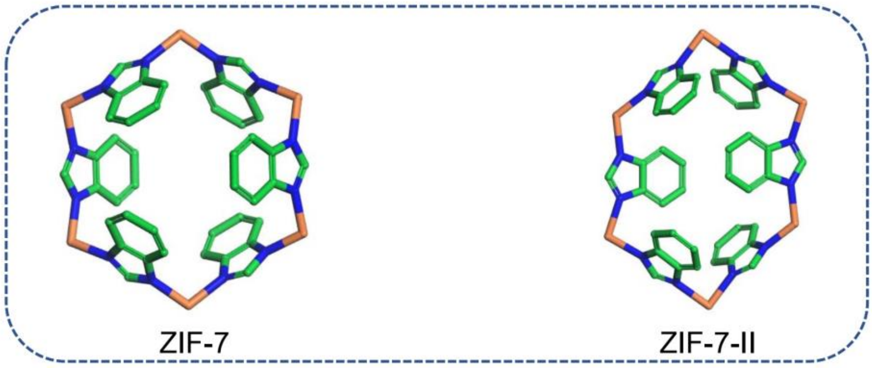

b)

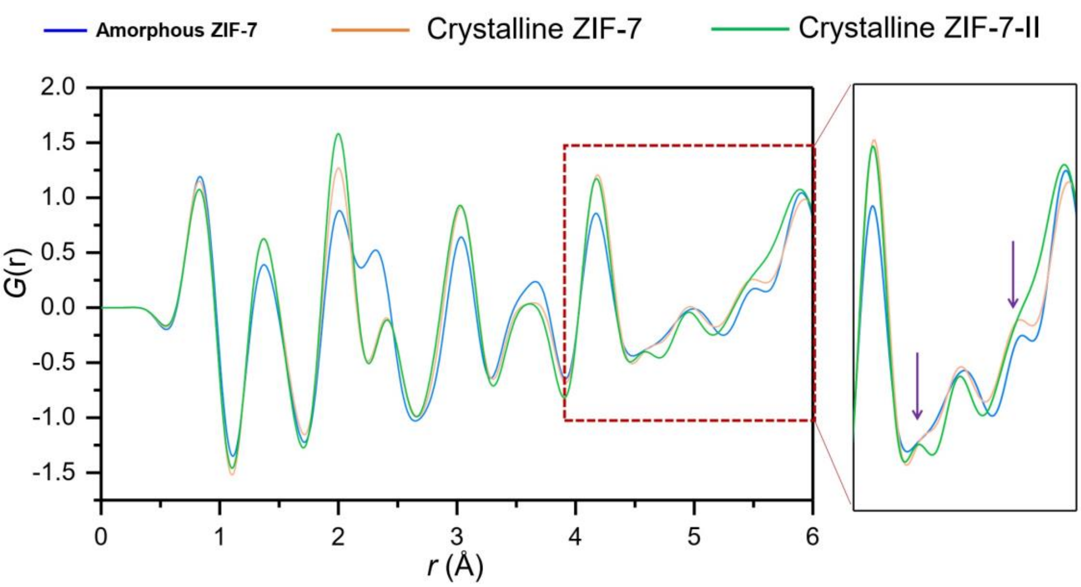

Supplementary Figure 6. (a) Simplified representation of the ZIF-7 and ZIF-II crystal structure. (b) Experimental PDF of amorphous ZIF-7, crystalline ZIF-7 and crystalline ZIF-7-II.

The ZIF-7 and ZIF-7-II share some chemical compositions, but quite different crystal structure. Compared with ZIF-7-II, a much better fit of the local structure is observed for the ZIF-7 structure. There is an almost 100% match of peaks up to 6Å.

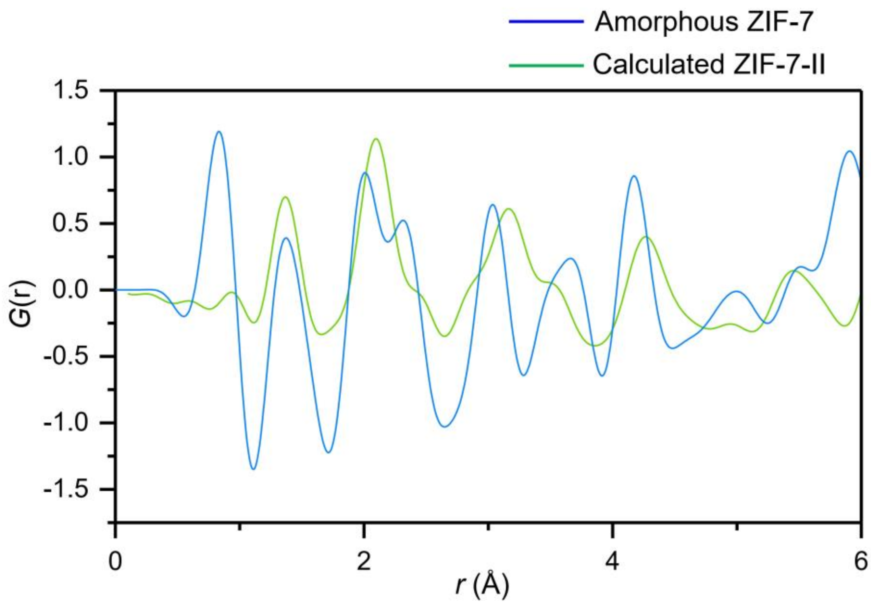

Supplementary Figure 7. Experimental PDF of amorphous ZIF-7 and calculated patterns of atom-pair distance distribution of ZIF-7-II.

While some peaks do match between the experimental and the calculated pattern of ZIF-7-II. The overall match is relatively poor.

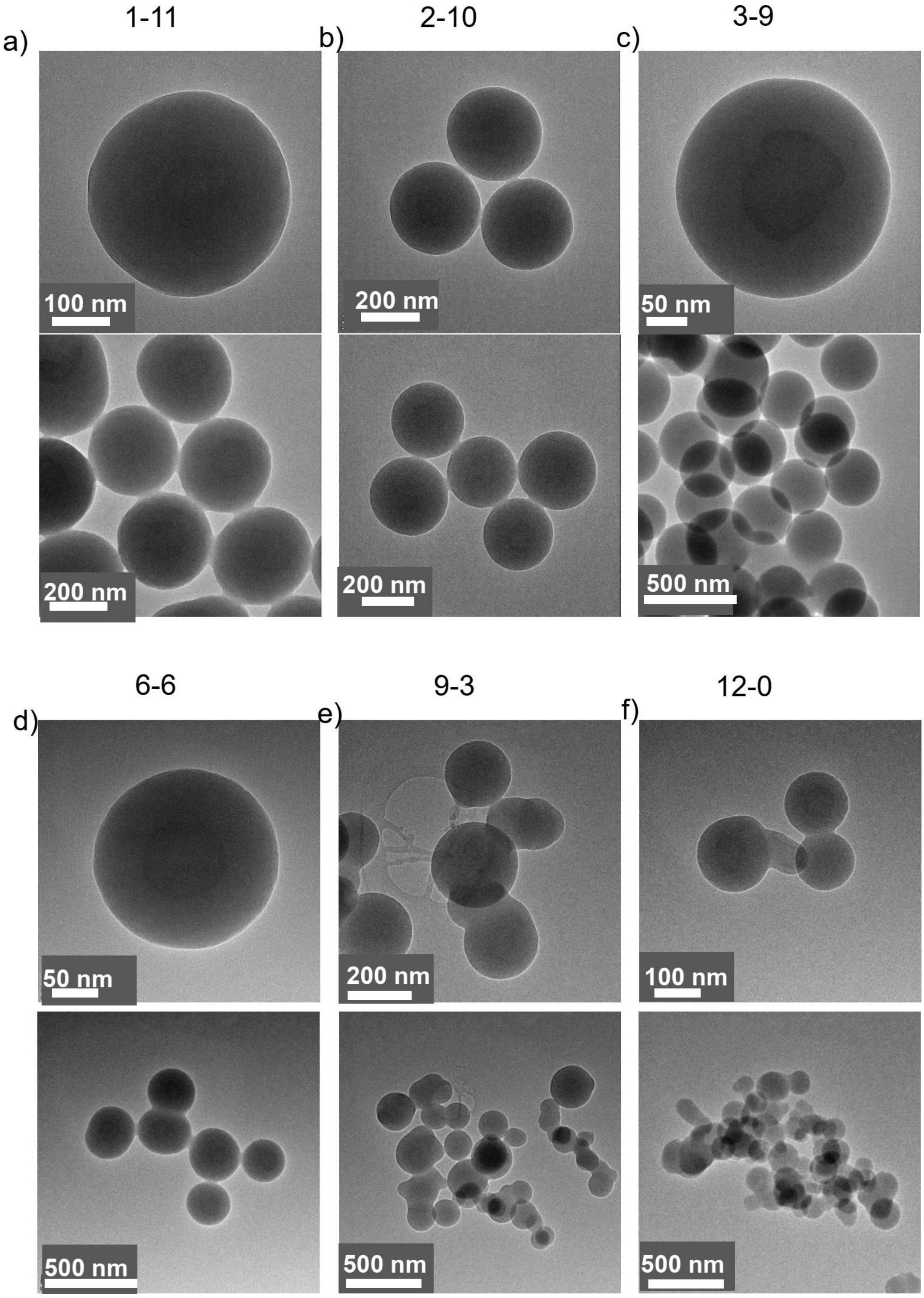

Supplementary Figure 8. TEM images of amorphous ZIF-7 using different ratios of TEA to EtOH. (a) 1:11. (b) 2:10. (c) 3:9. (d) 6:6. (e) 9:3. (f) 12:9.

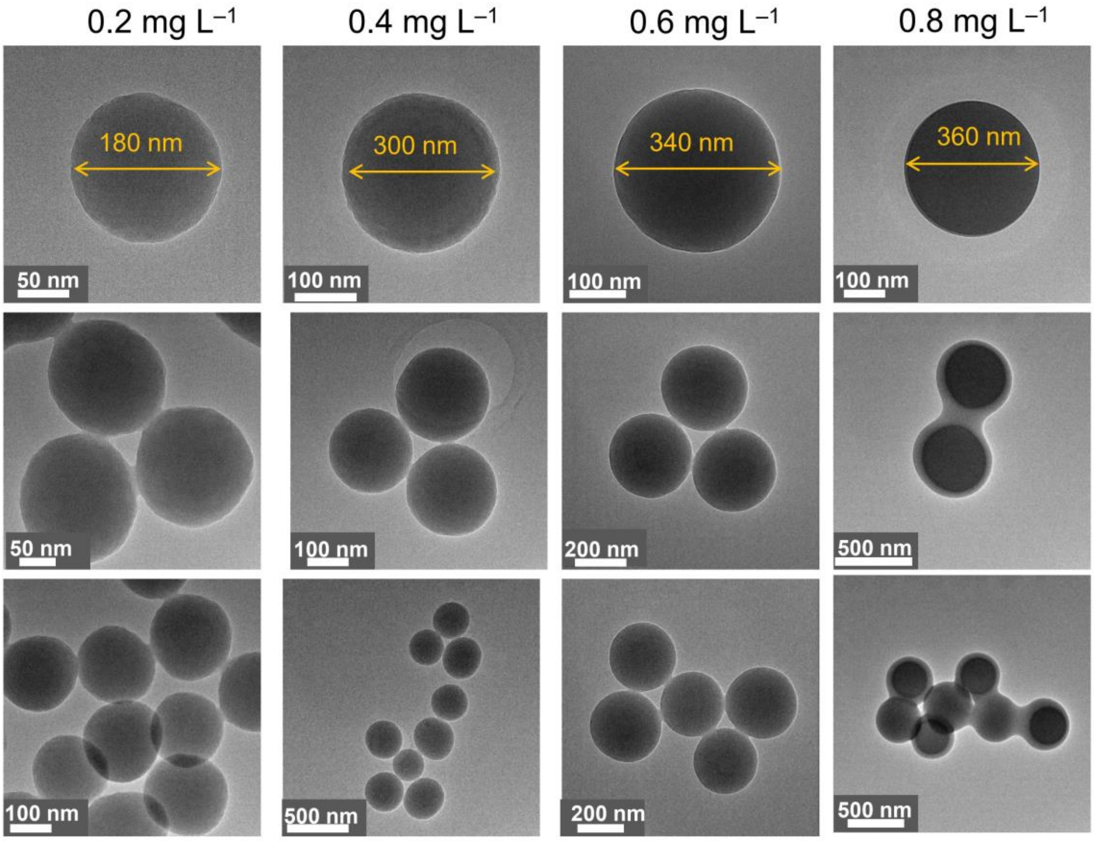

Supplementary Figure 9. TEM images of amorphous ZIF-7 obtained by varying the concentration of $ZnCl_2$ from 0.2 to 0.8 mg L^{-1}.

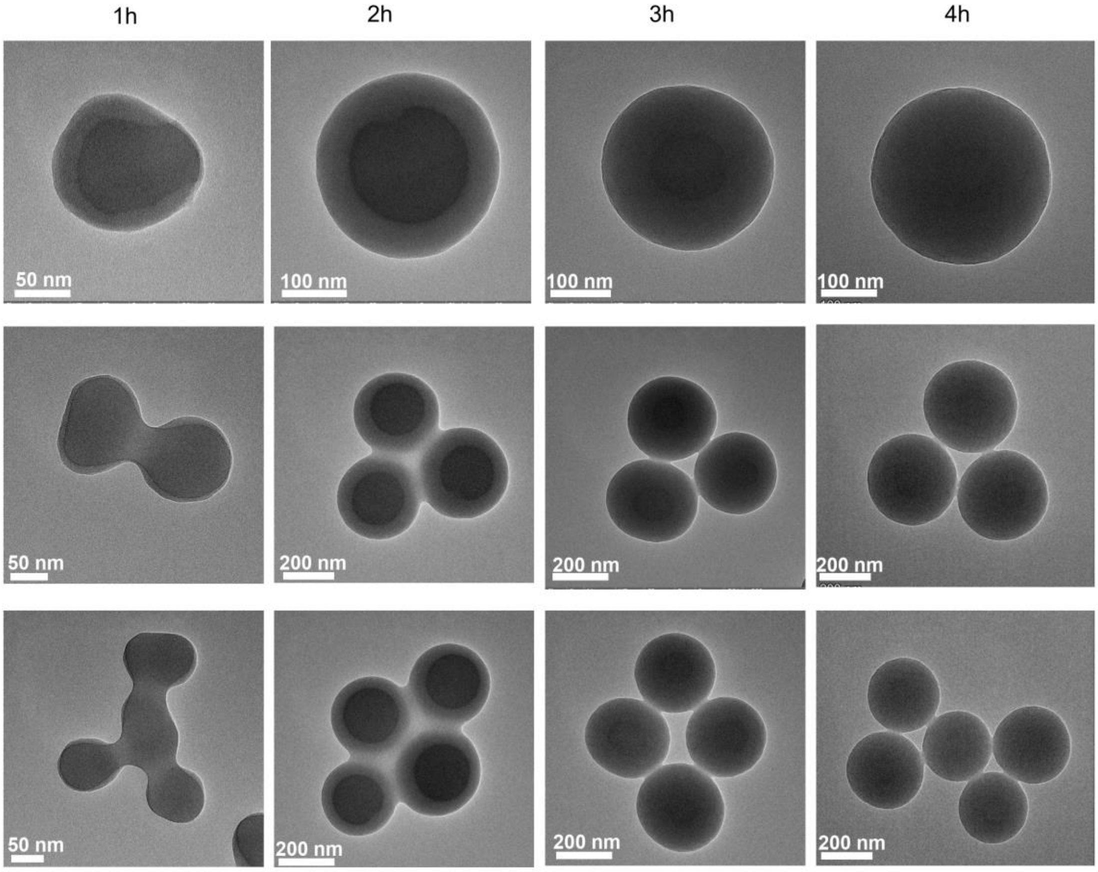

Supplementary Figure 10. Time series of ex-situ TEM images displaying the formation of aZIF-7 sphere.

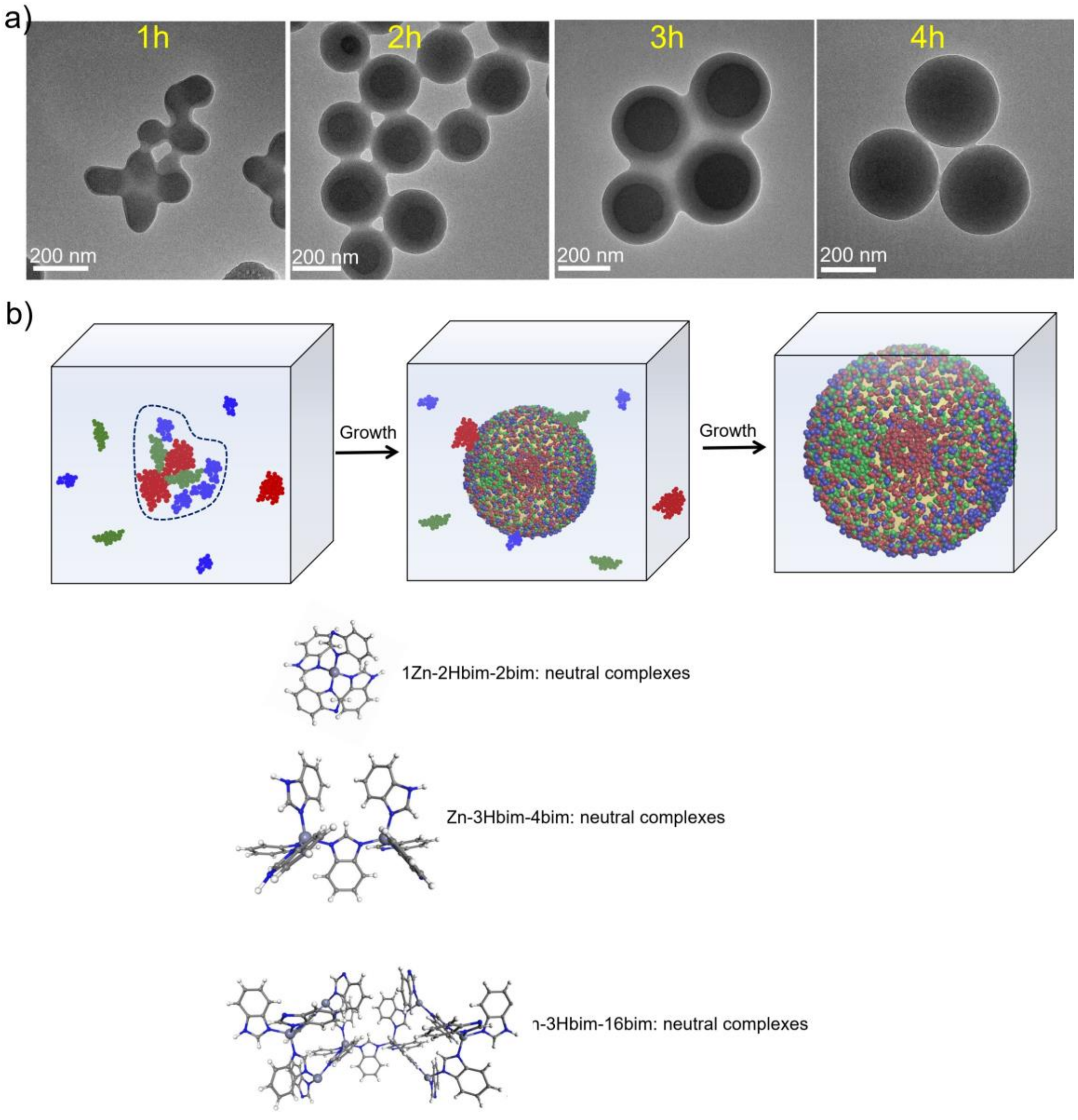

Supplementary Figure 11. (a) Time series of ex-situ TEM images displaying the formation of aZIF-7 sphere. (b) Schematic illustration of formation process for aZIF-7 spheres.

Different colors represent varying sizes of Zn-benzimidazole complexes. In the formation of aZIF-7 spheres, diverse Zn-benzimidazole complexes with different sizes could be generated first and then attached onto surface of the existing particles. The three Zn-benzimidazole complexes are just listed for schematic illustration of this process.

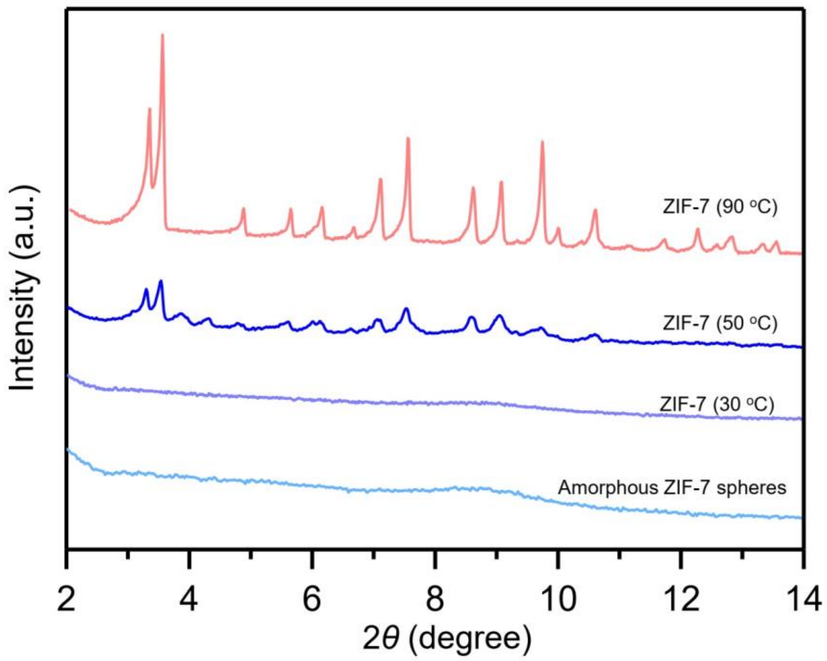

Supplementary Figure 12. PXRD patterns of amorphous ZIF-7 spheres incubated at temperatures of 30, 50 and 90 °C, respectively.

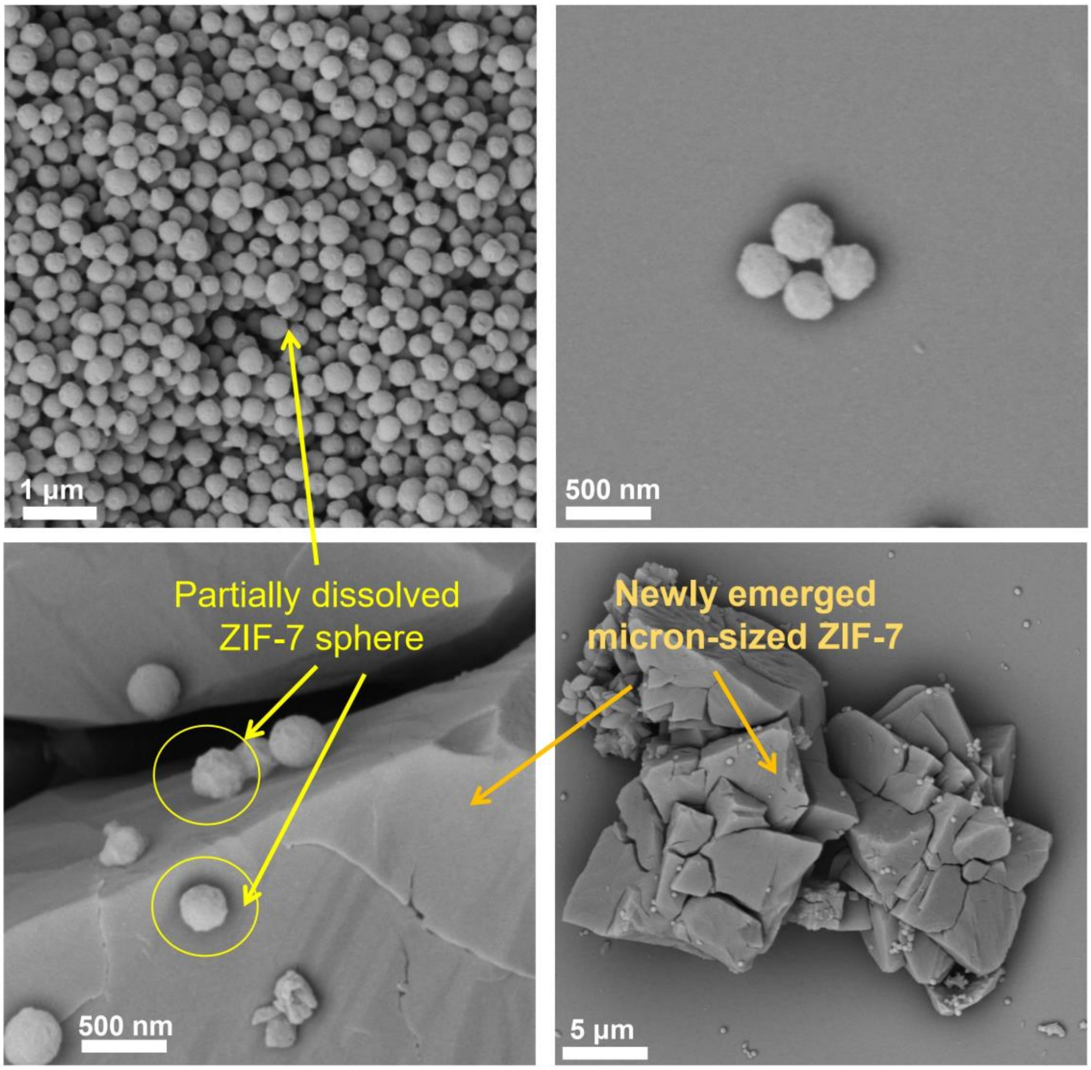

Supplementary Figure 13. SEM images of a-ZIF-7 spheres incubated at temperatures of 50°C for 24h with different magnifications.

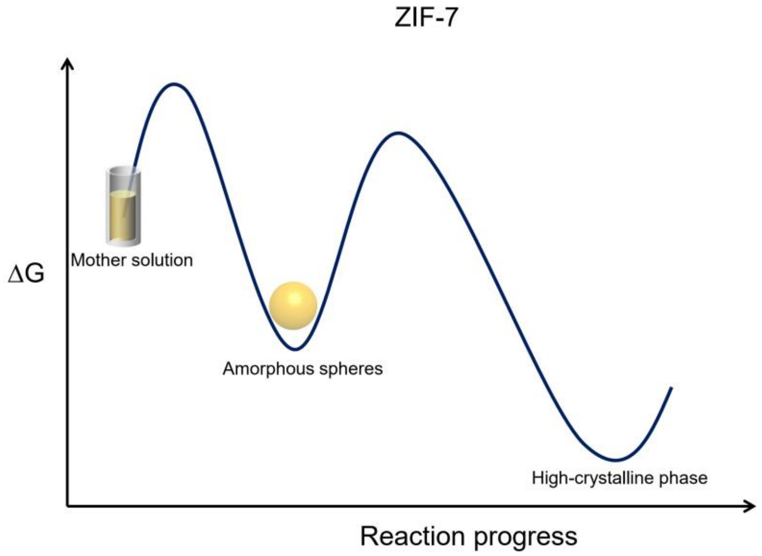

Supplementary Figure 14. Schematic illustration of formation of amorphous ZIF-7 under kinetic control and high-crystalline ZIF-7 under thermodynamic control.

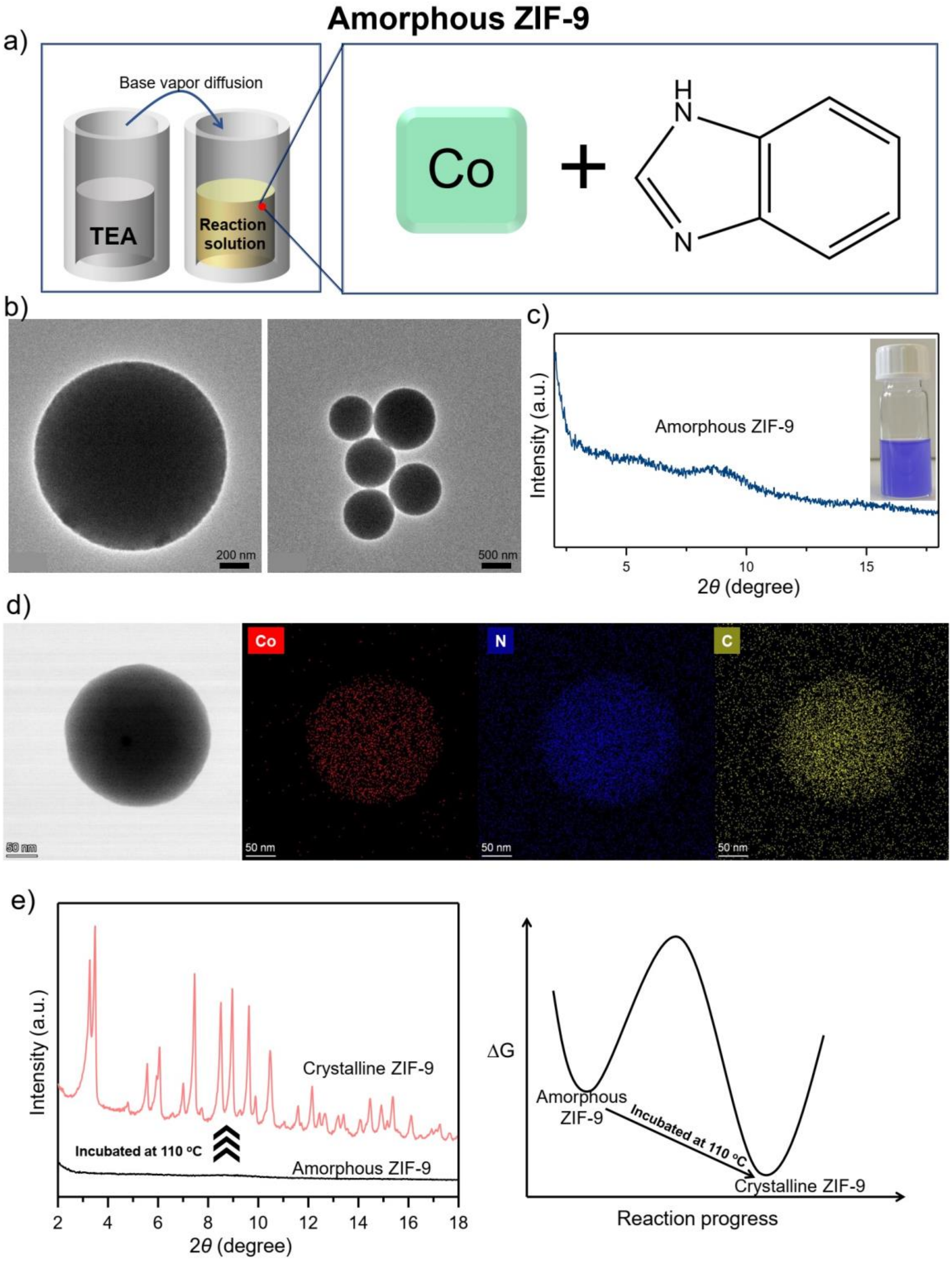

Supplementary Figure 15. (a) Schematic illustration of the preparation of ZIF-9 using the TEA diffusion method. (b)TEM images of a-ZIF-9 spheres. (c) PXRD pattern of a-ZIF-9 spheres, with an inset showing an optical image of a-ZIF-9 colloidal solution. (d) TEM images and element mapping images of a-ZIF-9 spheres. Co, N, and C elements are uniformly distributed. (e) PXRD patterns of amorphous ZIF-9 spheres incubated at 110 °C. Elevated temperatures trigger the transition of ZIF-9 from an amorphous to a crystalline state, indicating the influence of thermodynamic control.

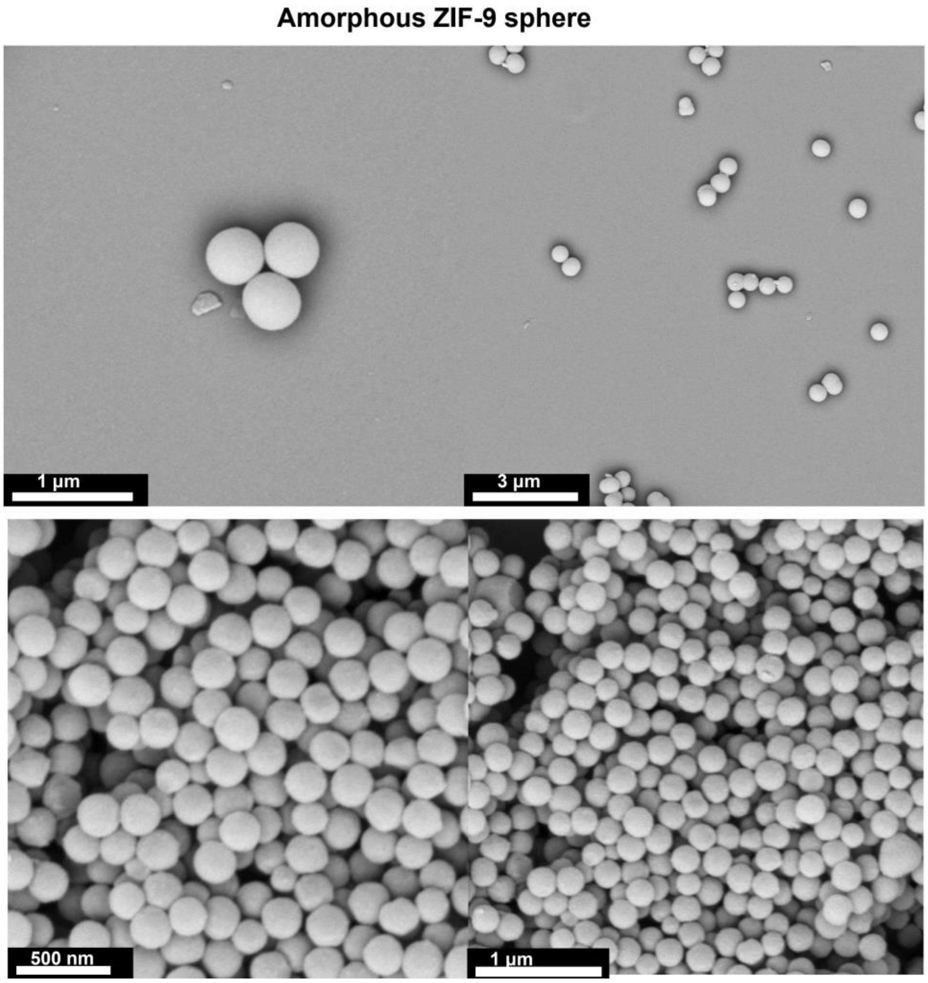

Supplementary Figure 16. SEM images of amorphous ZIF-9 with different magnifications obtained by TEA vapor diffusion method.

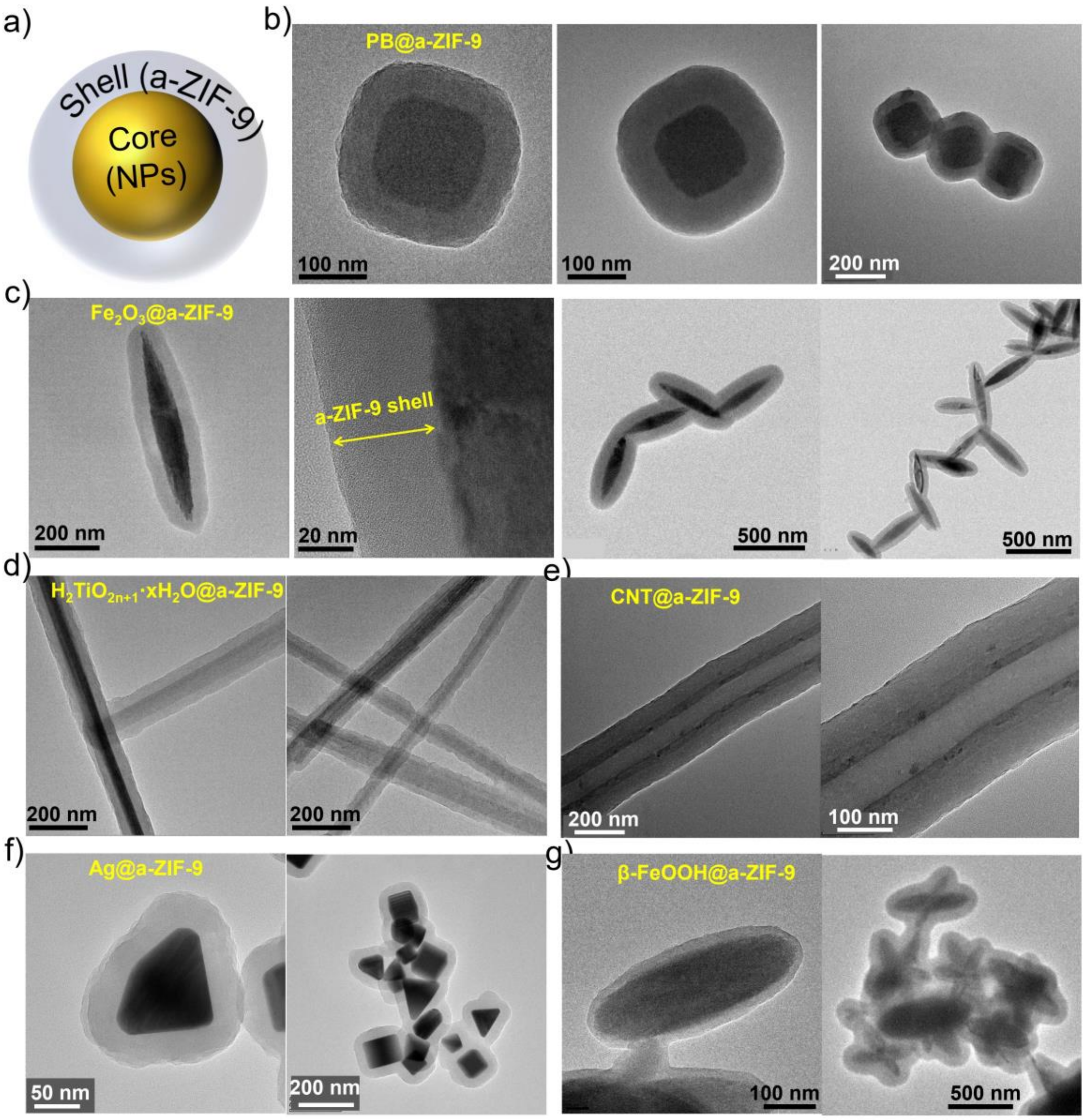

Supplementary Figure 17. (a) Schematic illustration of core-shell colloids with amorphous ZIF-9 as the shell. (b)TEM images of PB@a-ZIF-9 colloids. (c) TEM images of Fe_2O_3@a-ZIF-9 colloids. (d) TEM images of $H_2TiO_{2n+1}\cdot xH_2O$@a-ZIF-9. (e) TEM images of CNT@a-ZIF-9 colloids. (f) TEM images of Ag@a-ZIF-9 colloids. (g) TEM images of β-FeOOH@a-ZIF-9 colloids.

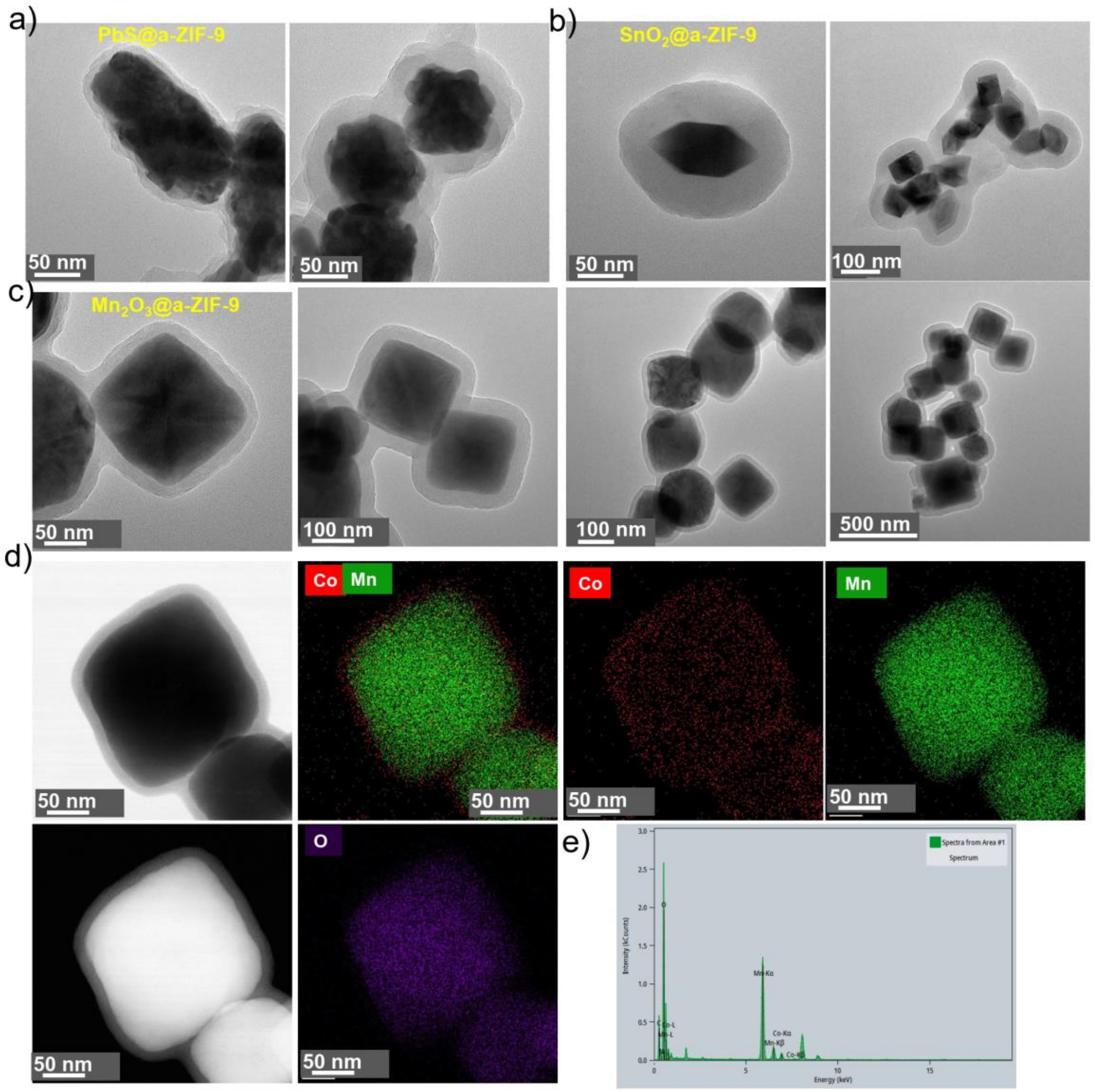

Supplementary Figure 18. (a) TEM images of PbS@a-ZIF-9 colloids. (b) TEM images of SnO_2@a-ZIF-9 colloids. (c) TEM images of Mn_2O_3@a-ZIF-9 colloids. (d) TEM images and element mapping images of Mn_2O_3@a-ZIF-9 colloids. Co are uniformly distributed around Mn elements. (e) Representative energy dispersive X-ray (EDX) spectrum for Mn_2O_3@a-ZIF-9.

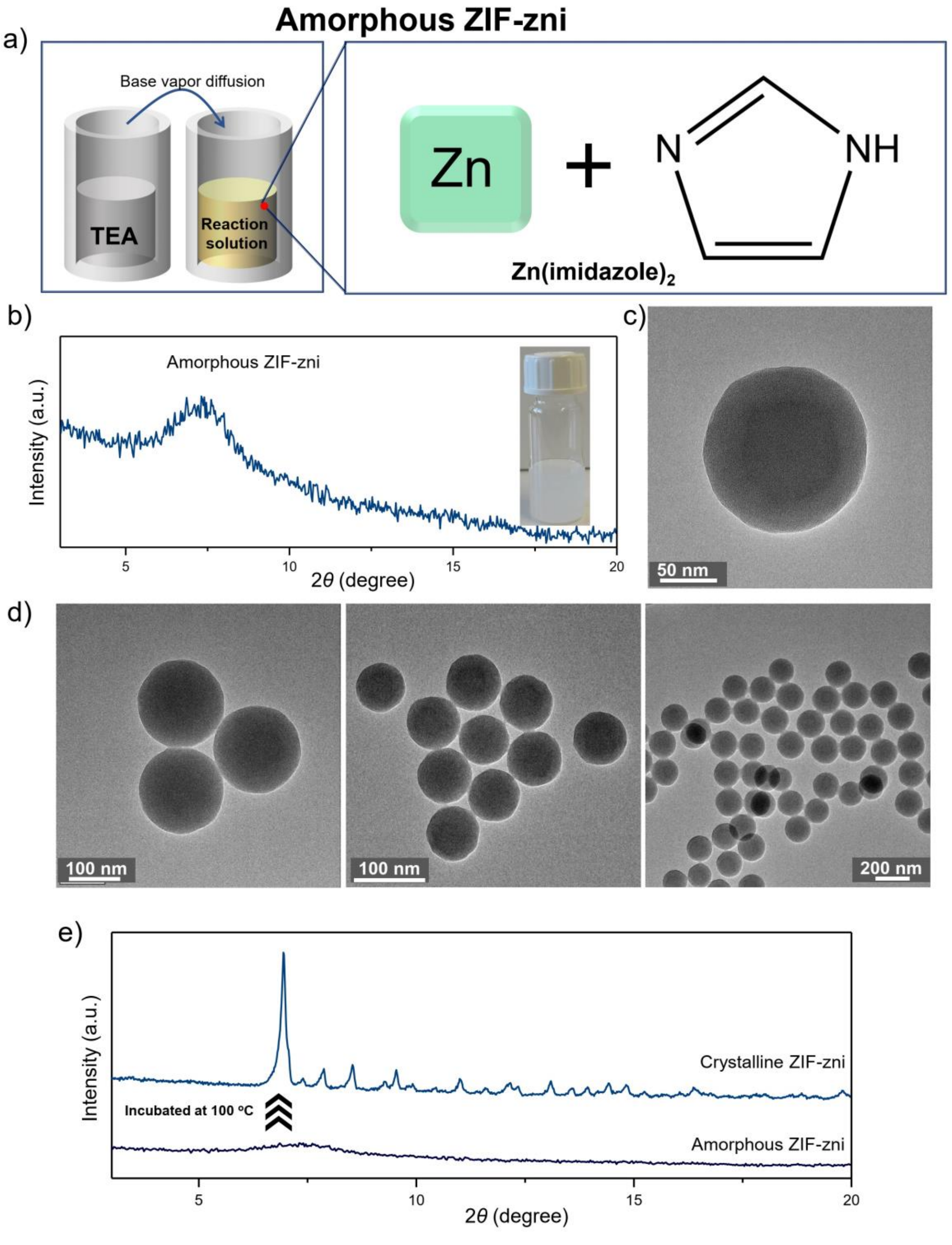

Supplementary Figure 19. (a) Schematic illustration of the preparation of ZIF-zni ($Zn(imidazole)_2$) using the TEA diffusion method. (b) PXRD pattern of a-ZIF-zni spheres, with an inset showing an optical image of the a-ZIF-zni colloidal solution. (c-d) TEM images of a-ZIF-zni spheres at different magnifications. (c) PXRD patterns of amorphous ZIF-zni spheres incubated at 100 °C. High temperatures induce the transition from an amorphous to a crystalline state, indicating the influence of thermodynamic control.

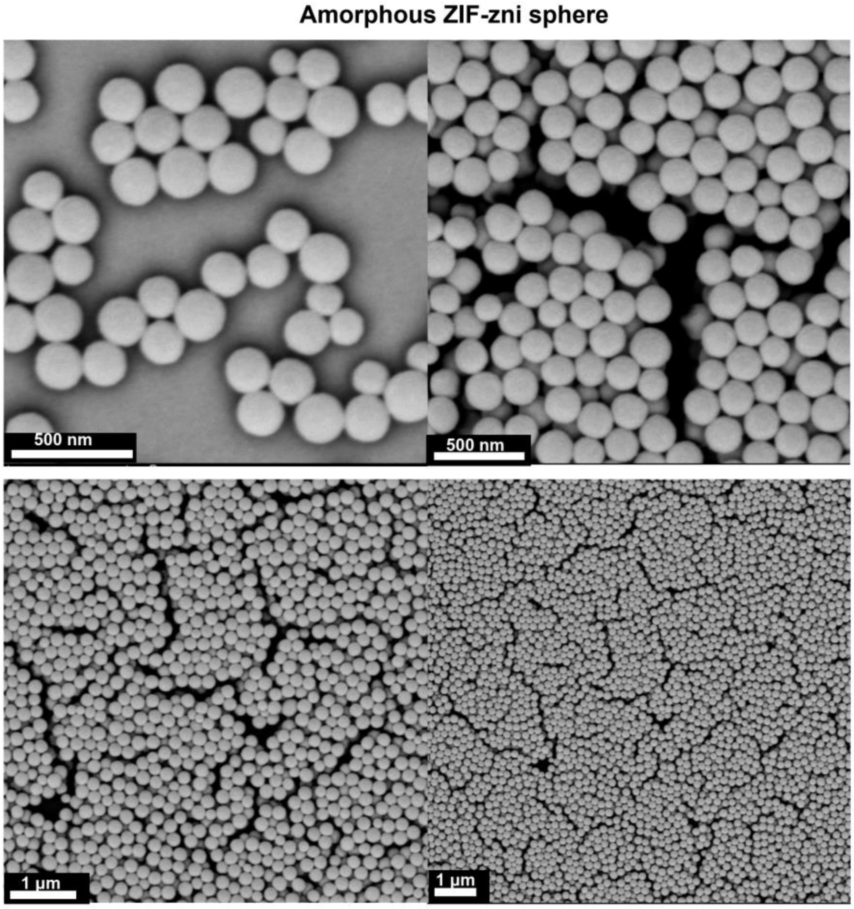

Supplementary Figure 20. SEM images of amorphous ZIF-zni with different magnifications obtained by TEA vapor diffusion method.

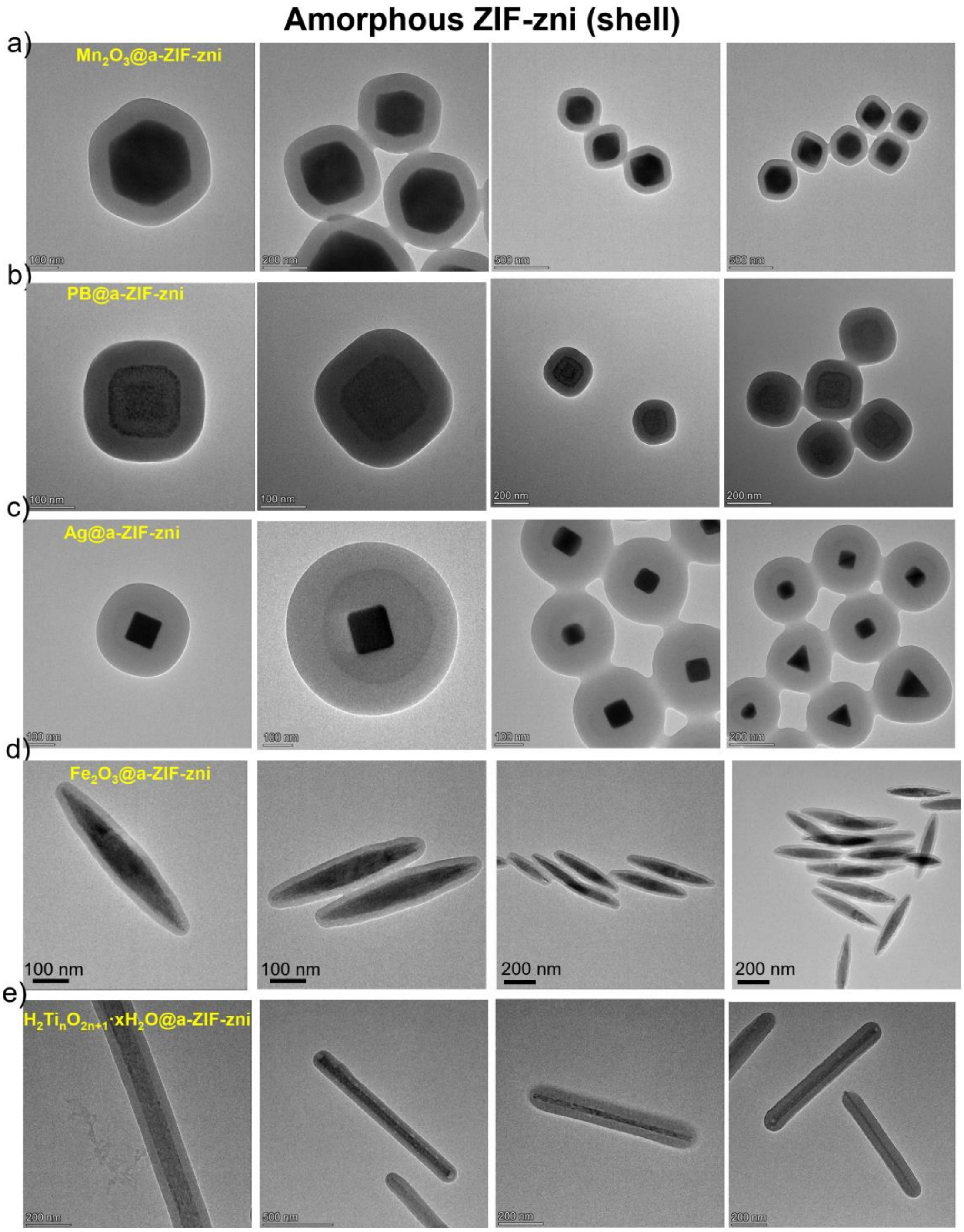

Supplementary Figure 21. (a) TEM images of Mn_2O_3@a-ZIF-zni. (b) TEM images of PB@a-ZIF-zni. (c) TEM images of Ag@a-ZIF-zni. (d) TEM images of Fe_2O_3@a-ZIF-zni. (e) TEM images of $H_2TiO_{2n+1}·xH_2O$@a-ZIF-zni at different magnifications.

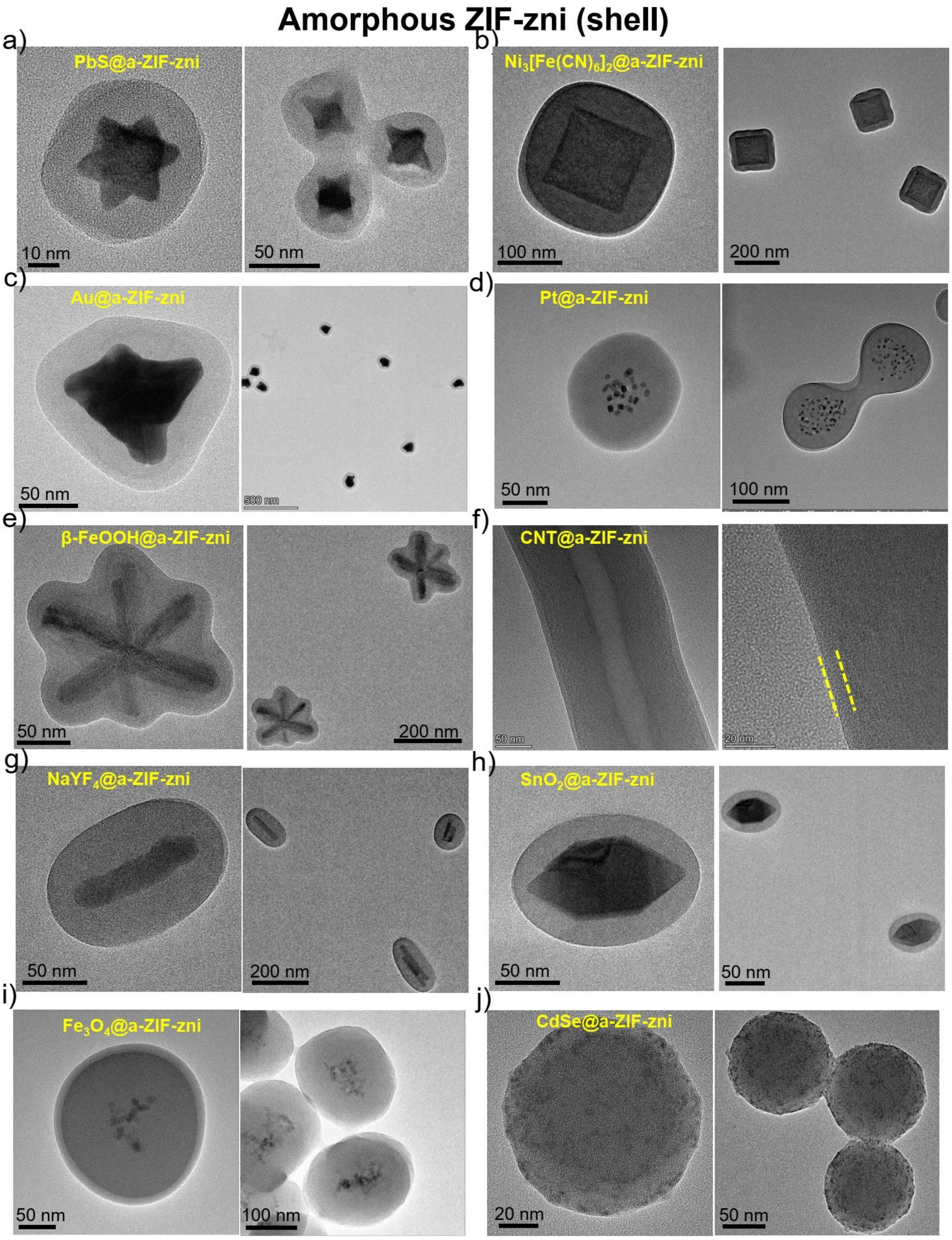

Supplementary Figure 22. (a) TEM images of PbS@a-ZIF-zni . (b) TEM images of $Ni_3[Fe(CN)_6]_2$@a-ZIF-zni . (c) TEM images of Au@a-ZIF-zni . (d) TEM images of Pt@a-ZIF-zni. (e) TEM images of β-FeOOH@a-ZIF-zni. (f) TEM images of CNT@a-ZIF-zni. (g) TEM images of $NaYF_4$@a-ZIF-zni. (h) TEM images of SnO_2@a-ZIF-zni. (i) TEM images of Fe_3O_4@a-ZIF-zni. (j) TEM images of CdSe@a-ZIF-zni at different magnifications.

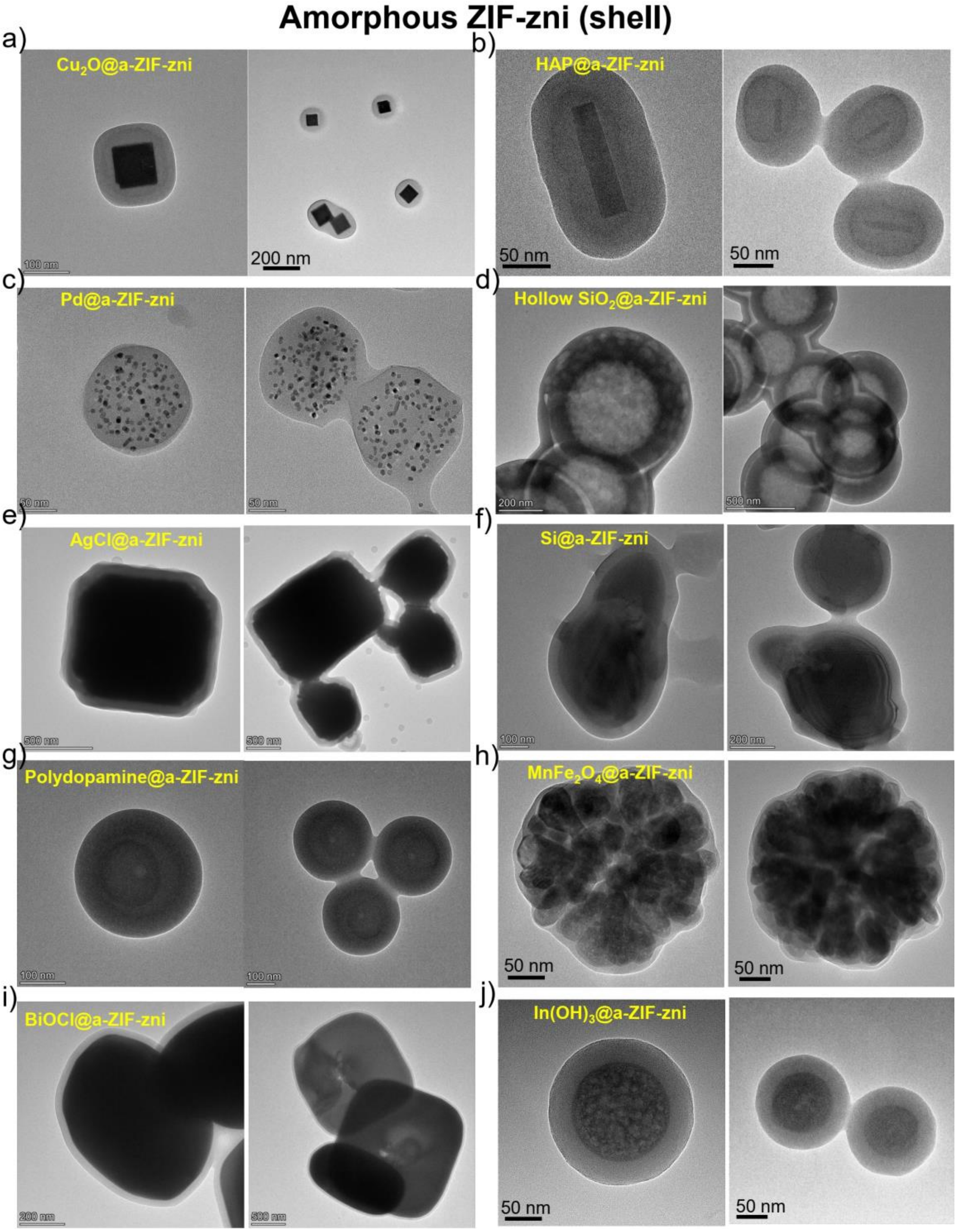

Supplementary Figure 23. (a) TEM images of Cu_2O@a-ZIF-zni. (b) TEM images of HAP@a-ZIF-zni. Hydroxyapatite (HAP) is $Ca_5(PO_4)_3OH$. (c) TEM images of Pd@a-ZIF-zni. (d) TEM images of Hollow SiO_2@a-ZIF-zni. (e) TEM images of AgCl@a-ZIF-zni. (f) TEM images of Si@a-ZIF-zni. (g) TEM images of Polydopamine@a-ZIF-zni. (h) TEM images of $MnFe_2O_4$@a-ZIF-zni. (i) TEM images of BiOCl@a-ZIF-zni. (j) TEM images of $In(OH)_3$@a-ZIF-zni.

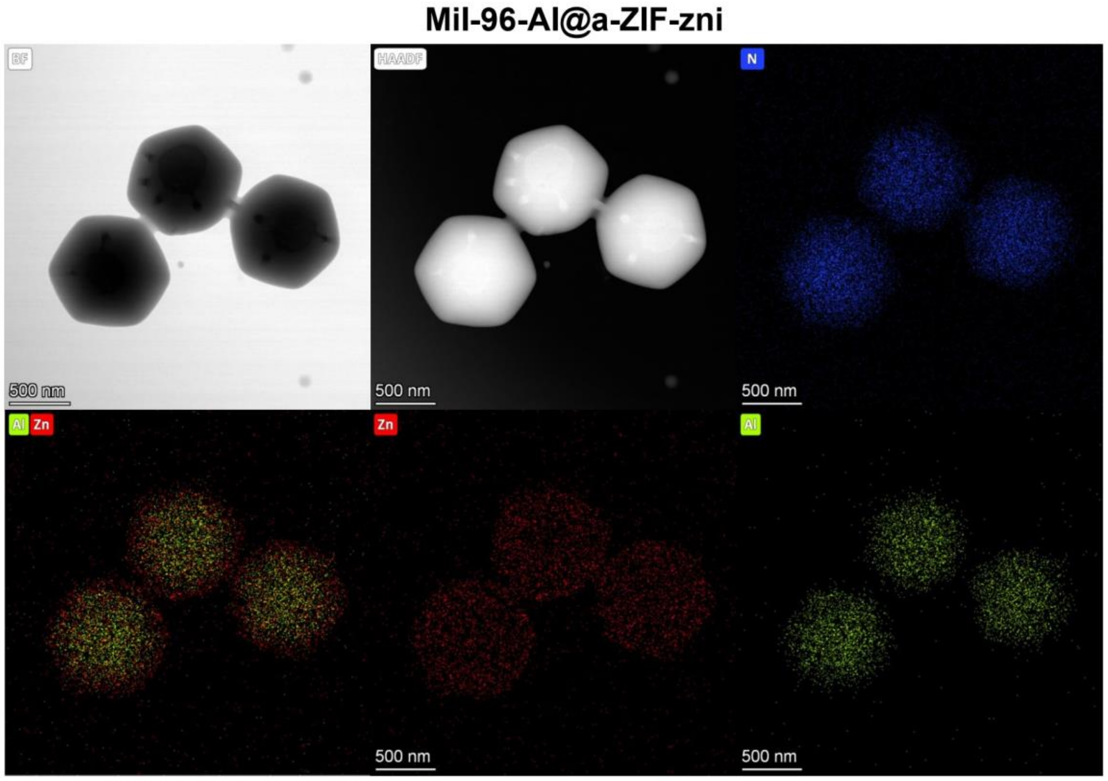

Supplementary Figure 24. TEM images and element mapping images of MIL-96-Al@ a-ZIF-zni. Zn are uniformly distributed around MIL-96 nanoparticles.

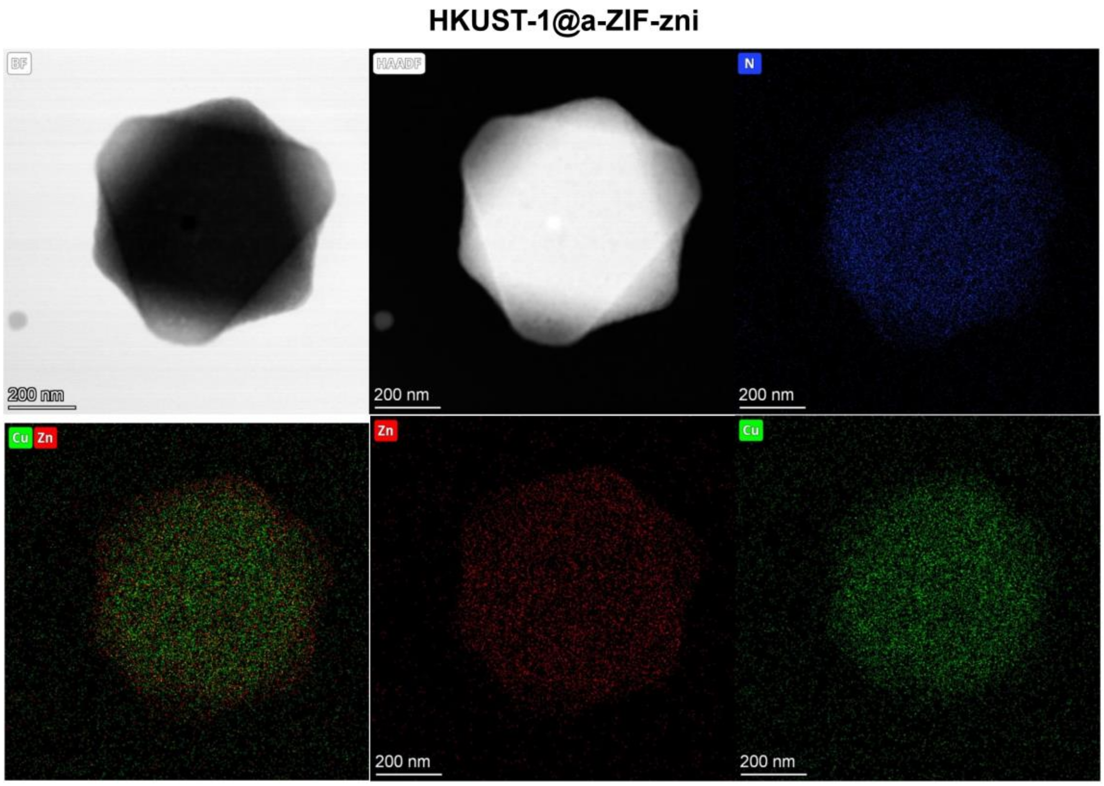

Supplementary Figure 25. TEM images and element mapping images of HKUST-1@ a-ZIF-zni. Zn are uniformly distributed around HKUST-1.

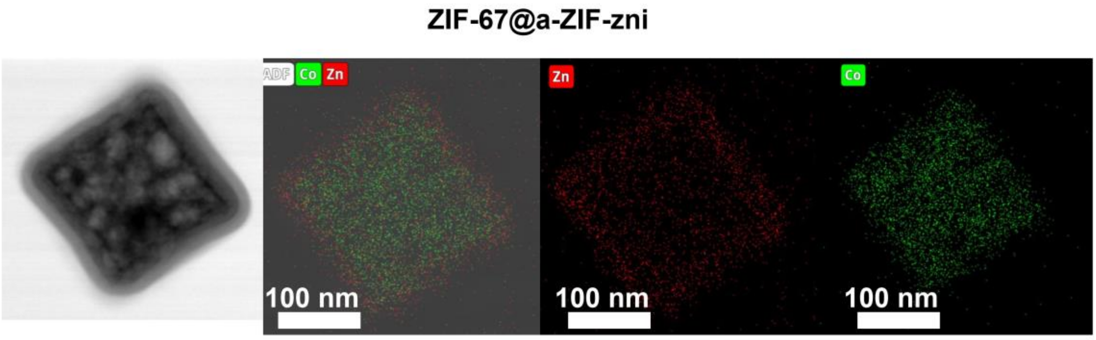

Supplementary Figure 26. TEM images and element mapping images of ZIF-67@ a-ZIF-zni. Zn are uniformly distributed around ZIF-67.

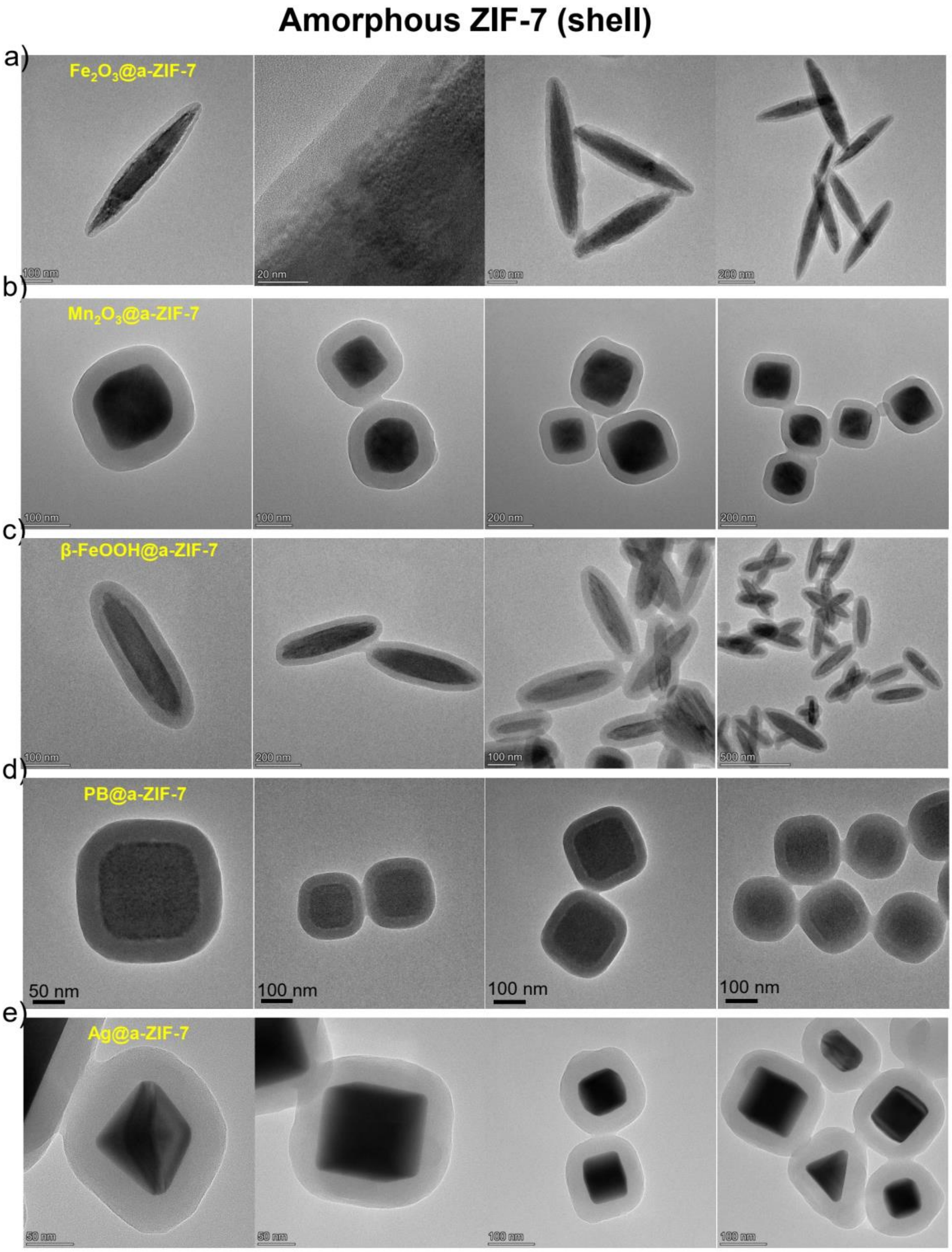

Supplementary Figure 27. (a) TEM images of Fe_2O_3@a-ZIF-7. (b) TEM images of Mn_2O_3@a-ZIF-7. (c) TEM images of β-FeOOH @a-ZIF-7. (d) TEM images of PB@a-ZIF-7. (e) TEM images of Ag@a-ZIF-7.

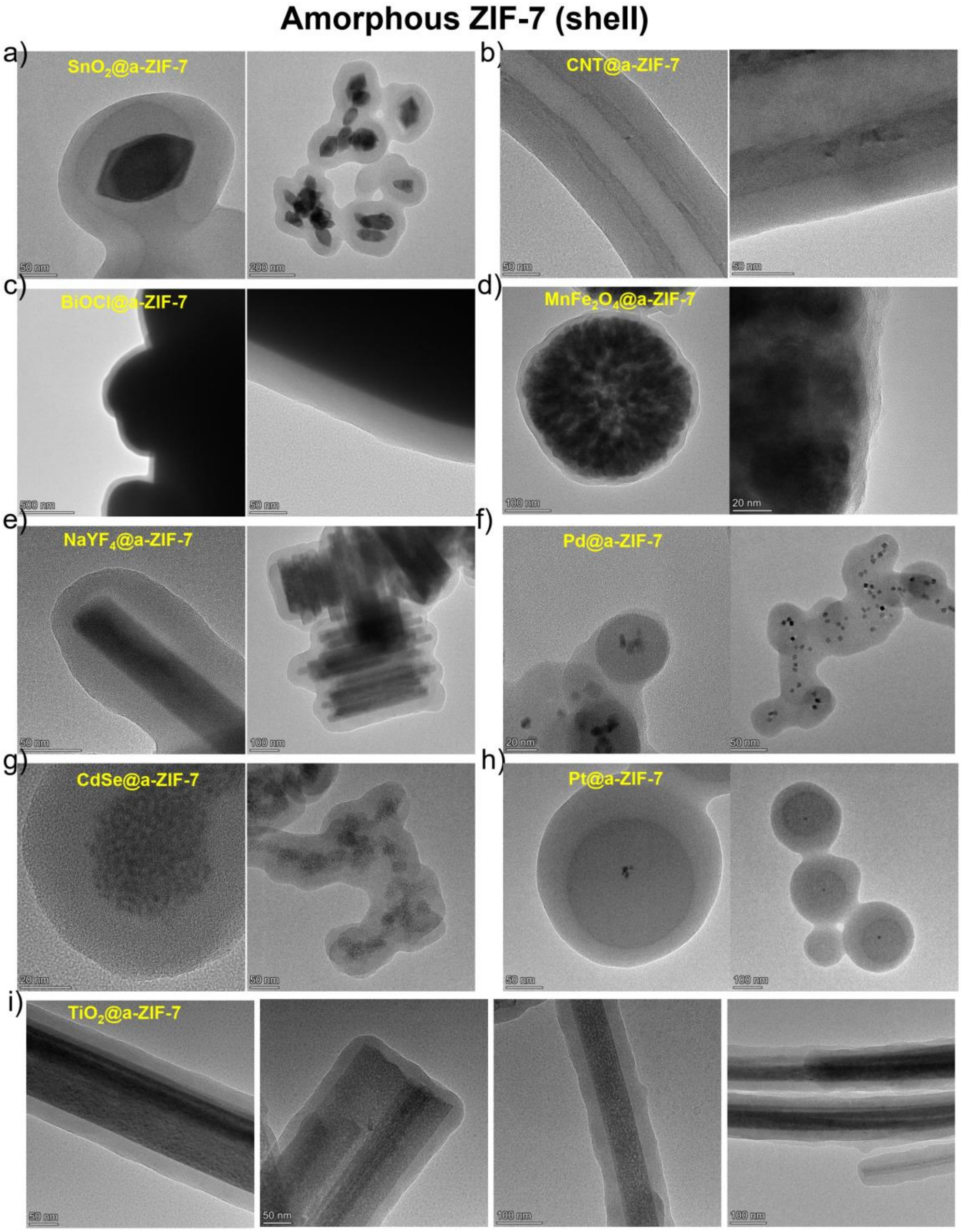

Supplementary Figure 28. (a) TEM images of SnO_2@a-ZIF-7. (b) TEM images of CNT@a-ZIF-7. (c) TEM images of BiOCl @a-ZIF-7. (d) TEM images of $MnFe_2O_4$@a-ZIF-7. (e) TEM images of $NaYF_4$@a-ZIF-7. (f) TEM images of Pd@a-ZIF-7. (g) TEM images of CdSe@a-ZIF-7. (h) TEM images of Pt@a-ZIF-7. (i) TEM images of TiO_2@a-ZIF-7.

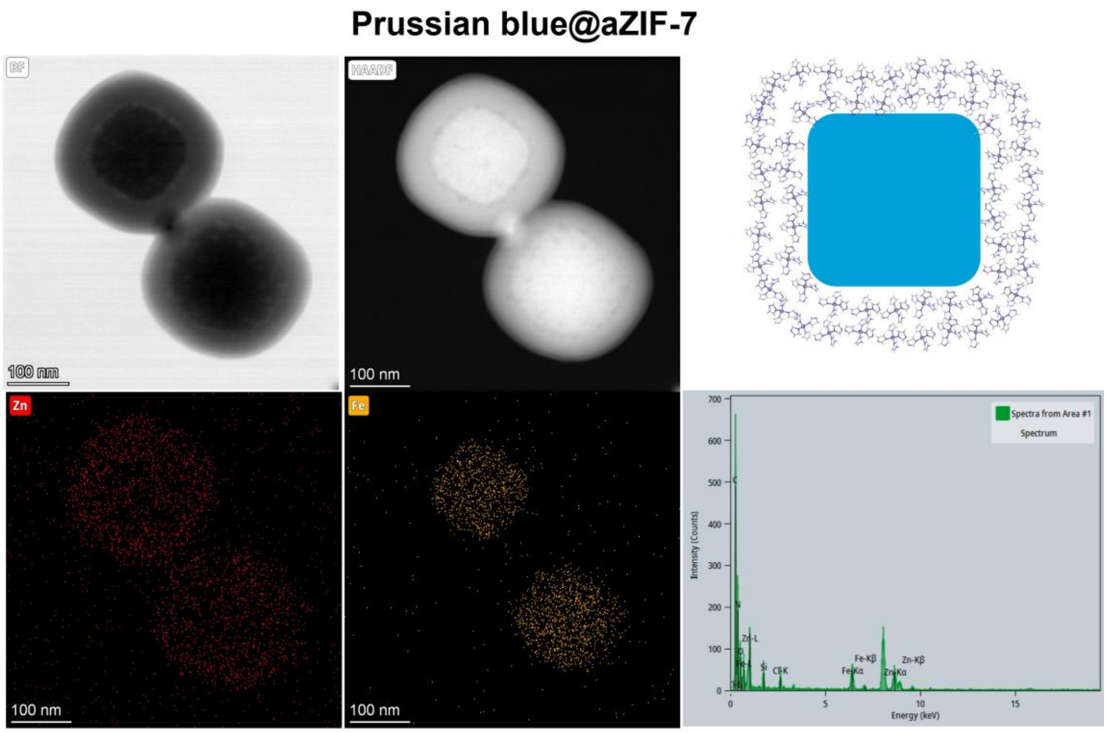

Supplementary Figure 29. TEM image, element mapping images and representative energy dispersive X-ray (EDX) spectrum of PB@a-ZIF-7 colloids. Fe is primarily localized within the interior, whereas Zn is distributed in the external shell. Fe originates from Prussian blue, while Zn is derived from amorphous ZIF-7. This indicates the successful growth of amorphous ZIF-7 on PB.

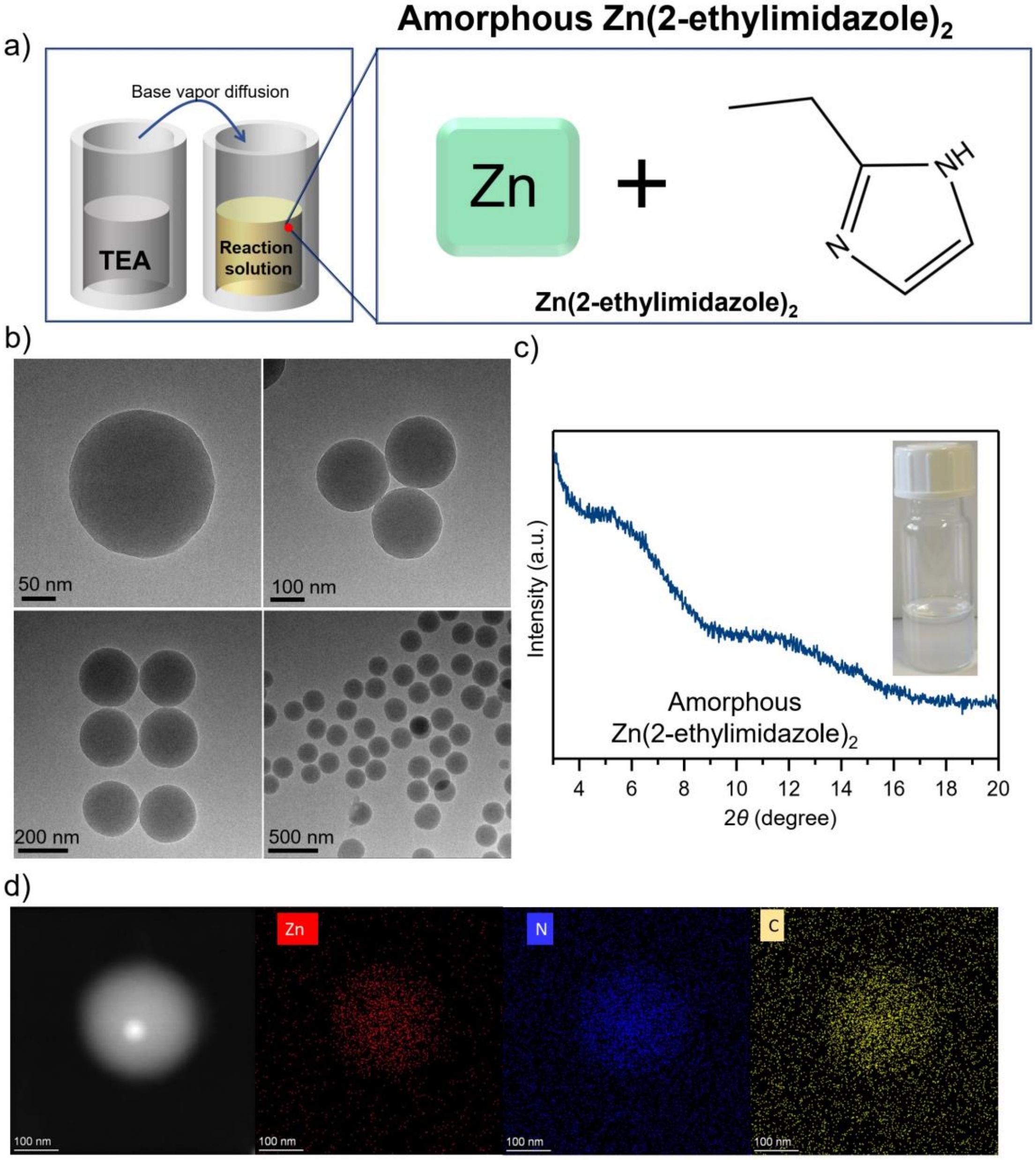

Supplementary Figure 30. (a) Schematic illustration of the preparation of amorphous Zn(2-ethylimidazole)$_2$ spheres using the TEA diffusion method. (b) TEM images of a-Zn(2-ethylimidazole)$_2$ spheres. (c) PXRD pattern of a-Zn(2-ethylimidazole)$_2$ spheres, with an inset showing an optical image of a- Zn(2-ethylimidazole)$_2$ colloidal solution. (d) TEM images and element mapping images of a-Zn(2-ethylimidazole)$_2$ spheres. Zn, N, and C elements are uniformly distributed.

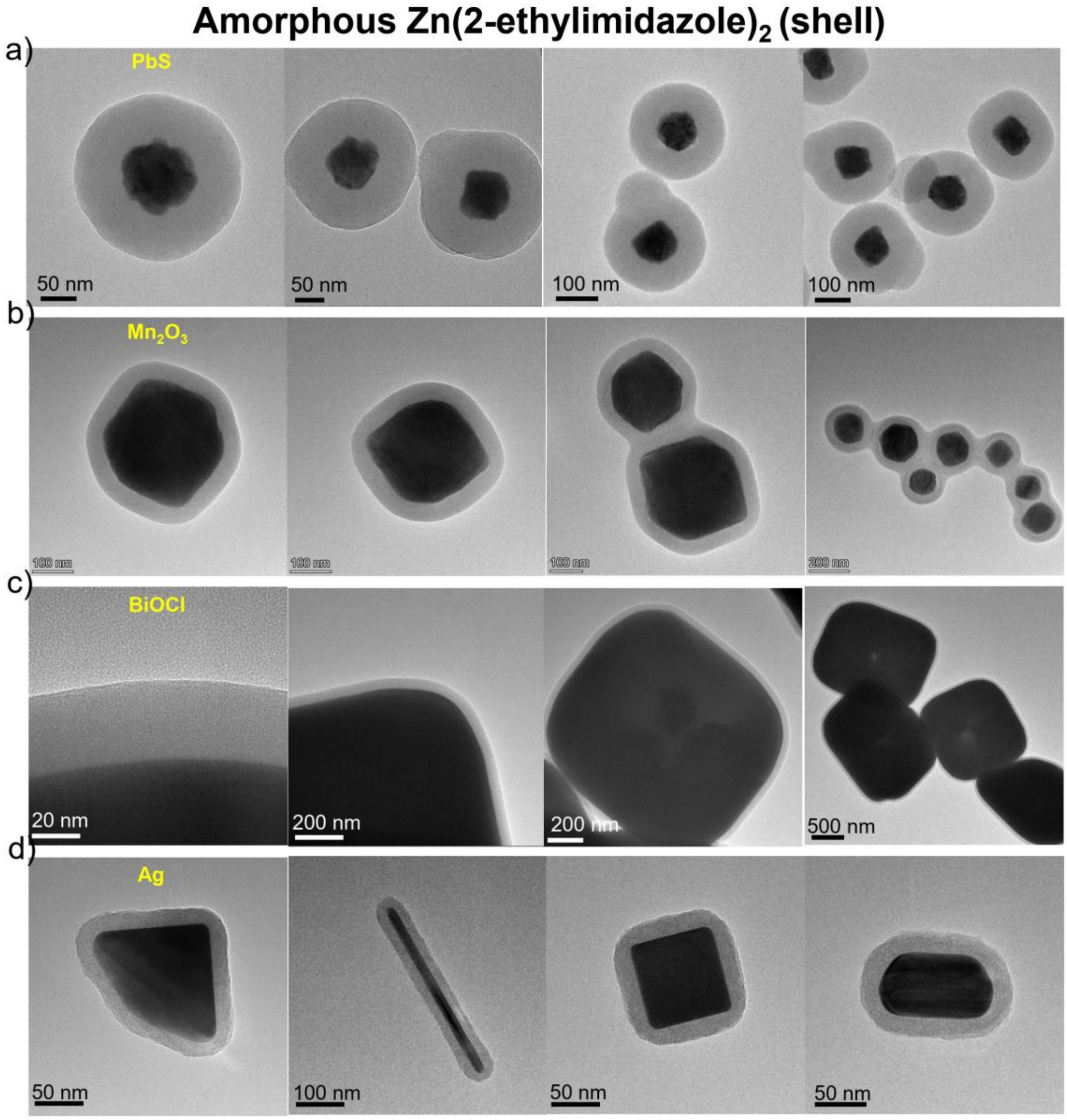

Supplementary Figure 31. (a) TEM images of PbS@a-Zn(2-ethylimidazole)$_2$. (b) TEM images of Mn_2O_3@a-Zn(2-ethylimidazole)$_2$. (c) TEM images of BiOCl@a-Zn(2-ethylimidazole)$_2$. (d) TEM images of Ag@a-Zn(2-ethylimidazole)$_2$ with different morphologies.

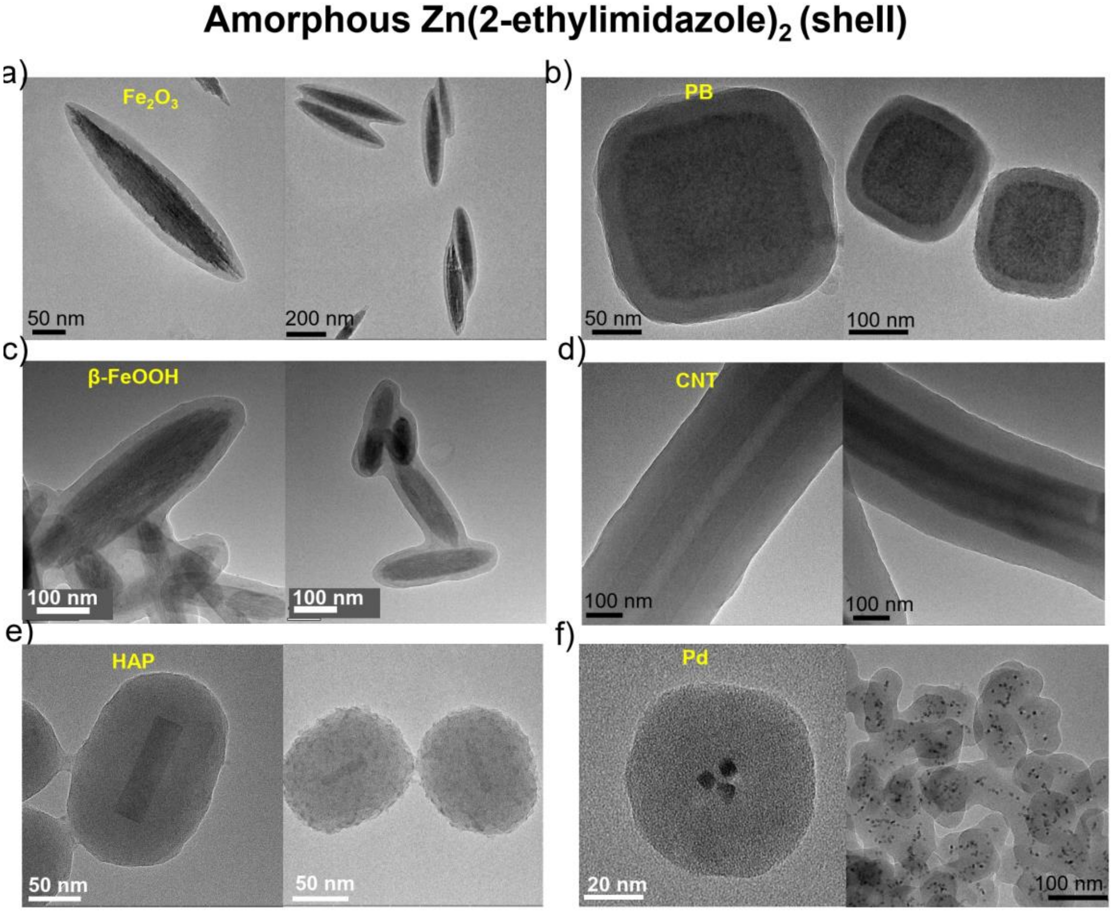

Supplementary Figure 32. (a) TEM images of Fe_2O_3@a-Zn(2-ethylimidazole)$_2$. (b) TEM images of PB@a-Zn(2-ethylimidazole)$_2$. (c) TEM images of β-FeOOH@a-Zn(2-ethylimidazole)$_2$. (d) TEM images of CNT@a-Zn(2-ethylimidazole)$_2$. (e) TEM images of HAP@a-Zn(2-ethylimidazole)$_2$. Hydroxyapatite (HAP) is $Ca_5(PO_4)_3OH$. (f) TEM images of Pd@a-Zn(2-ethylimidazole)$_2$.

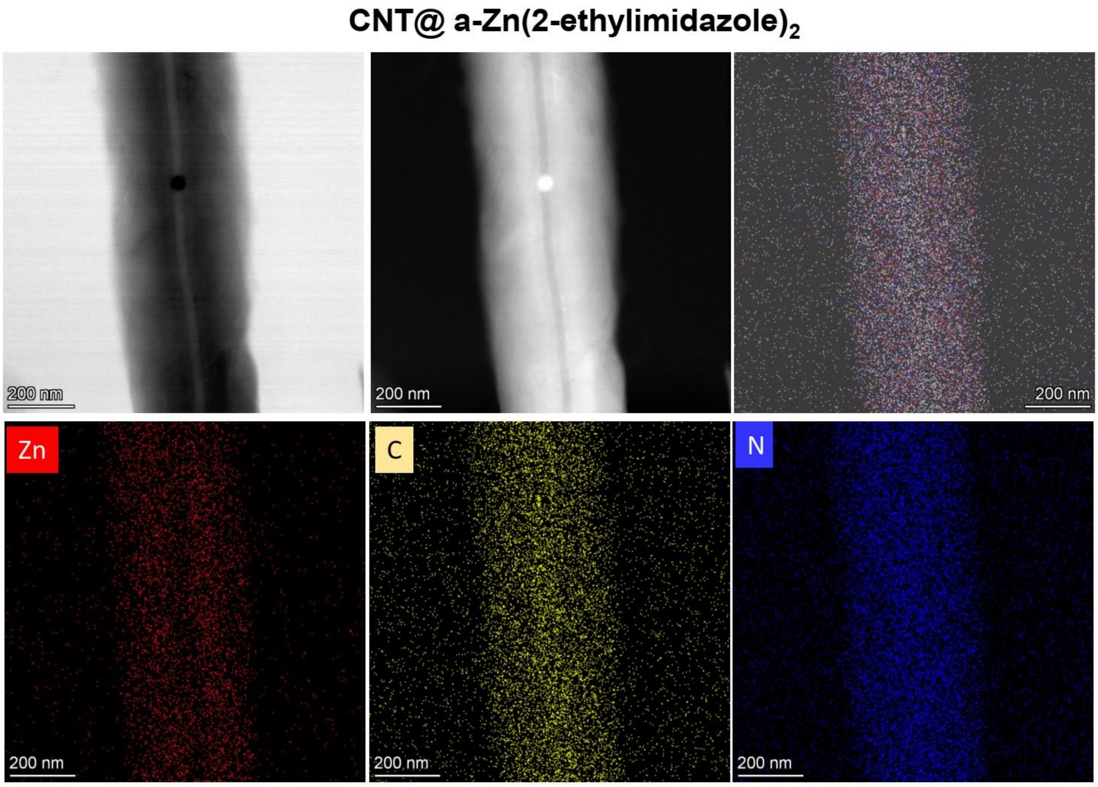

Supplementary Figure 33. TEM image and element mapping images of CNT@a-Zn(2-ethylimidazole)$_2$ colloids.

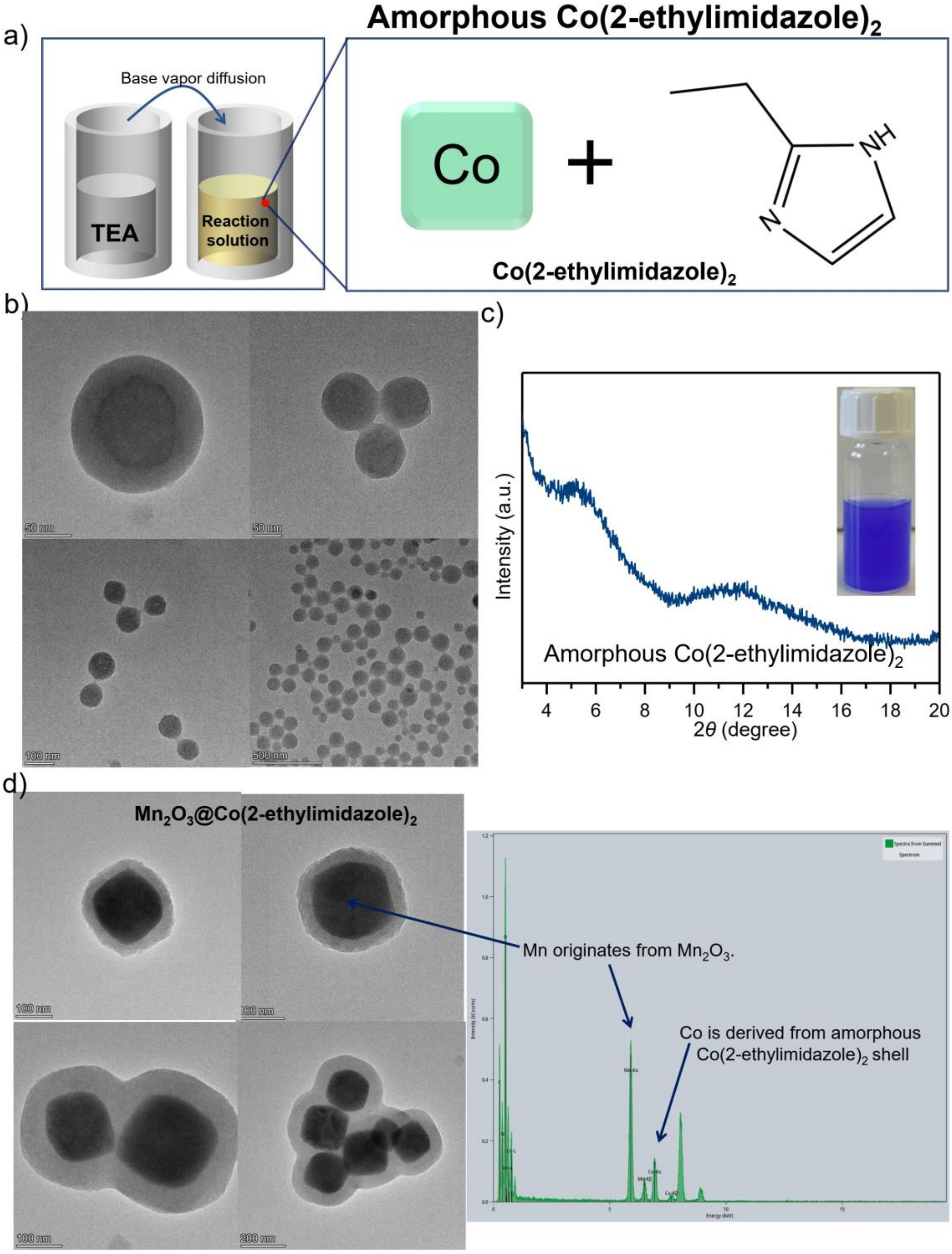

Supplementary Figure 34. (a) Schematic illustration of the preparation of amorphous Co(2-ethylimidazole)$_2$ spheres using the TEA diffusion method. (b) TEM images of a-Co(2-ethylimidazole)$_2$ spheres. (c) PXRD pattern of a-Co(2-ethylimidazole)$_2$ spheres, with an inset showing an optical image of a-Co(2-ethylimidazole)$_2$ colloidal solution. (d) TEM images and representative energy dispersive X-ray (EDX) spectrum of Mn_2O_3@a-Co(2-ethylimidazole)$_2$ core-shell structures.

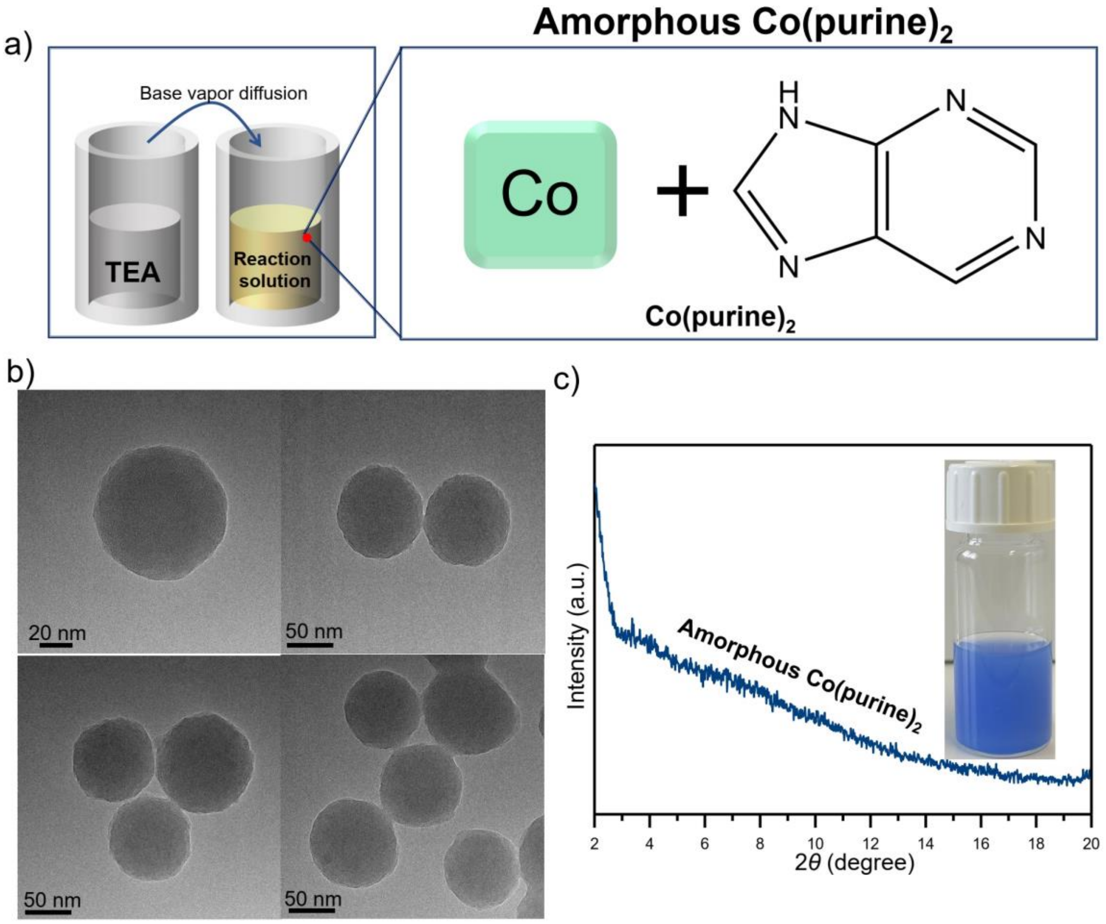

Supplementary Figure 35. (a) Schematic illustration of the preparation of amorphous Co(purine)$_2$ spheres using the TEA diffusion method. (b)TEM images of a-Co(purine)$_2$ spheres. (c) PXRD pattern of a-Co(purine)$_2$ spheres, with an inset showing an optical image of a-Co(purine)$_2$ colloidal solution.

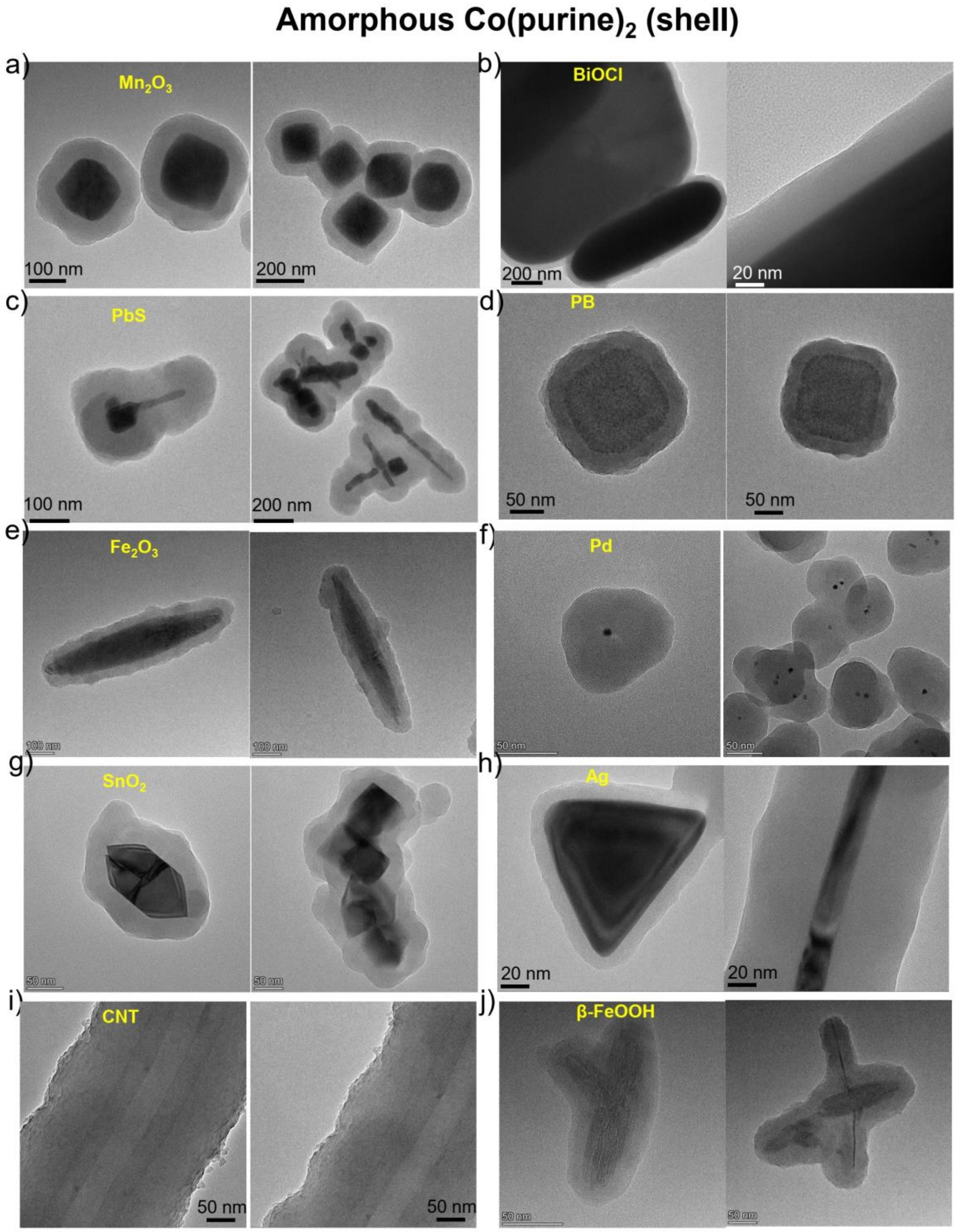

Supplementary Figure 36. (a) TEM images of Mn_2O_3@a-Co(purine)$_2$. (b) TEM images of BiOCl@a-Co(purine)$_2$. (c) TEM images of PbS@a-Co(purine)$_2$. (d) TEM images of PB@a-Co(purine)$_2$. (e) TEM images of Fe_2O_3@a-Co(purine)$_2$. (f) TEM images of Pd@a-Co(purine)$_2$. (g) TEM images of SnO_2@a-Co(purine)$_2$. (h) TEM images of Ag@a-Co(purine)$_2$. (i) TEM images of CNT@a-Co(purine)$_2$. (i) TEM images of β-FeOOH@a-Co(purine)$_2$.

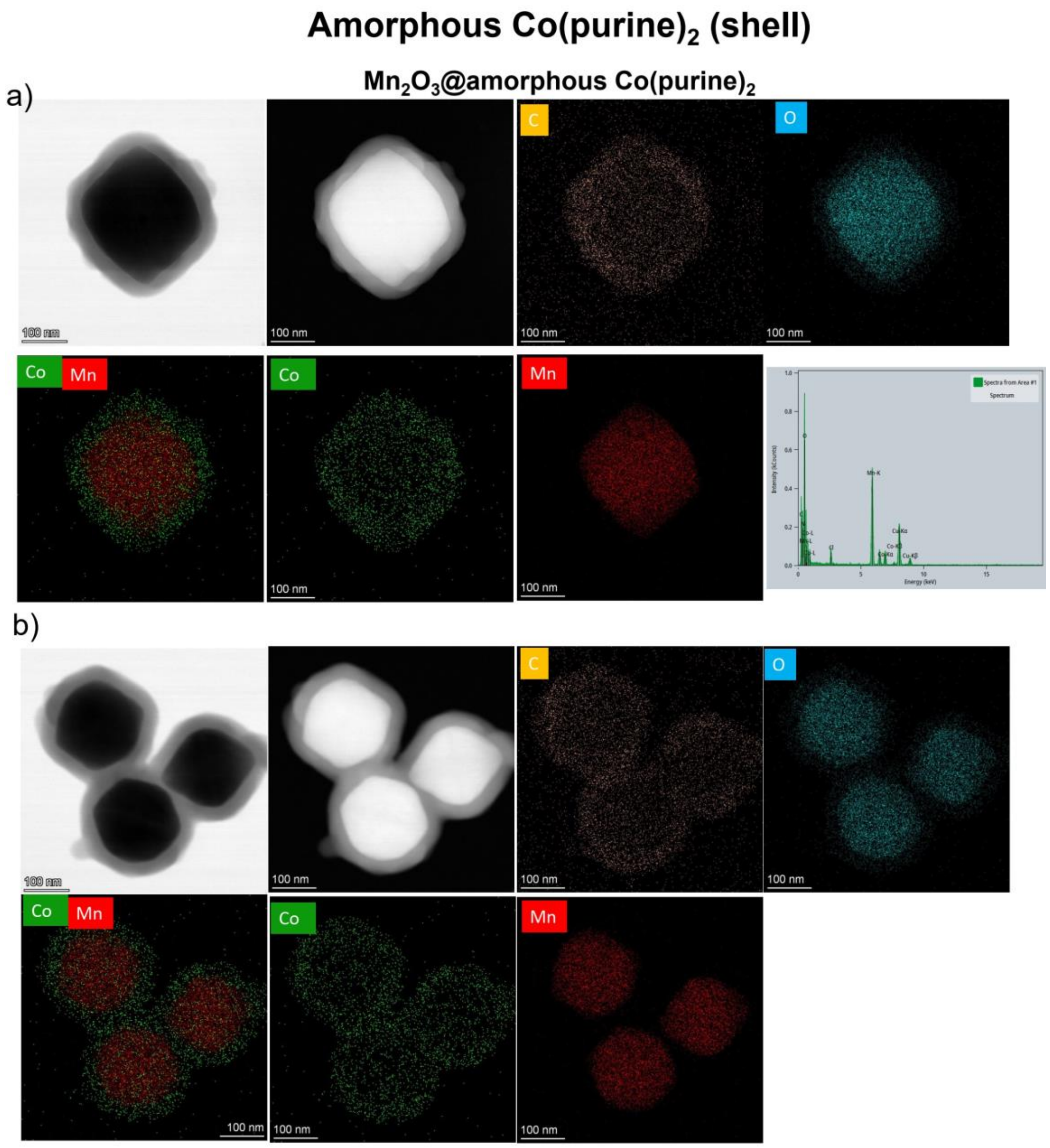

Supplementary Figure 37. (a-b) Bright field-and HAADF-STEM images, element mapping images and representative energy dispersive X-ray (EDX) spectrum of Mn_2O_3@a-Co(purine)$_2$ colloids.

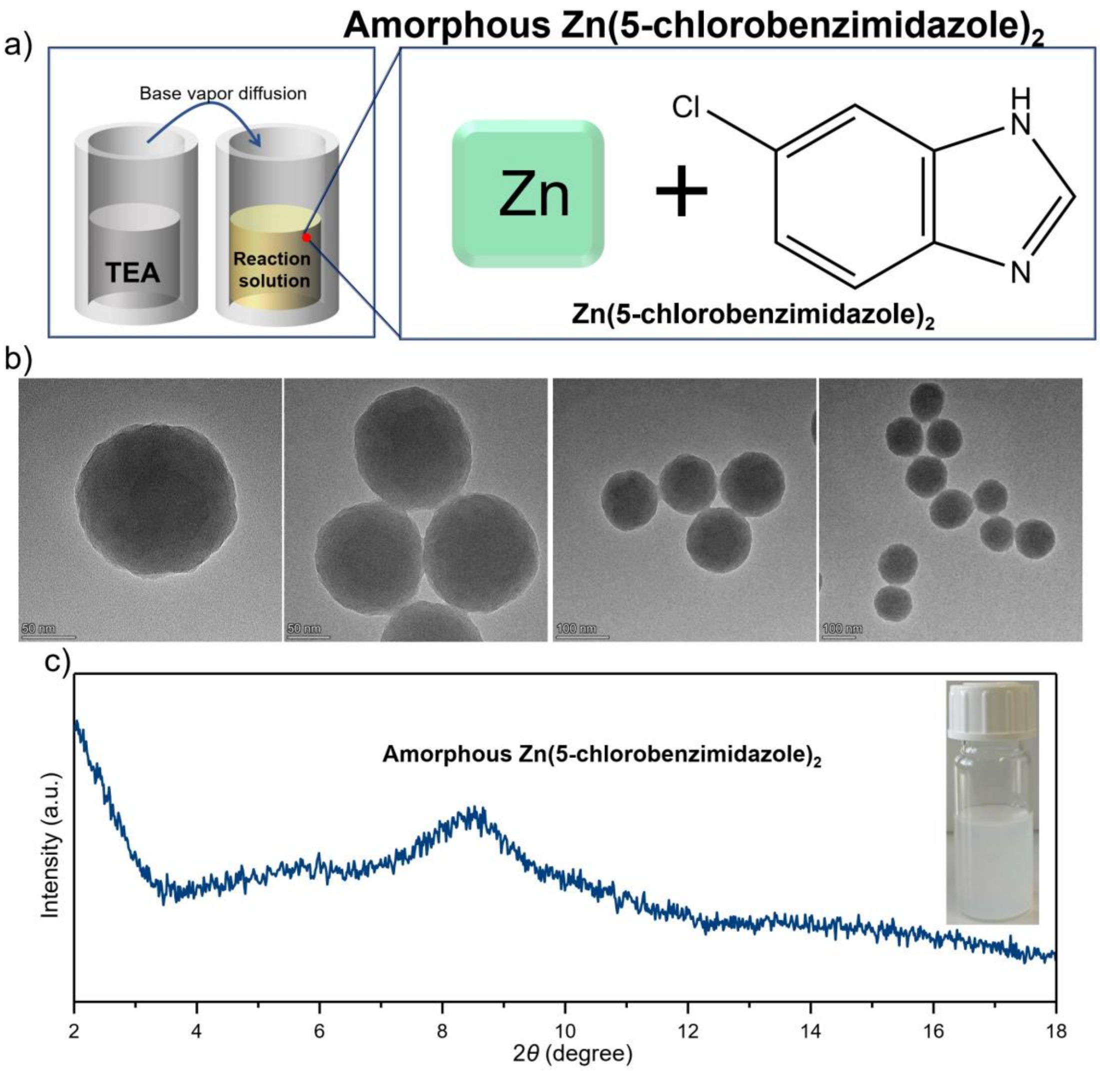

Supplementary Figure 38. (a) Schematic illustration of the preparation of amorphous Zn(5-chlorobenzimidazole)$_2$ spheres using the TEA diffusion method. (b) TEM images of a-Zn(5-chlorobenzimidazole)$_2$ spheres. (c) PXRD pattern of a-Zn(5-chlorobenzimidazole)$_2$ spheres, with an inset showing an optical image of a-Zn(5-chlorobenzimidazole)$_2$ colloidal solution.

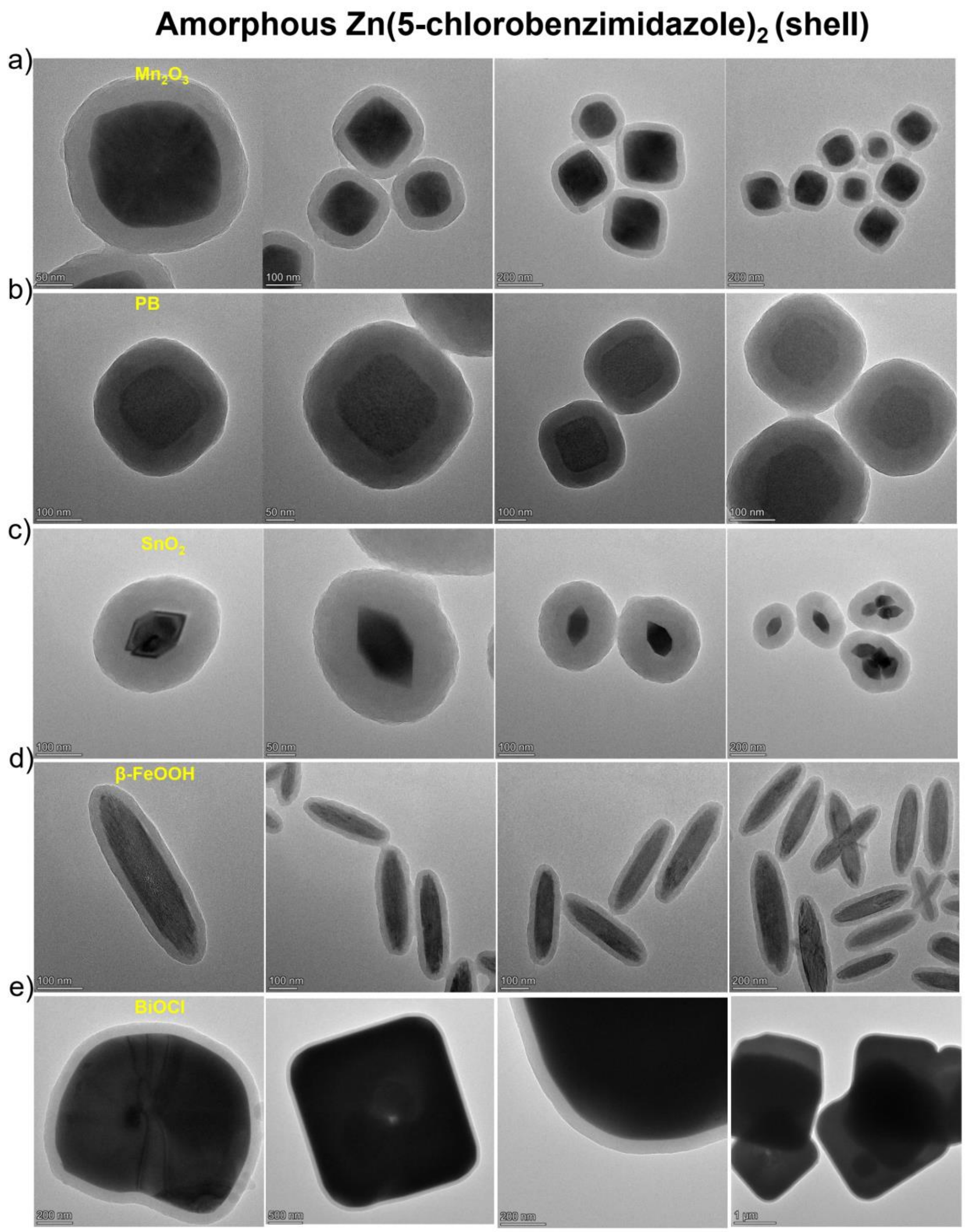

Supplementary Figure 39. (a) TEM images of Mn_2O_3@a-Zn(5-chlorobenzimidazole)$_2$. (b) TEM images of PB@a-Zn(5-chlorobenzimidazole)$_2$. (c) TEM images of SnO_2@a-Zn(5-chlorobenzimidazole)$_2$. (d) TEM images of β-FeOOH@a-Zn(5-chlorobenzimidazole)$_2$. (e) TEM images of BiOCl@a-Zn(5-chlorobenzimidazole)$_2$.

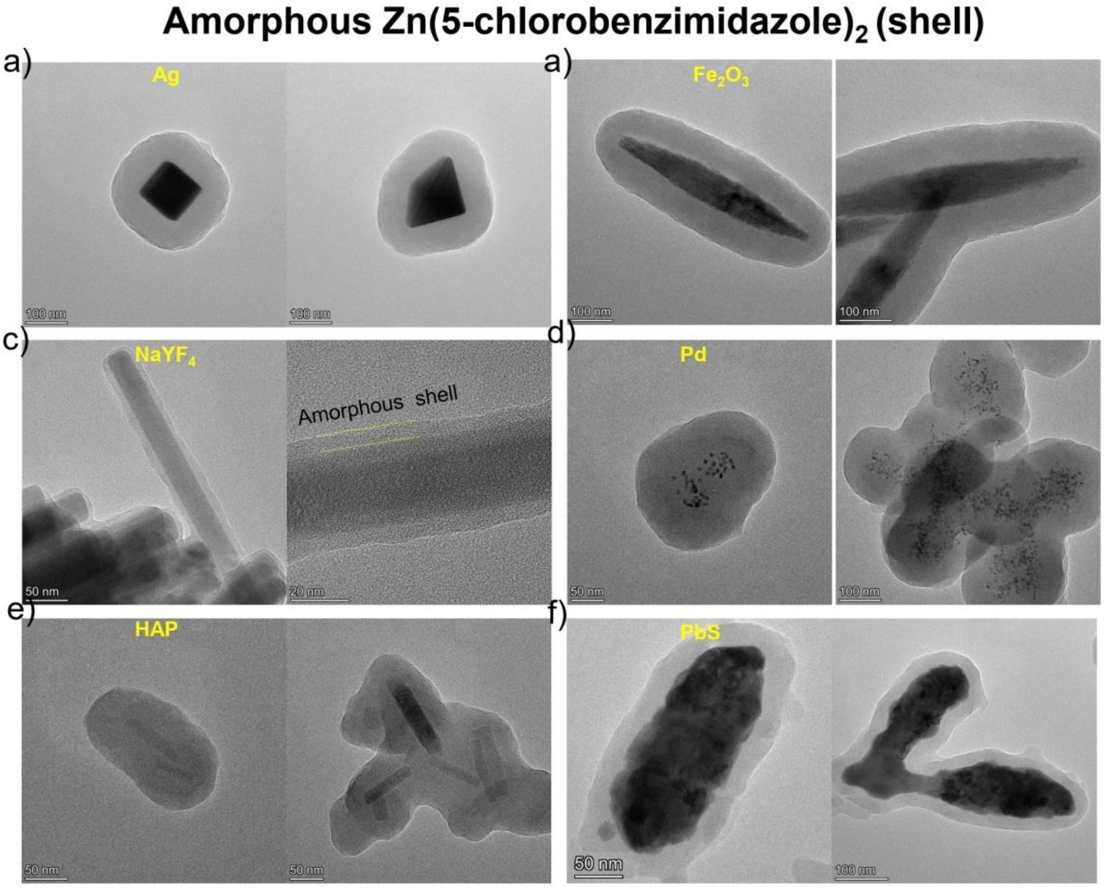

Supplementary Figure 40. (a) TEM images of Ag@a-Zn(5-chlorobenzimidazole)$_2$. (b) TEM images of Fe_2O_3@a-Zn(5-chlorobenzimidazole)$_2$. (c) TEM images of $NaYF_4$@a-Zn(5-chlorobenzimidazole)$_2$. (d) TEM images of Pd@a-Zn(5-chlorobenzimidazole)$_2$. (e) TEM images of HAP@a-Zn(5-chlorobenzimidazole)$_2$. (f) TEM images of PbS@a-Zn(5-chlorobenzimidazole)$_2$.

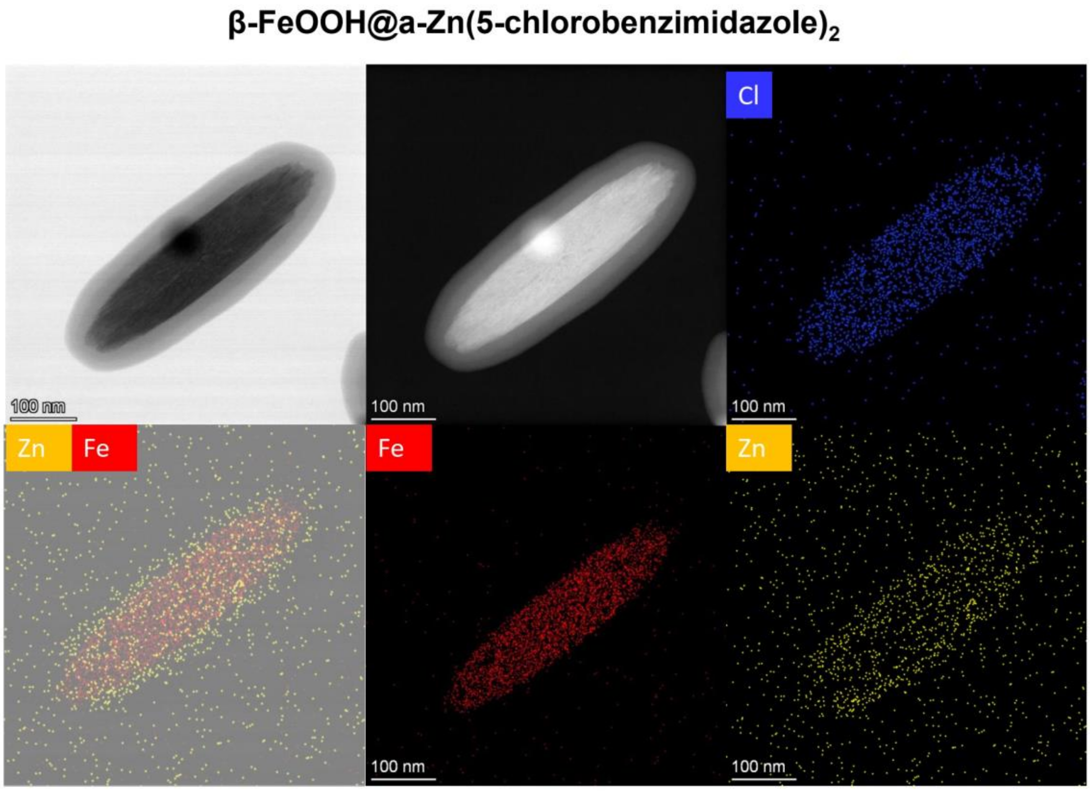

Supplementary Figure 41. Bright filed- and HAADF-STEM images and element mapping images of β-FeOOH@a-Zn(5-chlorobenzimidazole)$_2$ colloids.

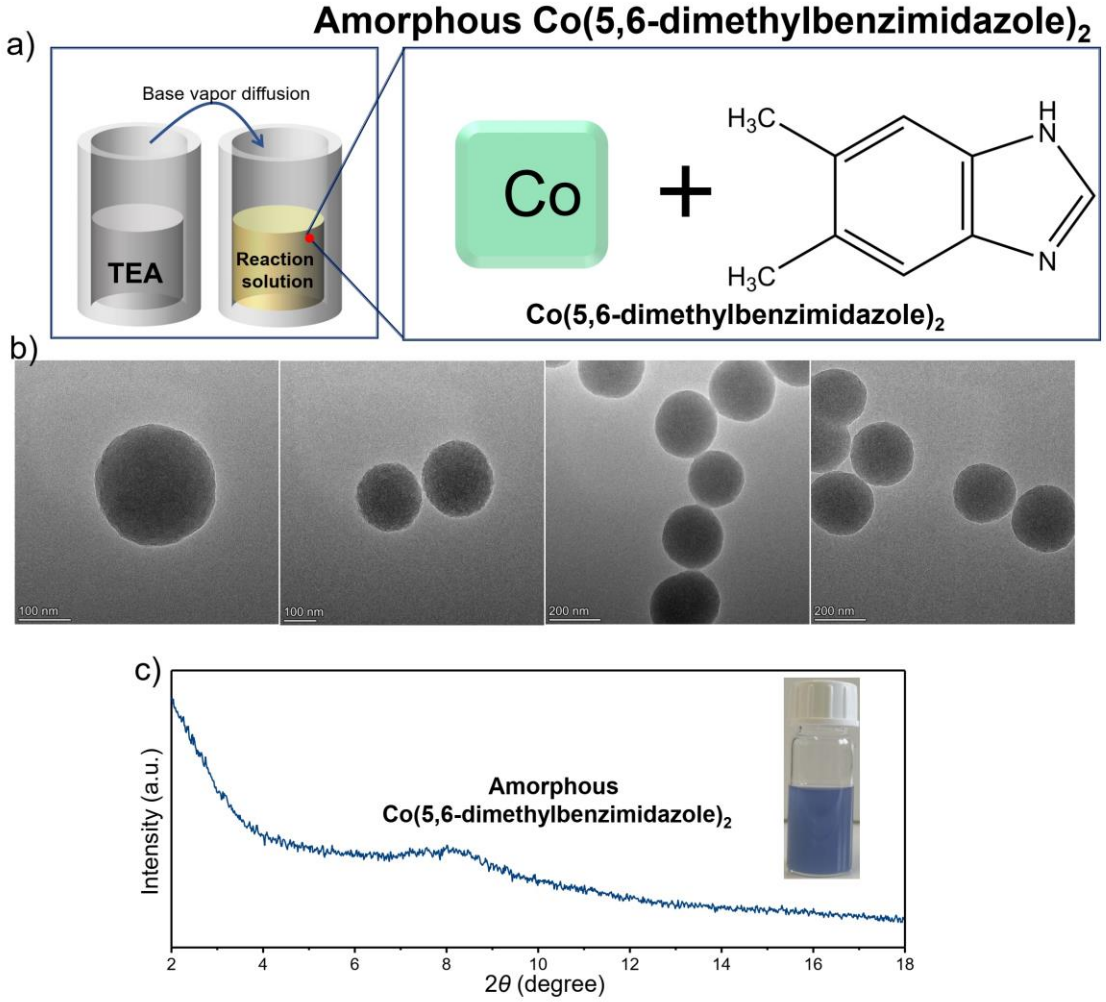

Supplementary Figure 42. (a) Schematic illustration of the preparation of amorphous Co(5,6-dimethylbenzimidazole)$_2$ spheres using the TEA diffusion method. (b) TEM images of a-Co(5,6-dimethylbenzimidazole)$_2$ spheres. (c) PXRD pattern of a-Co(5,6-dimethylbenzimidazole)$_2$ spheres, with an inset showing an optical image of a-Co(5,6-dimethylbenzimidazole)$_2$ colloidal solution.

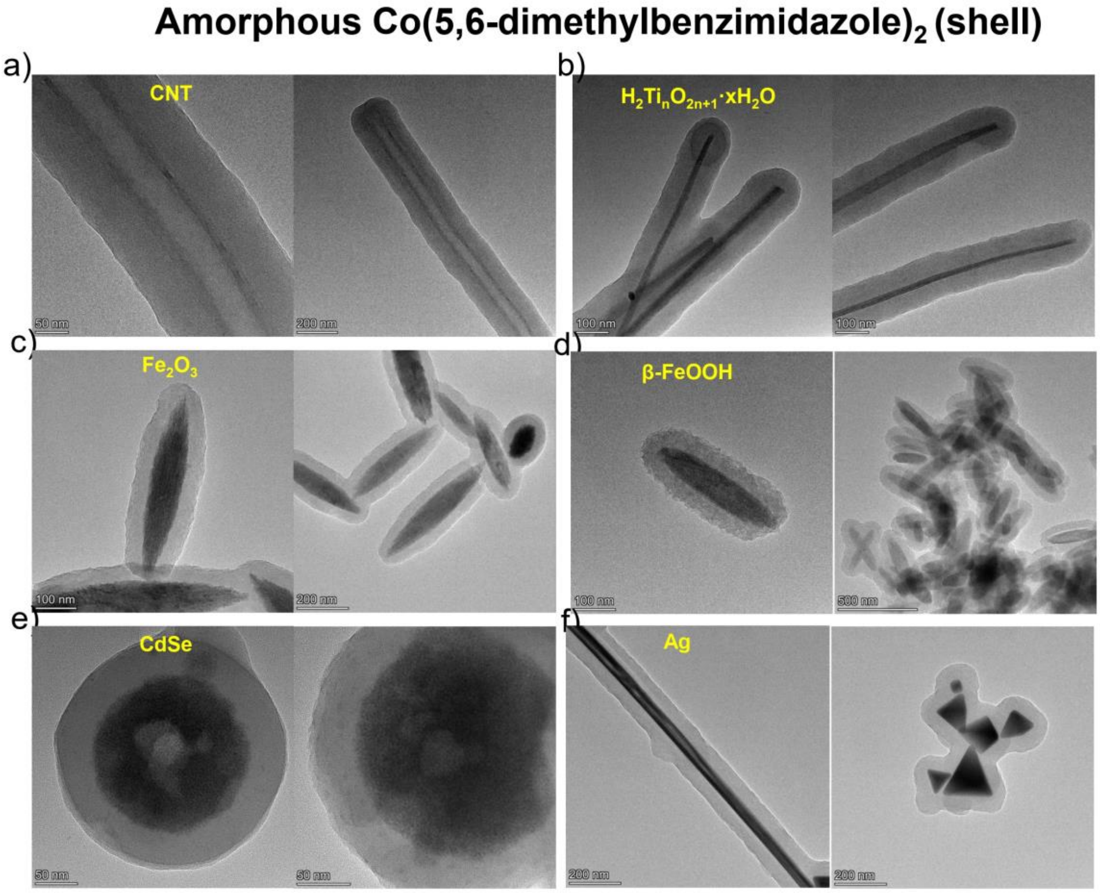

Supplementary Figure 43. (a) TEM images of CNT@a-Co(5,6-dimethylbenzimidazole)$_2$. (b) TEM images of $H_2Ti_nO_{2n+1}\cdot xH_2O$@a-Co(5,6-dimethylbenzimidazole)$_2$. (c) TEM images of Fe_2O_3@a-Co(5,6-dimethylbenzimidazole)$_2$. (d) TEM images of β-FeOOH @a-Co(5,6-dimethylbenzimidazole)$_2$. (e) TEM images of CdSe@a-Co(5,6-dimethylbenzimidazole)$_2$. (f) TEM images of Ag@a-Co(5,6-dimethylbenzimidazole)$_2$.

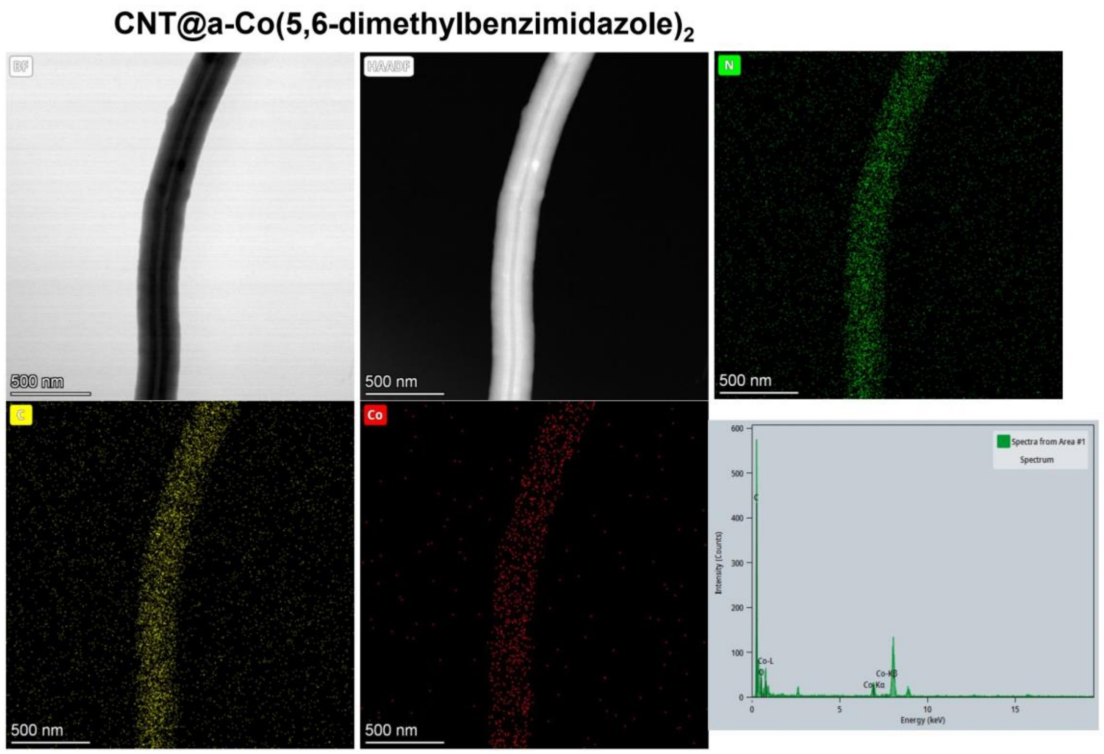

Supplementary Figure 44. TEM images, element mapping images and representative energy dispersive X-ray (EDX) spectrum of CNT@a-Co(5,6-dimethylbenzimidazole)$_2$.

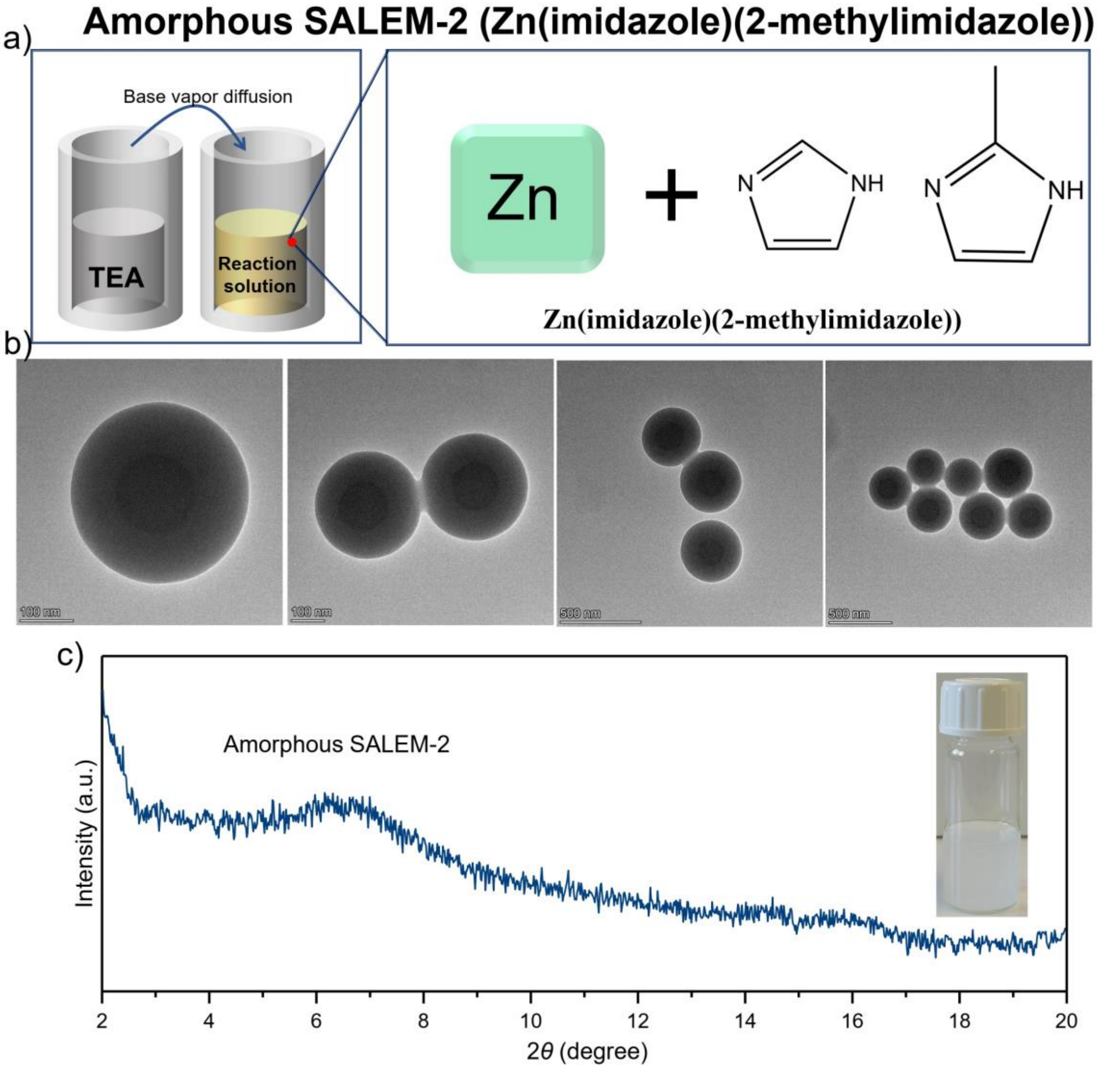

Supplementary Figure 45. (a) Schematic illustration of the preparation of amorphous SALEM-2 spheres using the TEA diffusion method. (b) TEM images of a-SALEM-2 spheres. (c) PXRD pattern of a-SALEM-2 spheres, with an inset showing an optical image of a-SALEM-2 colloidal solution.

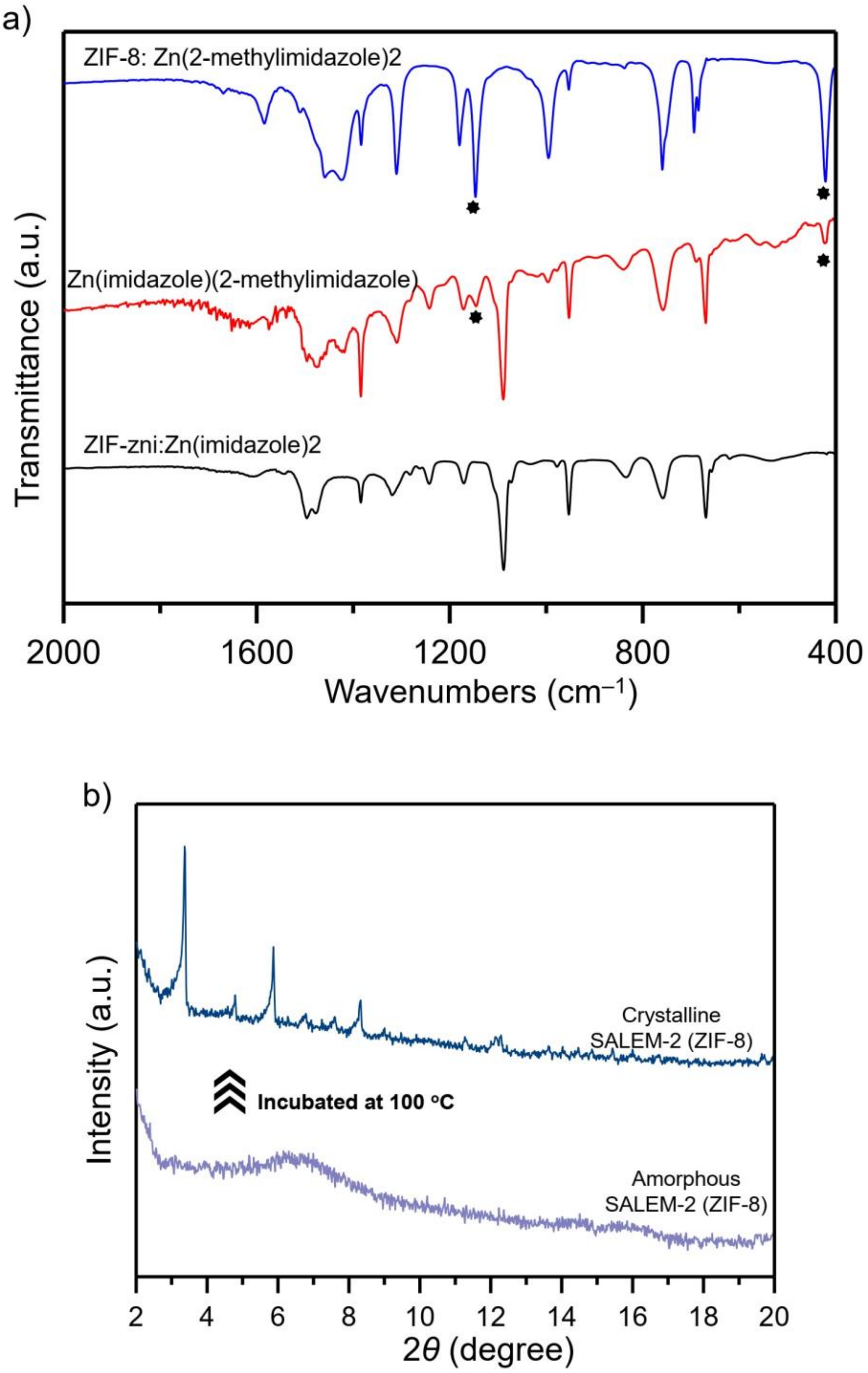

Supplementary Figure 46. (a) FT-IR spectra of crystalline ZIF-zni, amorphous SALEM-2 spheres and crystalline ZIF-8. The results indicate that the SALEM-2 contains both imidazole and 2-methylimidazole. (b) PXRD patterns of amorphous SALEM-2 spheres incubated at 110 ℃. Elevated temperatures trigger the transition of SALEM-2 from an amorphous to a crystalline state, indicating the influence of thermodynamic control.

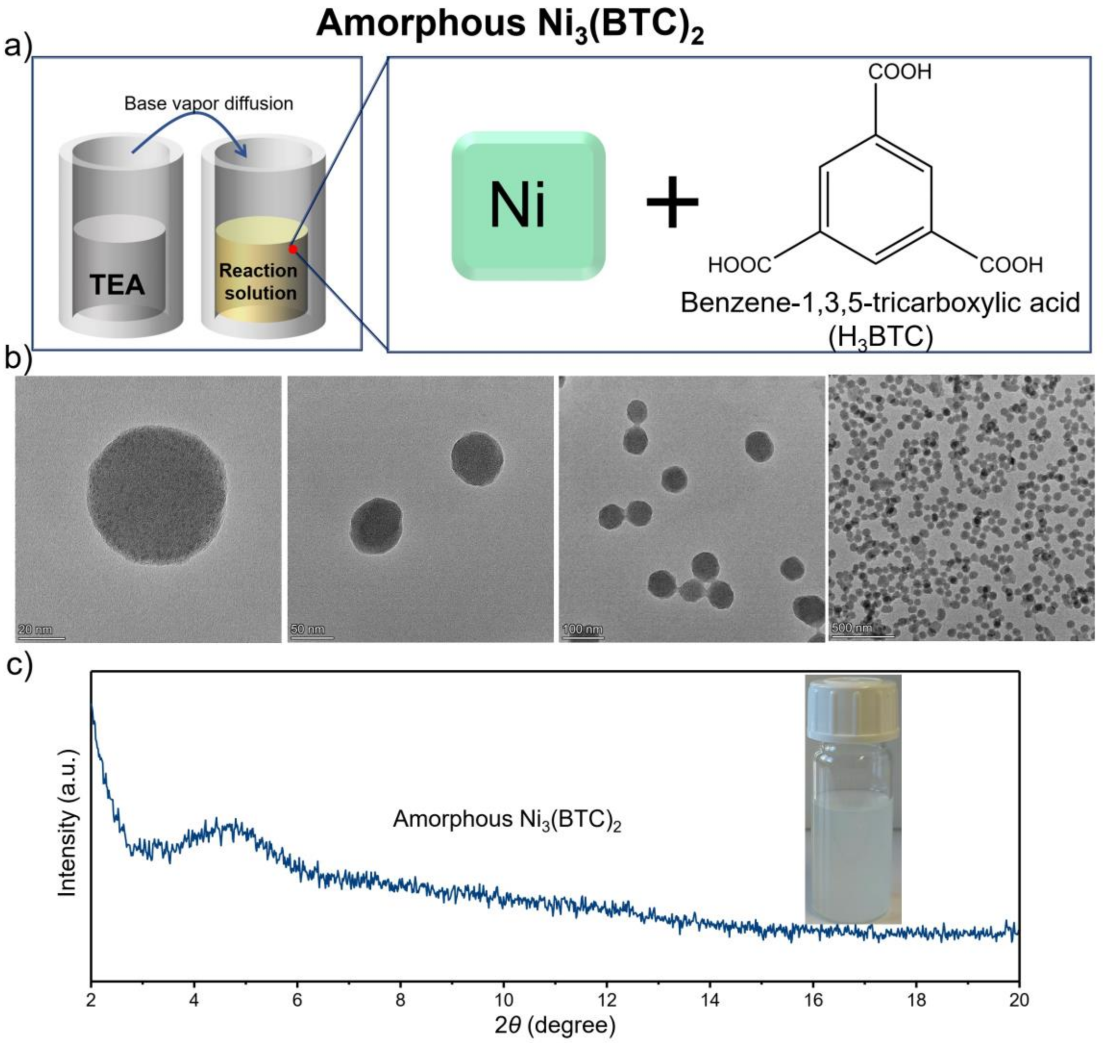

Supplementary Figure 47. (a) Schematic illustration of the preparation of amorphous $Ni_3(BTC)_2$ spheres using the TEA diffusion method. (b) TEM images of a-$Ni_3(BTC)_2$ spheres. (c) PXRD pattern of a-$Ni_3(BTC)_2$ spheres, with an inset showing an optical image of a-$Ni_3(BTC)_2$ colloidal solution.

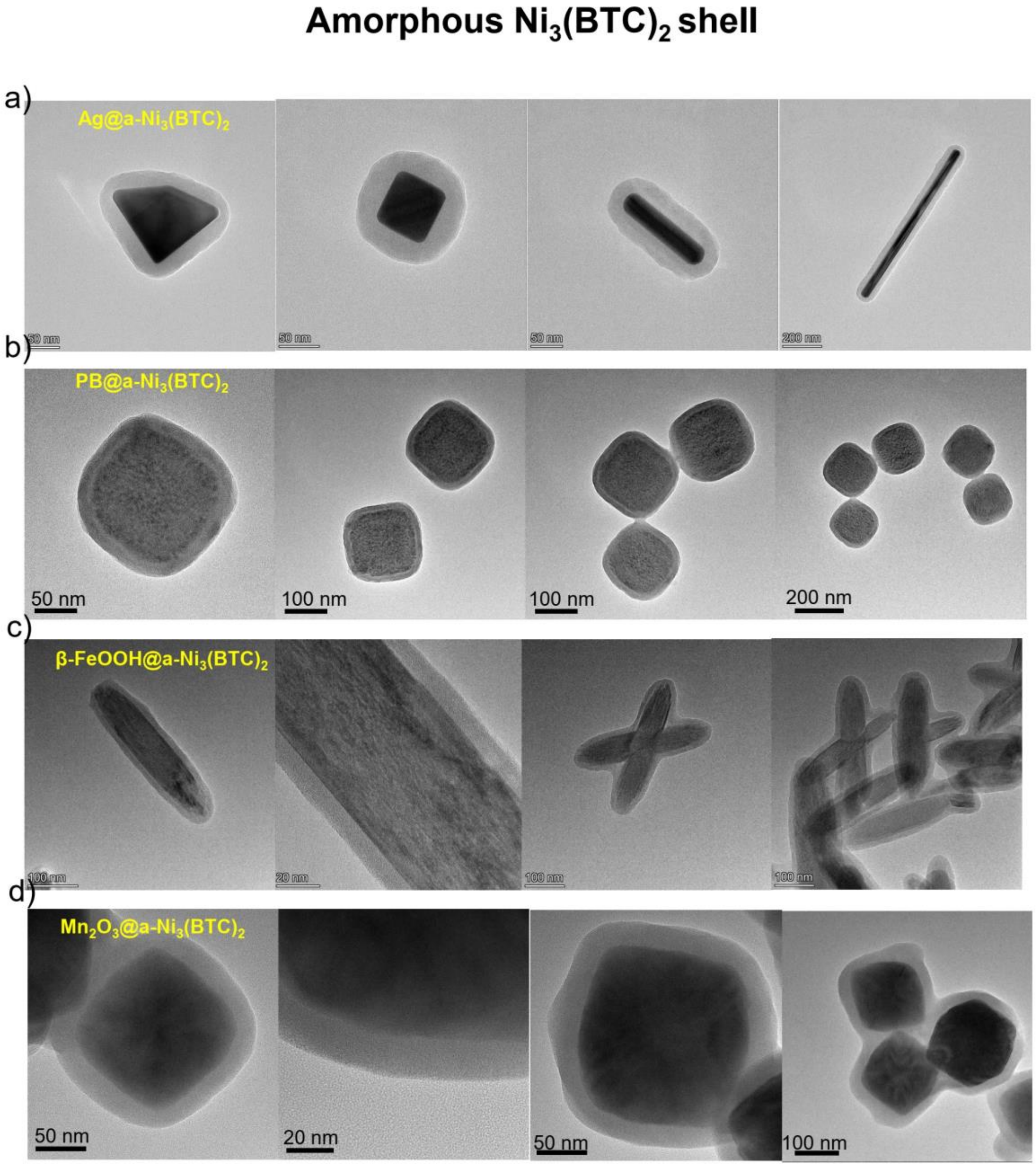

Supplementary Figure 48. (a) TEM images of Ag@a-$Ni_3(BTC)_2$ with different morphologies. (b) TEM images of PB@a-$Ni_3(BTC)_2$. (c) TEM images of β-FeOOH@a-$Ni_3(BTC)_2$. (d) TEM images of Mn_2O_3@a-$Ni_3(BTC)_2$.

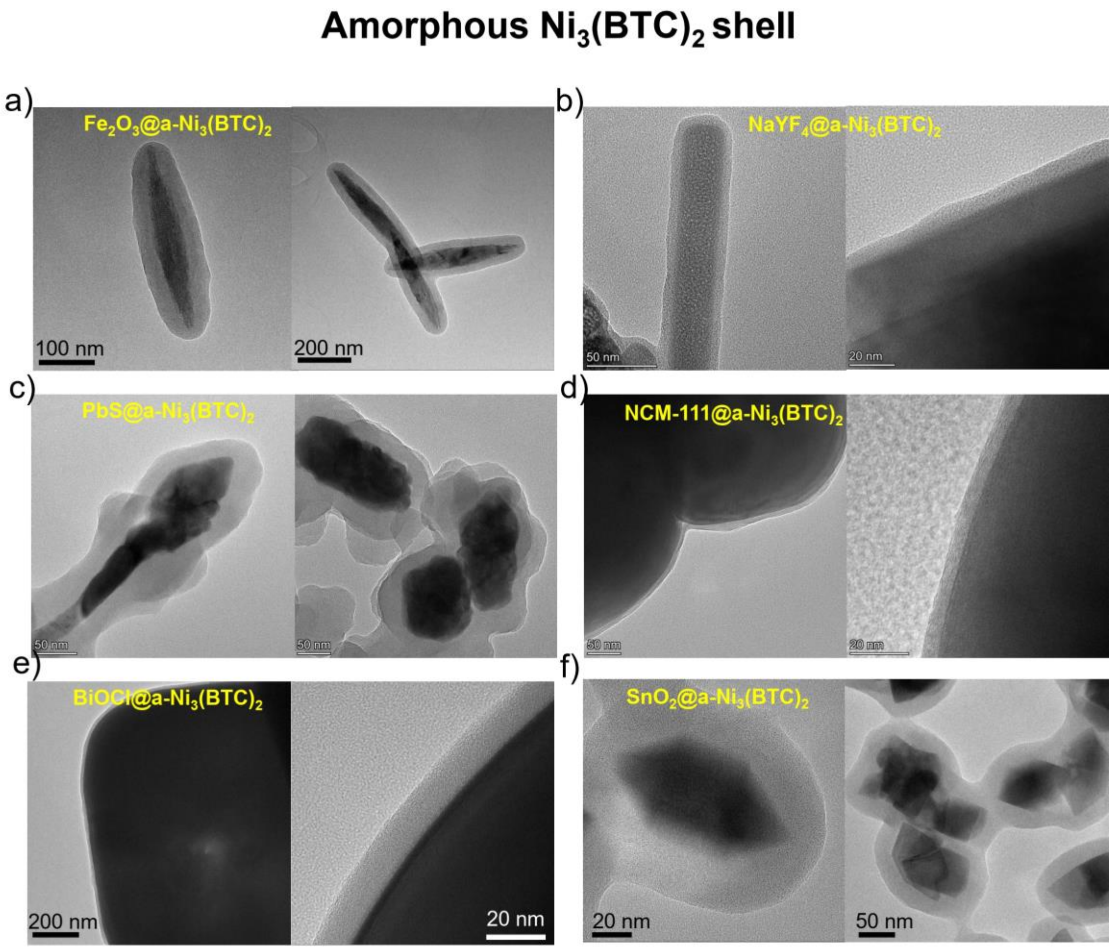

Supplementary Figure 49. (a) TEM images of Fe_2O_3@a-$Ni_3(BTC)_2$. (b) TEM images of $NaYF_4$@a-$Ni_3(BTC)_2$. (c) TEM images of PbS@a-$Ni_3(BTC)_2$. (d) TEM images of NCM-111@a-$Ni_3(BTC)_2$. NCM-111 is lithium nickel manganese cobalt oxide. (e) TEM images of BiOCl@a-$Ni_3(BTC)_2$. (f) TEM images of SnO_2@a-$Ni_3(BTC)_2$.

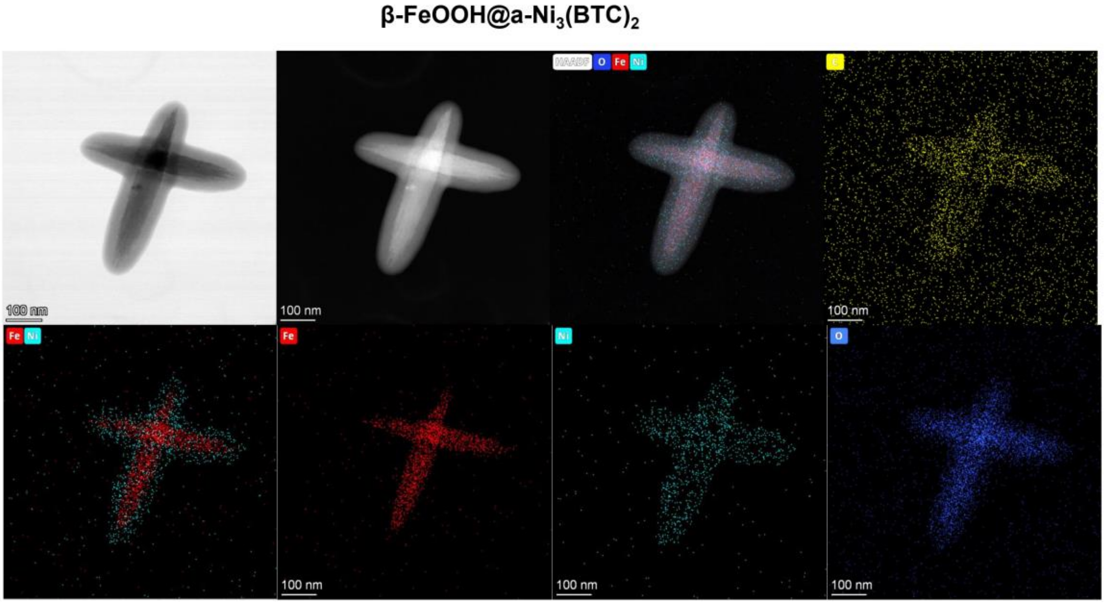

Supplementary Figure 50. Bright Field- and HAADF-STEM images and element mapping images of β-FeOOH@a-$Ni_3(BTC)_2$.

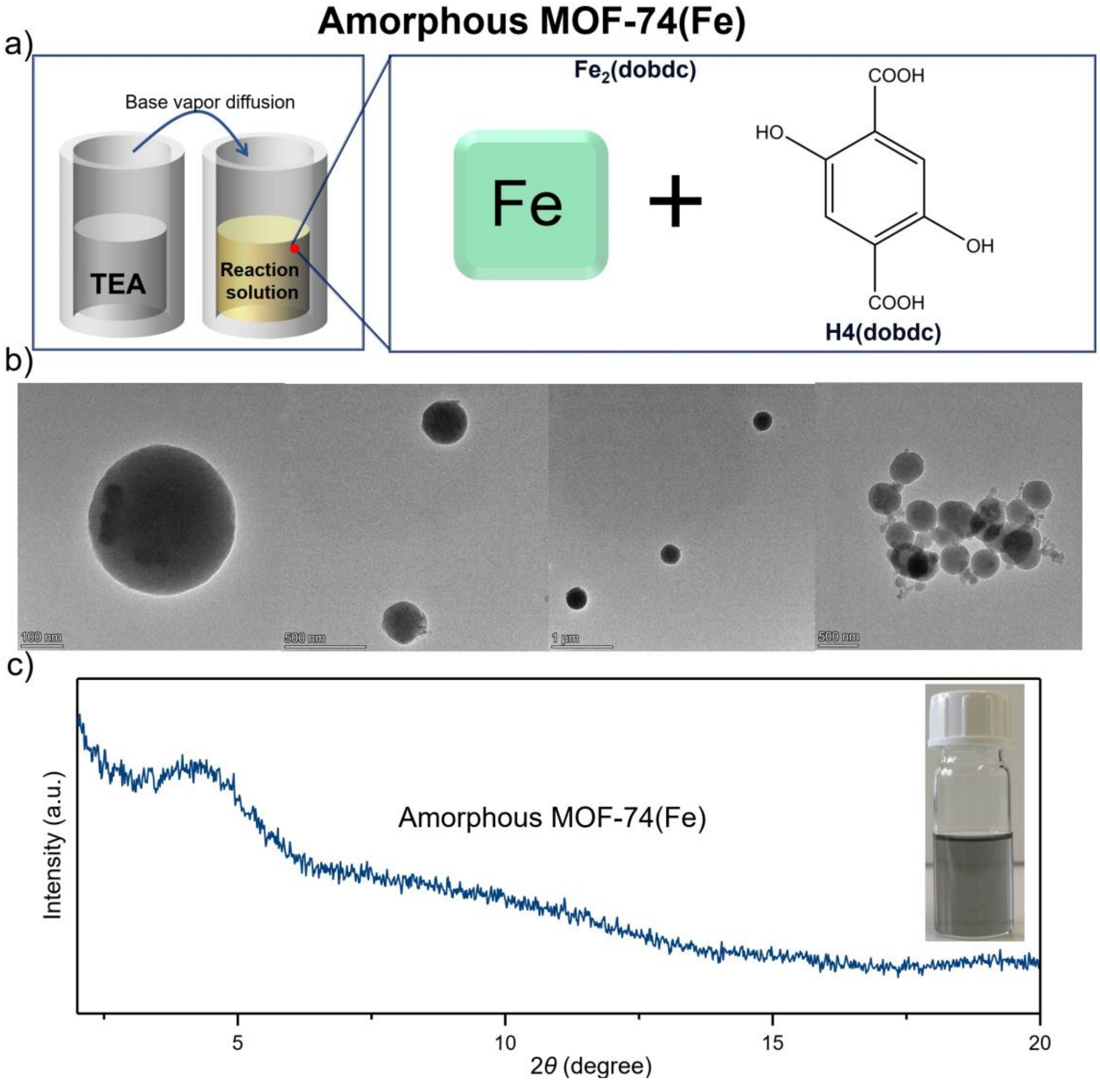

Supplementary Figure 51. (a) Schematic illustration of the preparation of amorphous MOF-74(Fe) using the TEA diffusion method. (b) TEM images of a-MOF-74(Fe) spheres. (c) PXRD pattern of a-MOF-74(Fe), with an inset showing an optical image of a-MOF-74(Fe) colloidal solution.

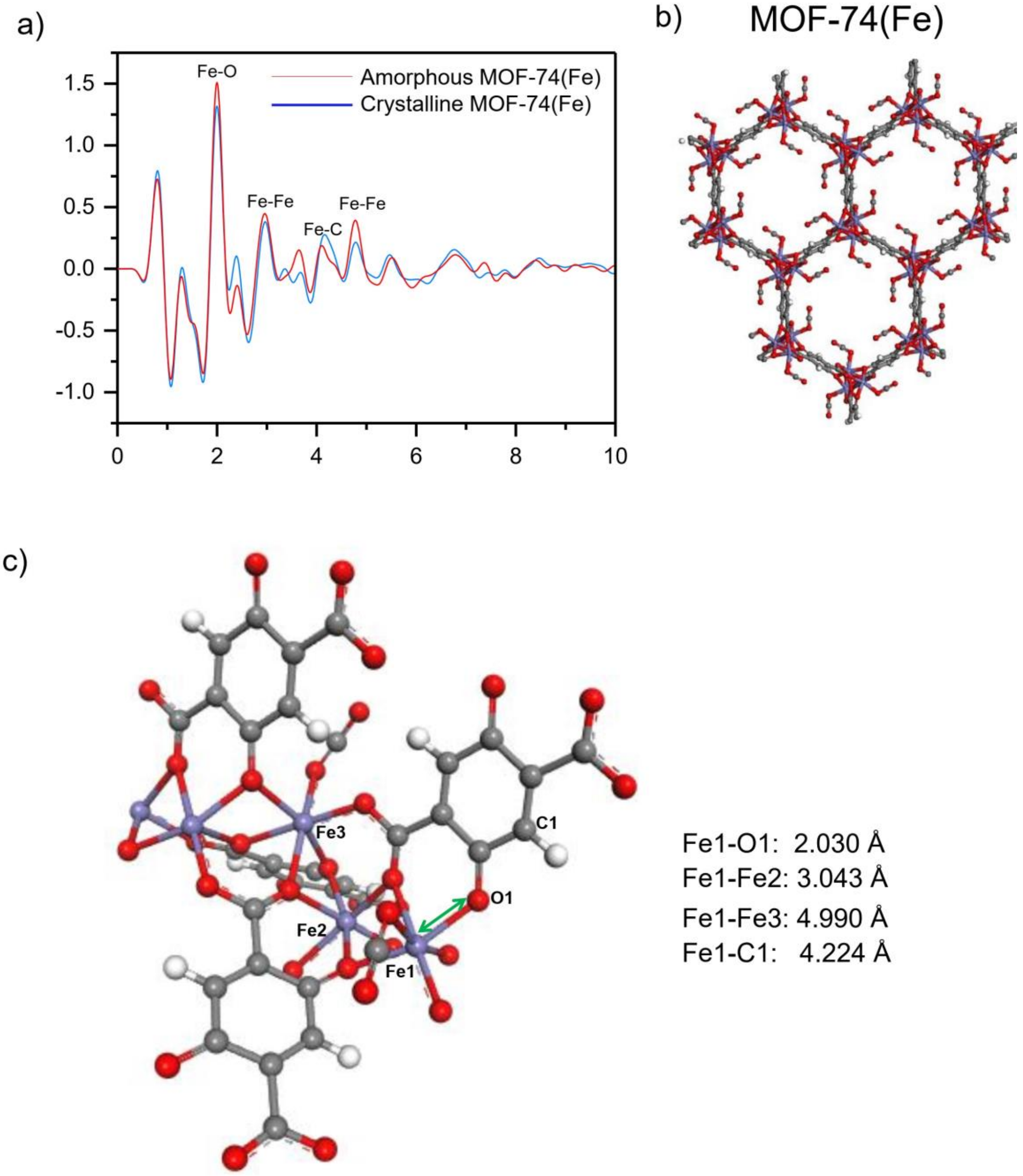

Supplementary Figure 52. (a) Experimental pair distribution functions of a-MOF-74(Fe) and crystalline MOF-74(Fe). (b) Simplified representation of the MOF-74(Fe) crystal structure. (c) Atomic positions model of MOF-74(Fe).

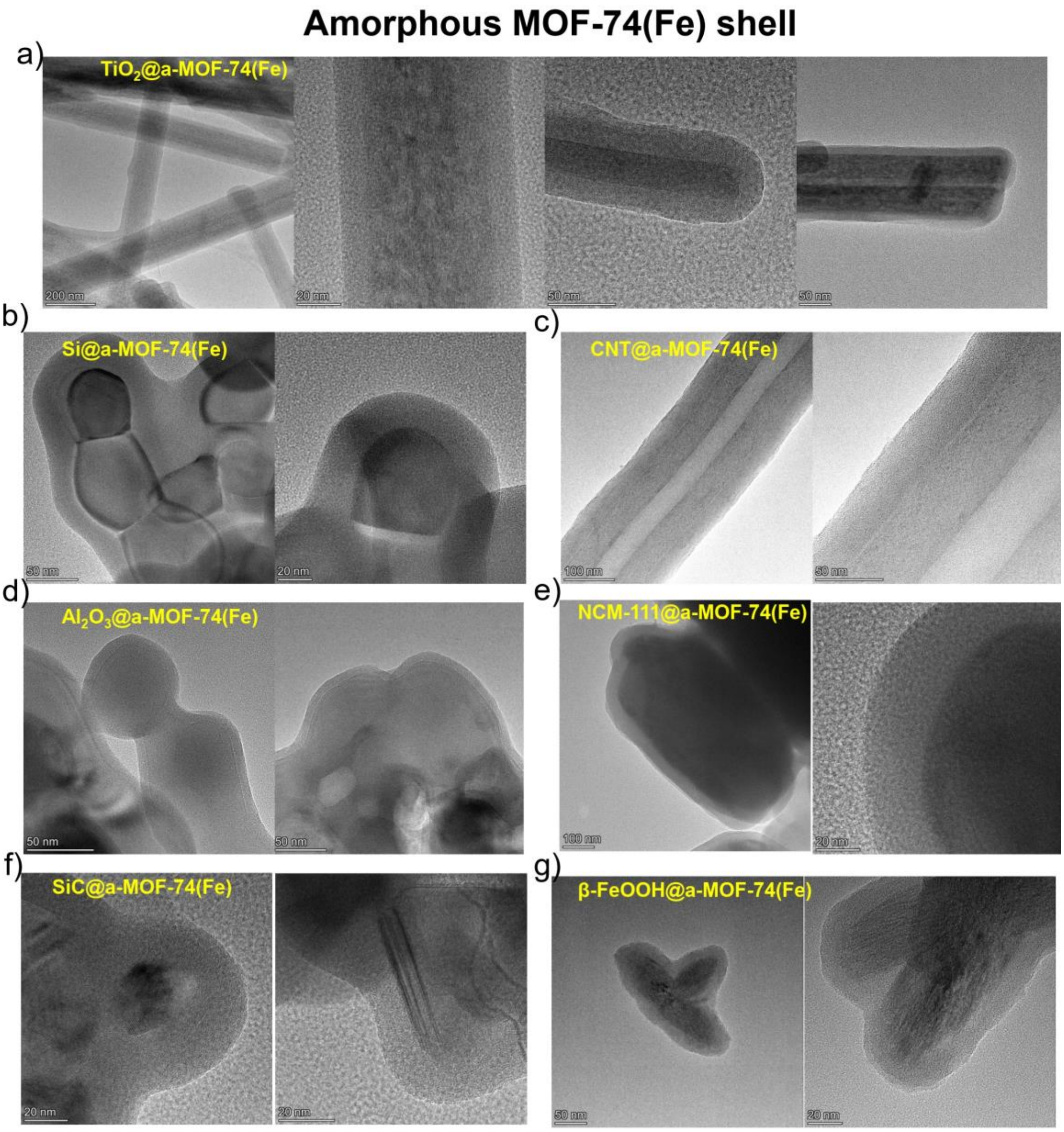

Supplementary Figure 53. (a) TEM images of TiO_2@a-MOF-74. (b) TEM images of Si@ a-MOF-74. (c) TEM images of CNT@ a-MOF-74. (d) TEM images of Al_2O_3@ a-MOF-74. (e) TEM images of NCM-111@a-MOF-74. (f) TEM images of SiC@a-MOF-74. (g) TEM images of β-FeOOH@ a-MOF-74.

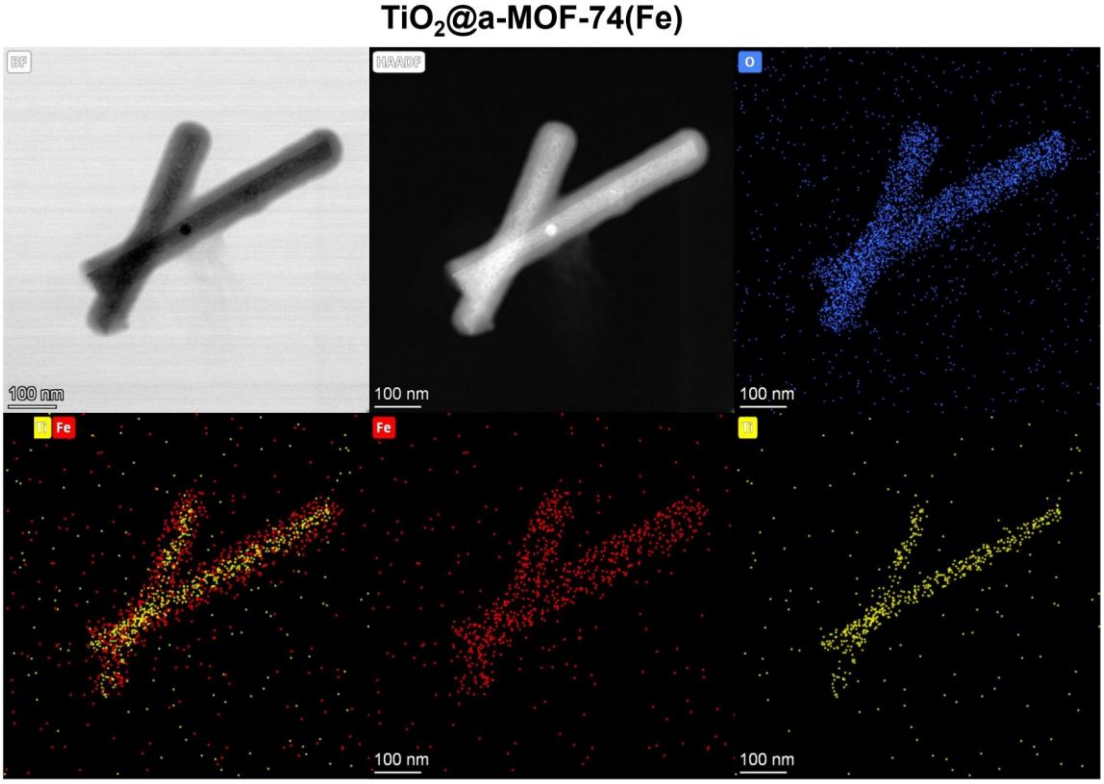

Supplementary Figure 54. Bright Field- and HAADF-STEM images and element mapping images of TiO_2@a-MOF-74(Fe).

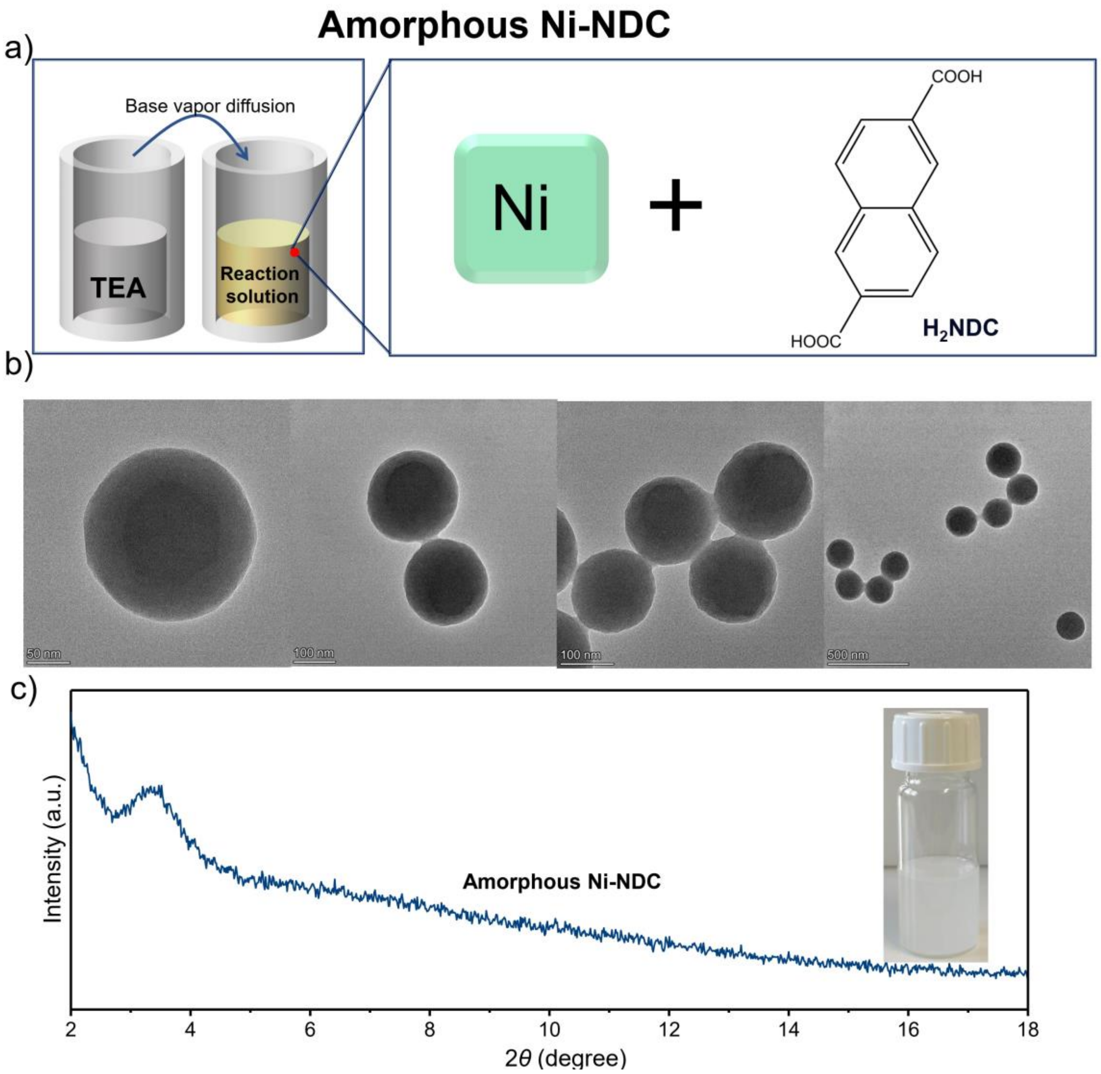

Supplementary Figure 55. (a) Schematic illustration of the preparation of amorphous Ni-NDC using the TEA diffusion method. (b) TEM images of a-Ni-NDC spheres. (c) PXRD pattern of a-Ni-NDC, with an inset showing an optical image of a-Ni-NDC colloidal solution.

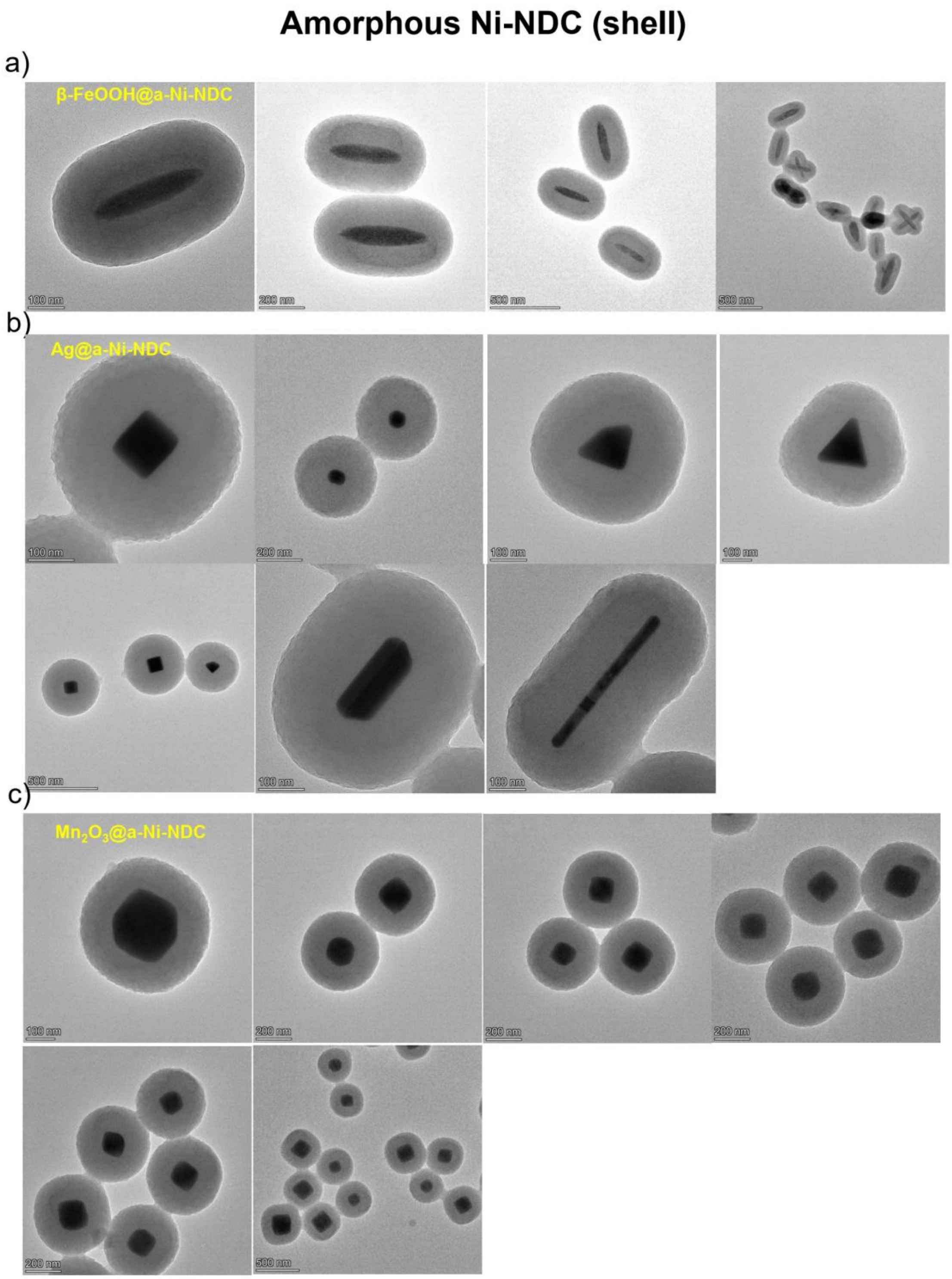

Supplementary Figure 56. (a) TEM images of β-FeOOH@ a-Ni-NDC. (b) TEM images of Ag@ a-Ni-NDC. (c) TEM images of Mn_2O_3@ a-Ni-NDC.

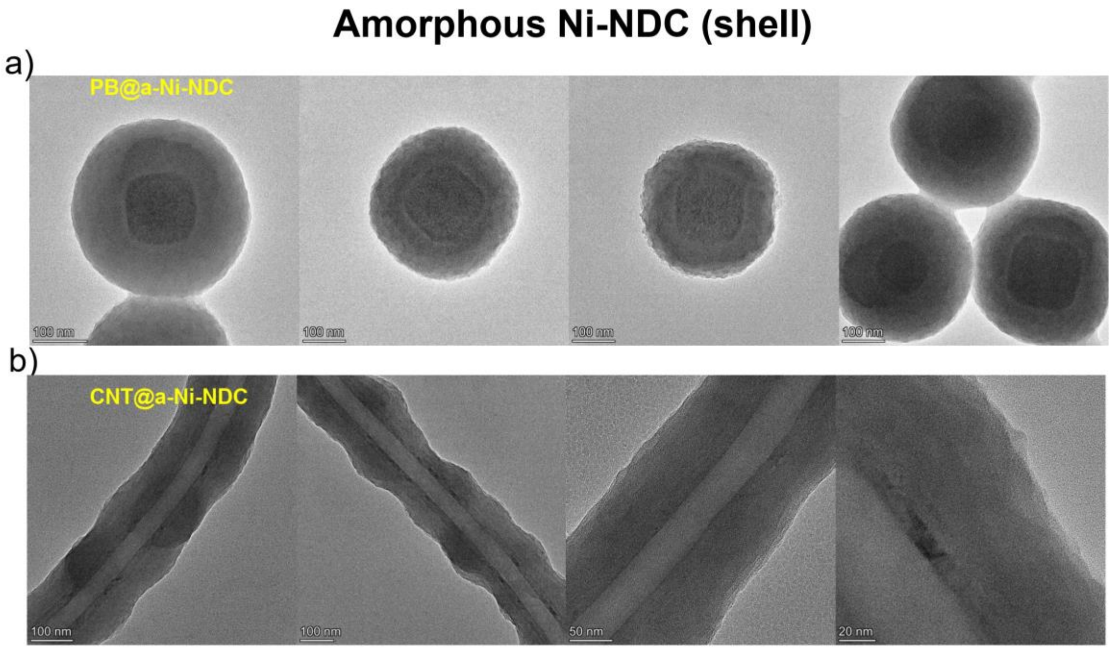

Supplementary Figure 57. (a) TEM images of PB@ a-Ni-NDC. (b) TEM images of CNT@ a-Ni-NDC.

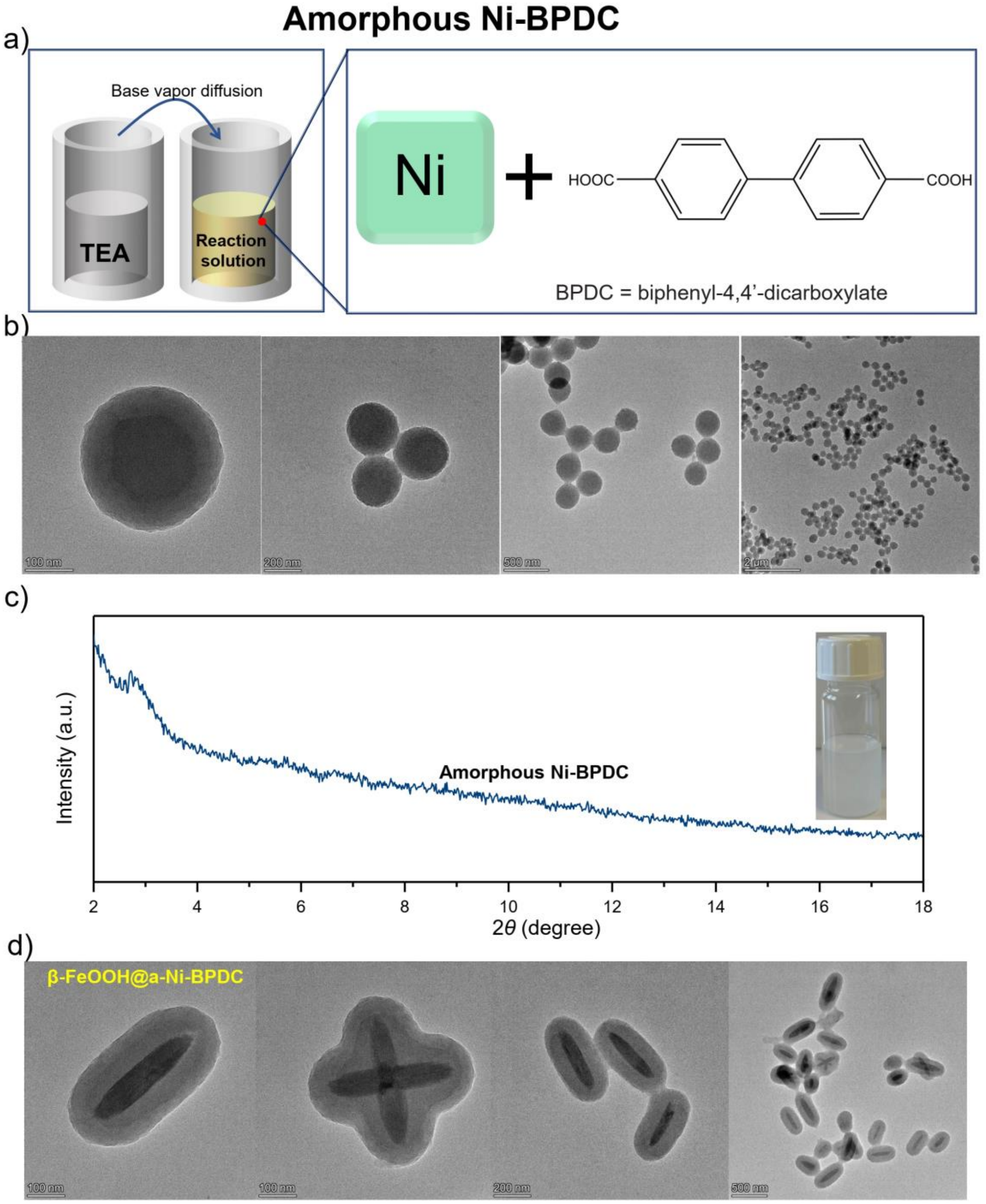

Supplementary Figure 58. (a) Schematic illustration of the preparation of amorphous Ni-BPDC using the TEA diffusion method. (b)TEM images of a-Ni-BPDC spheres. (c) PXRD pattern of a-Ni-BPDC, with an inset showing an optical image of a-Ni-BPDC colloidal solution. (f) TEM images of β-FeOOH@ a-Ni-BPDC.

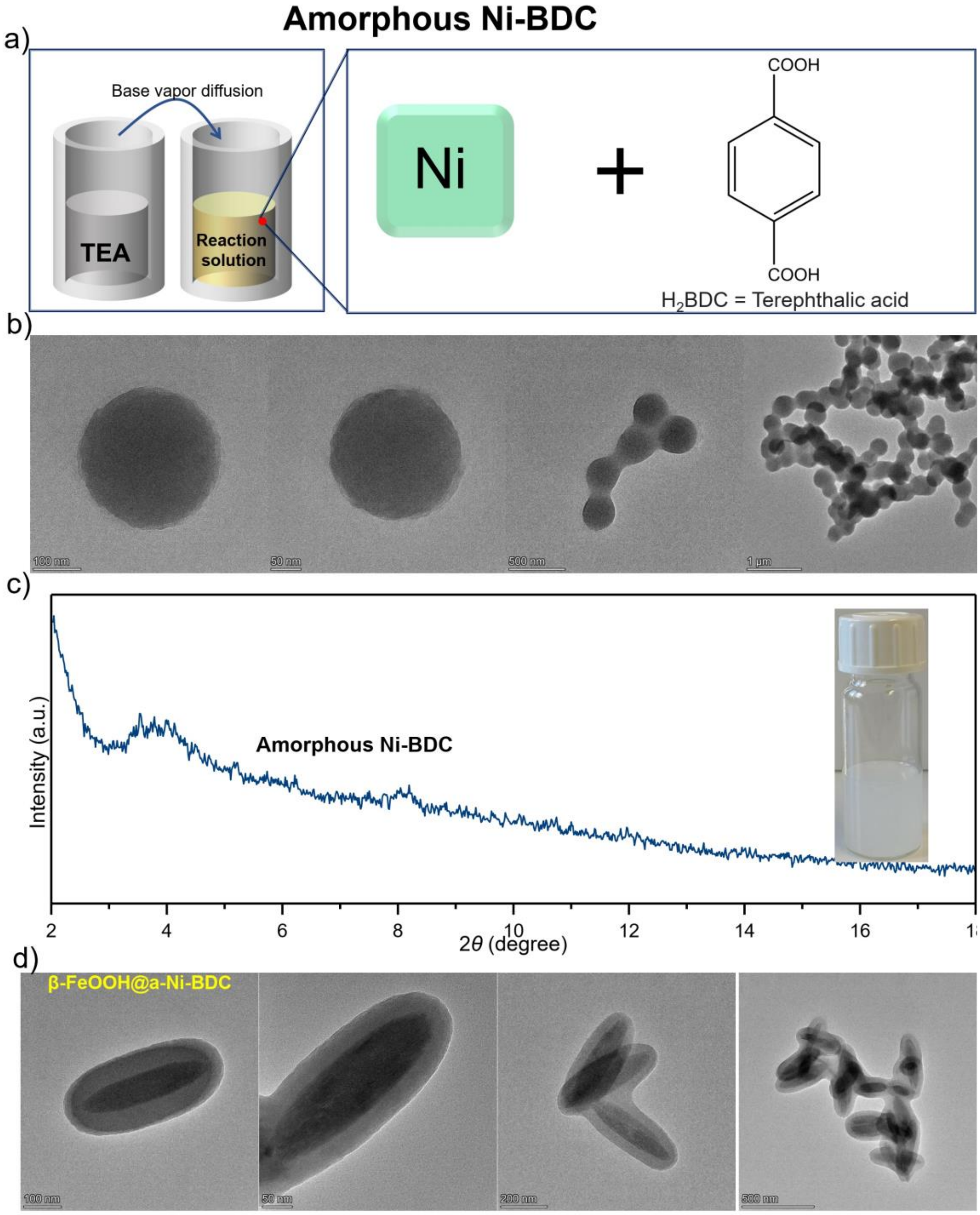

Supplementary Figure 59. (a) Schematic illustration of the preparation of amorphous Ni-BDC using the TEA diffusion method. (b) TEM images of a-Ni-BDC spheres. (c) PXRD pattern of a-Ni-BDC, with an inset showing an optical image of a-Ni-BDC colloidal solution. (d) TEM images of β-FeOOH@ a-Ni-BDC.

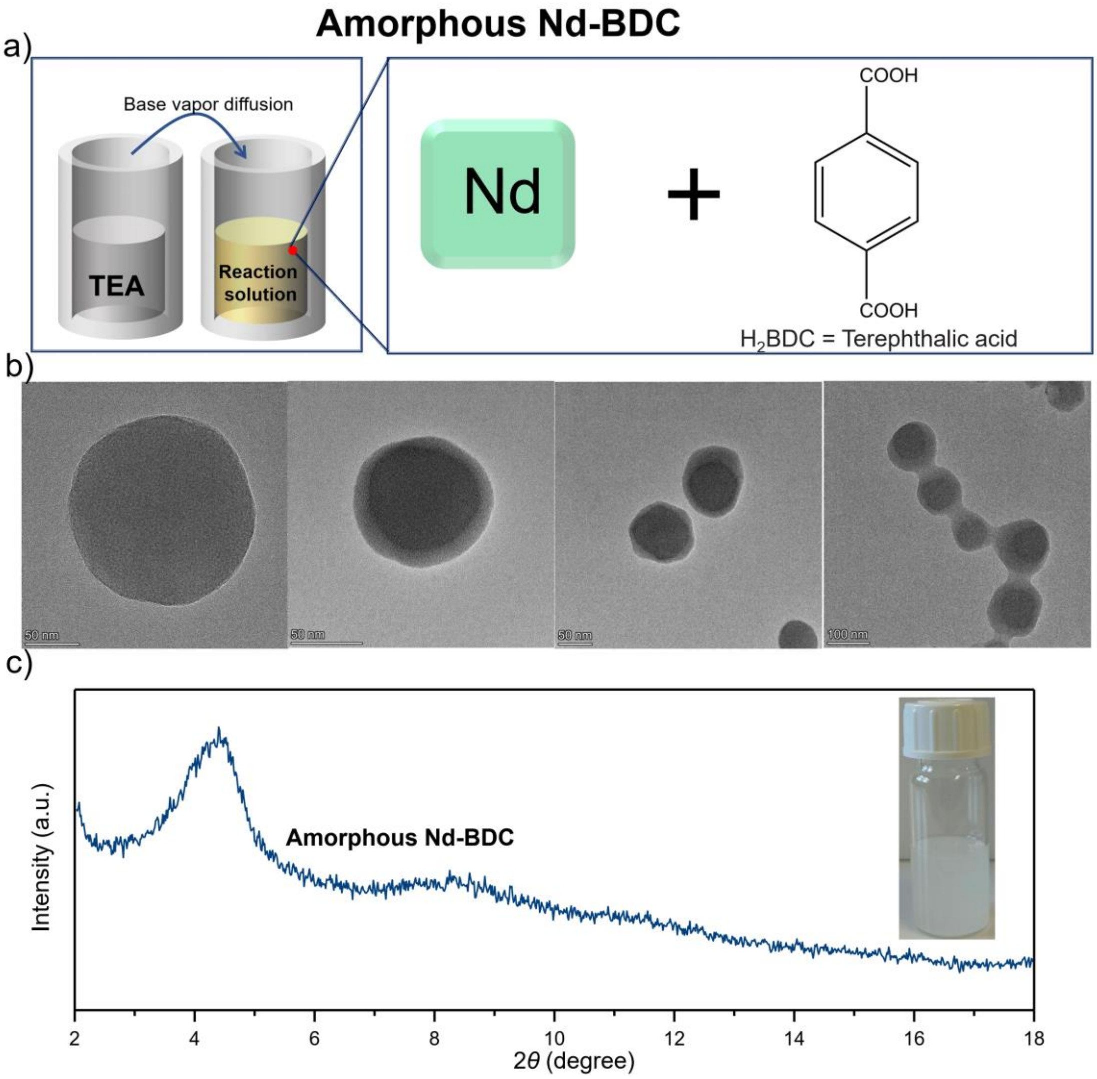

Supplementary Figure 60. (a) Schematic illustration of the preparation of amorphous Nd-BDC using the TEA diffusion method. (b) TEM images of a-Nd-BDC spheres. (c) PXRD pattern of a-Nd-BDC, with an inset showing an optical image of a-Nd-BDC colloidal solution.

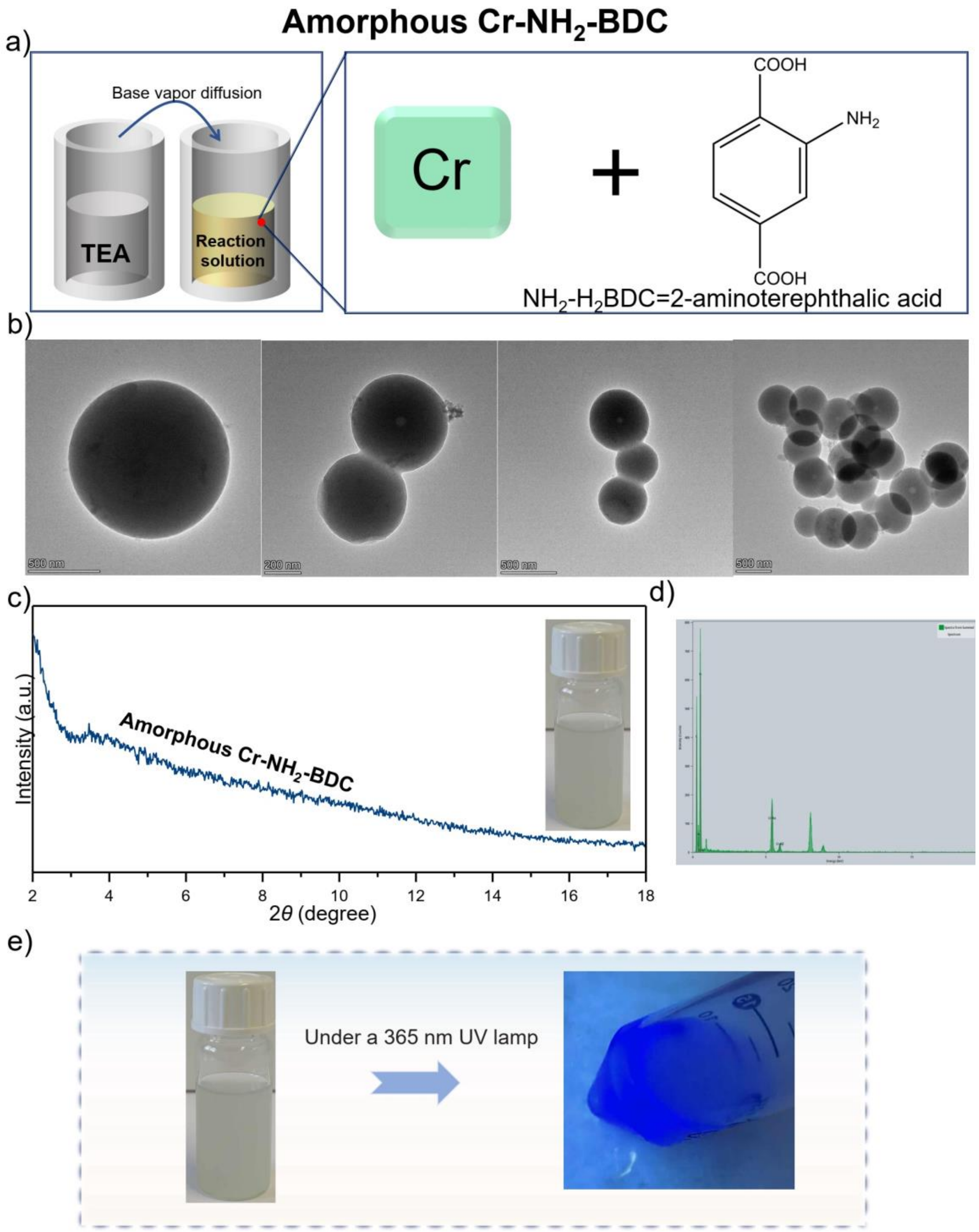

Supplementary Figure 61. (a) Schematic illustration of the preparation of amorphous Cr-NH_2-BDC using the TEA diffusion method. (b) TEM images of a-Cr-NH_2-BDC spheres. (c) PXRD pattern of a-Cr-NH_2-BDC, with an inset showing an optical image of a-Cr-NH_2-BDC colloidal solution. (d) Representative energy dispersive X-ray (EDX) spectrum of amorphous Cr-NH_2-BDC. (e) The corresponding optical images of a-Cr-NH_2-BDC colloidal solution under a 365 nm UV lamp.

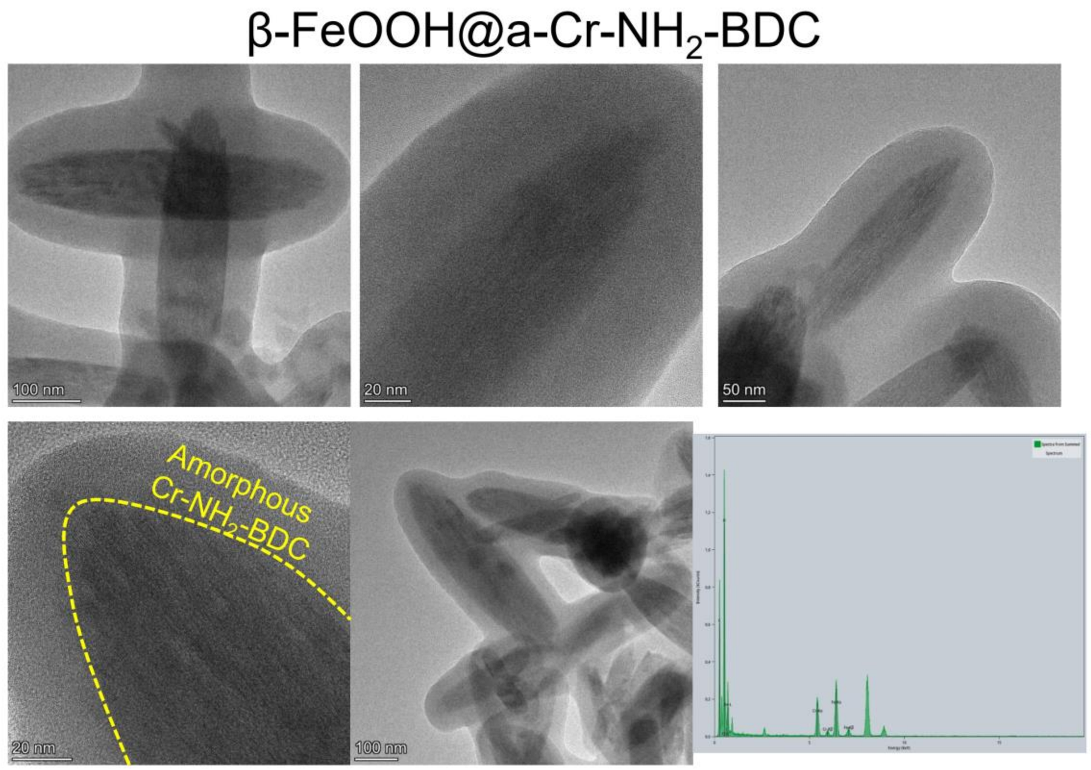

Supplementary Figure 62. TEM images of β-FeOOH@a-Cr-NH2-BDC and the representative energy dispersive X-ray (EDX) spectrum of β-FeOOH@a-Cr-NH2-BDC.

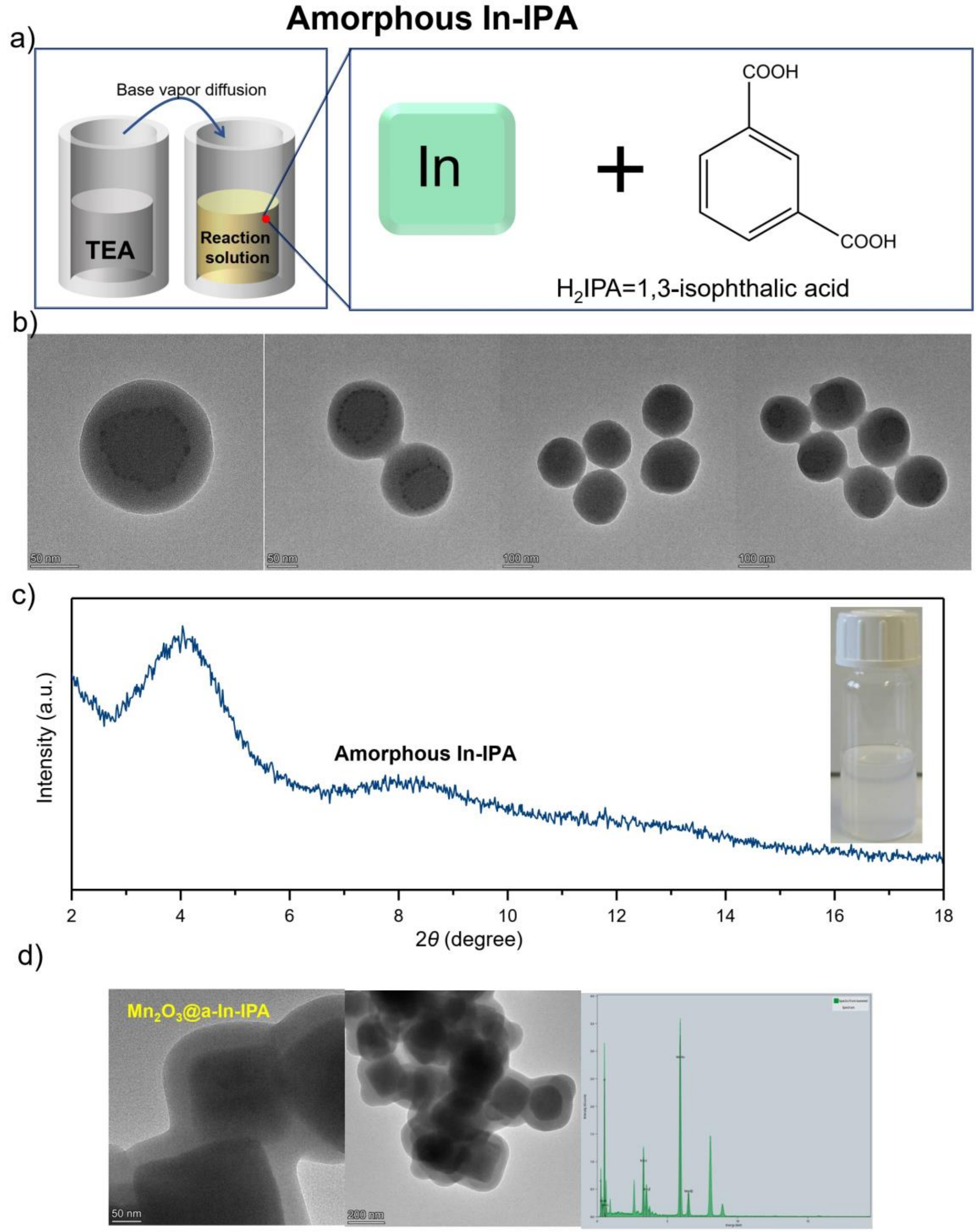

Supplementary Figure 63. (a) Schematic illustration of the preparation of amorphous In-IPA using the TEA diffusion method. (b) TEM images of a-In-IPA spheres. (c) PXRD pattern of a-In-IPA, with an inset showing an optical image of a-In-IPA colloidal solution. (d) TEM images and representative energy dispersive X-ray (EDX) spectrum of Mn_2O_3@a-In-IPA.

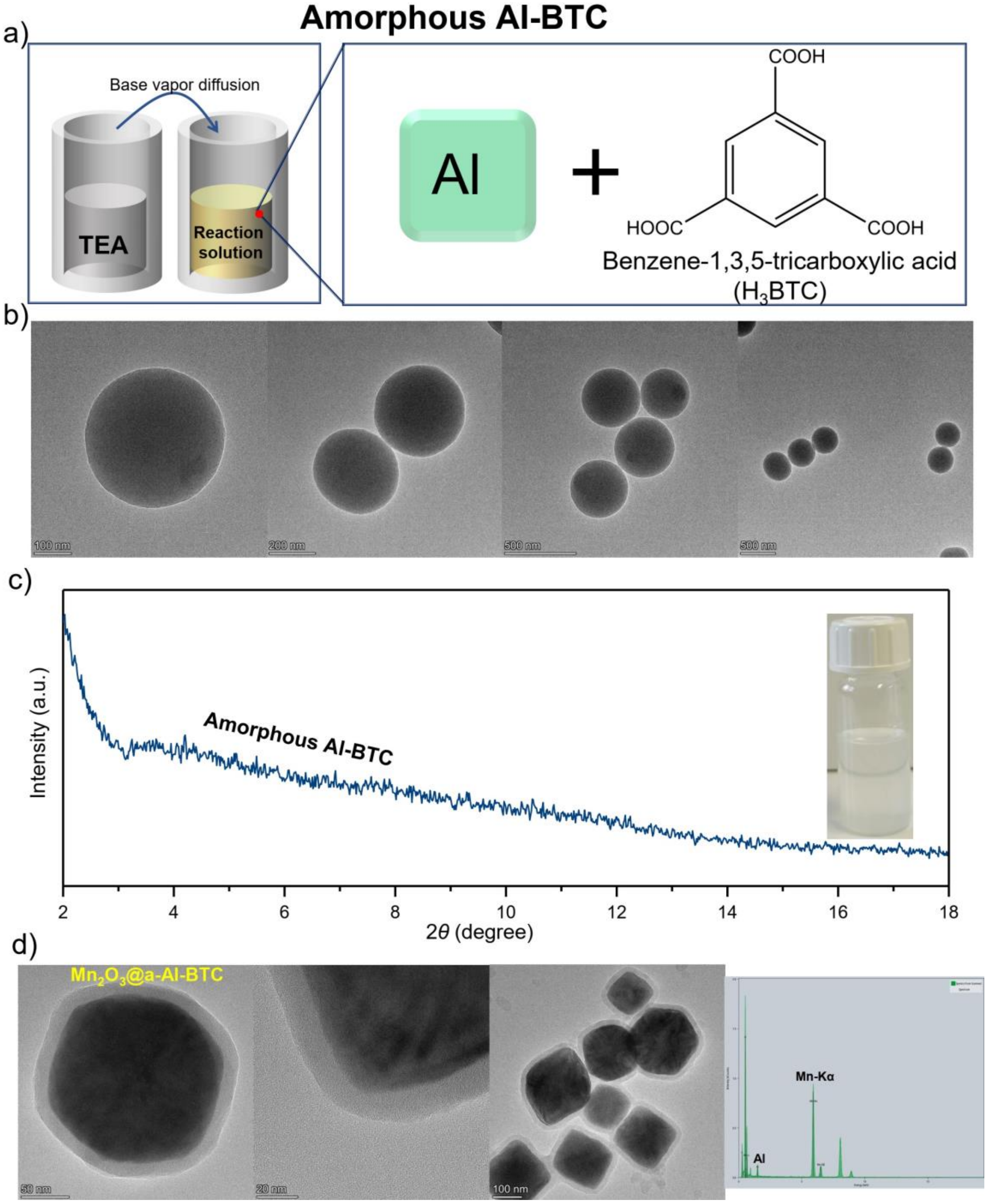

Supplementary Figure 64. (a) Schematic illustration of the preparation of amorphous Al-BTC using the TEA diffusion method. (b) TEM images of a-Al-BTC spheres. (c) PXRD pattern of a-Al-BTC, with an inset showing an optical image of a-Al-BTC colloidal solution. (d) TEM images and representative energy dispersive X-ray (EDX) spectrum of Mn_2O_3@a-Al-BTC.

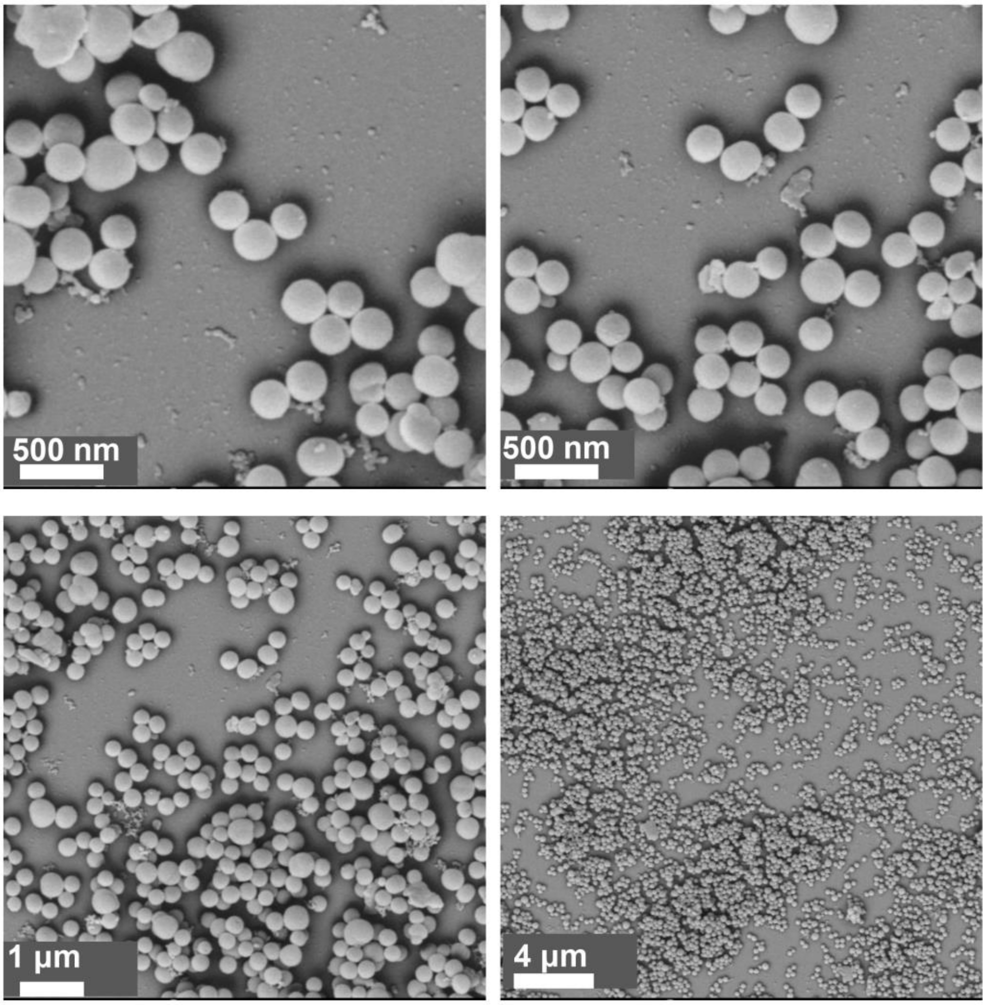

Supplementary Figure 65. SEM images of a-Al-BTC spheres with different magnifications using ammonia solution as base generator.

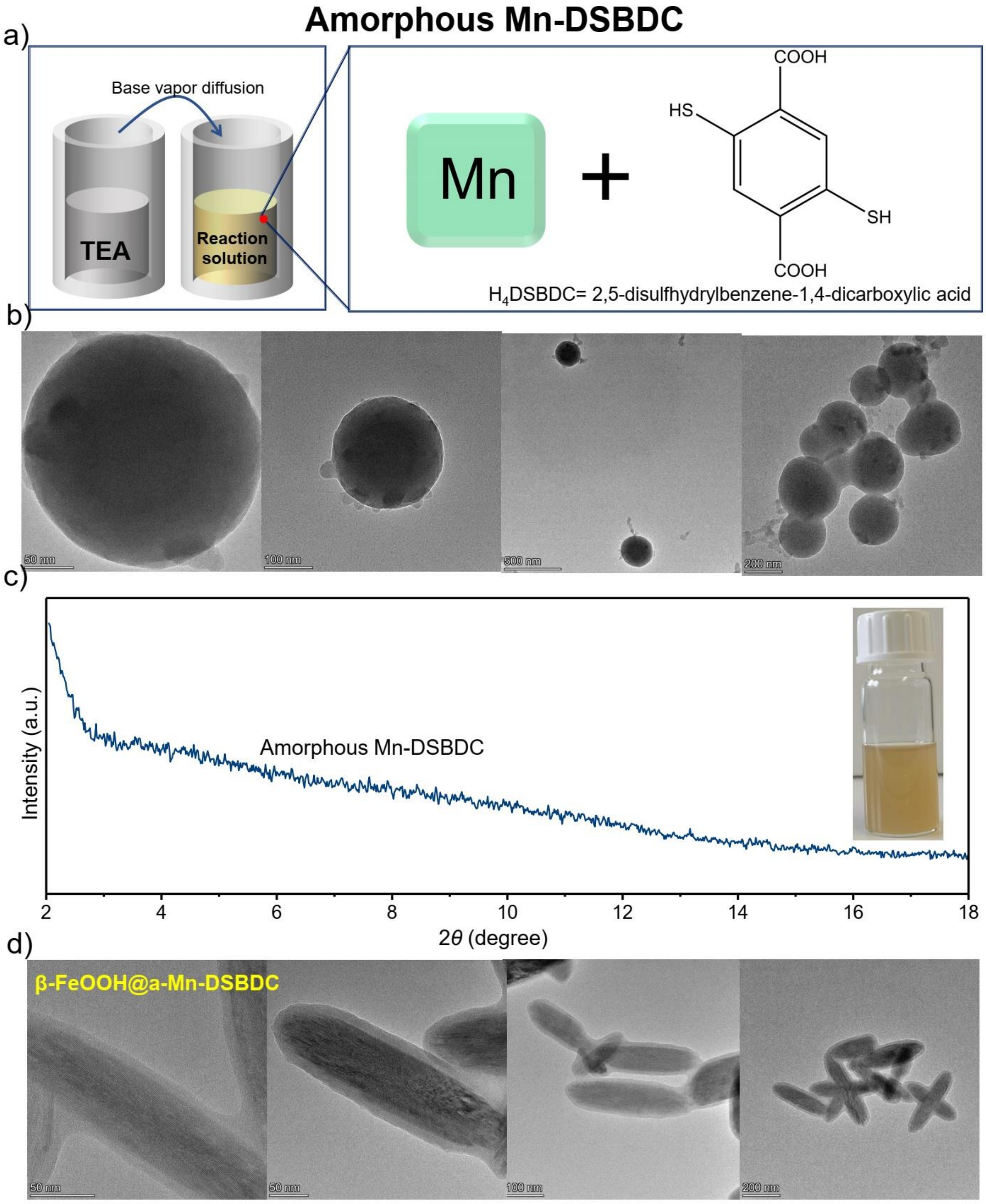

Supplementary Figure 66. (a) Schematic illustration of the preparation of amorphous Mn-DSBDC using the TEA diffusion method. (b) TEM images of a-Mn-DSBDC spheres. (c) PXRD pattern of a-Mn-DSBDC, with an inset showing an optical image of a-Mn-DSBDC colloidal solution. (d) TEM images of β-FeOOH@a-Mn-DSBDC.

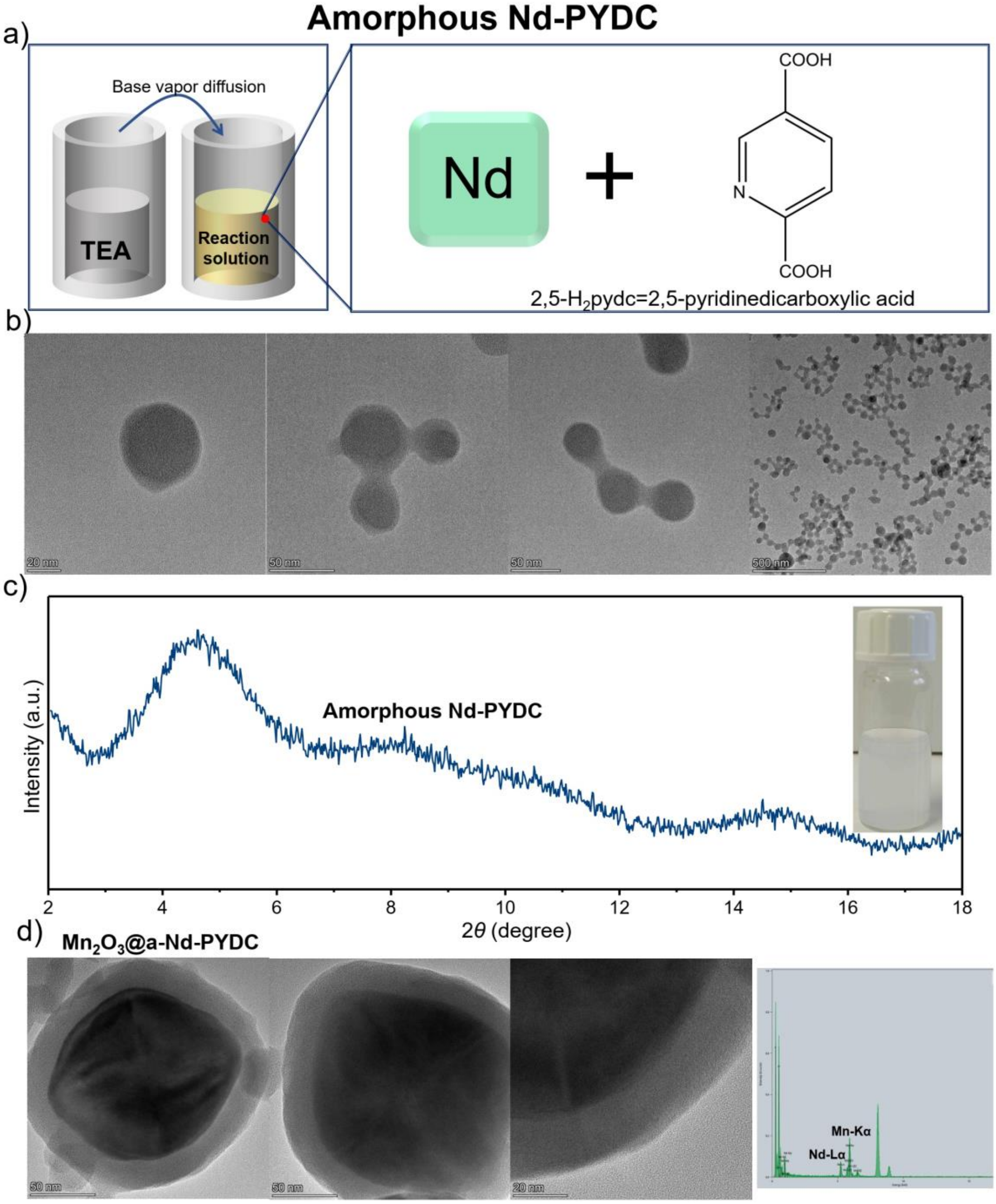

Supplementary Figure 67. (a) Schematic illustration of the preparation of amorphous Nd-PYDC using the TEA diffusion method. (b) TEM images of a- Nd-PYDC spheres. (c) PXRD pattern of a-Nd-PYDC, with an inset showing an optical image of a-Nd-PYDC colloidal solution. (d) TEM images and representative energy dispersive X-ray (EDX) spectrum of Mn_2O_3@a-Nd-PYDC.

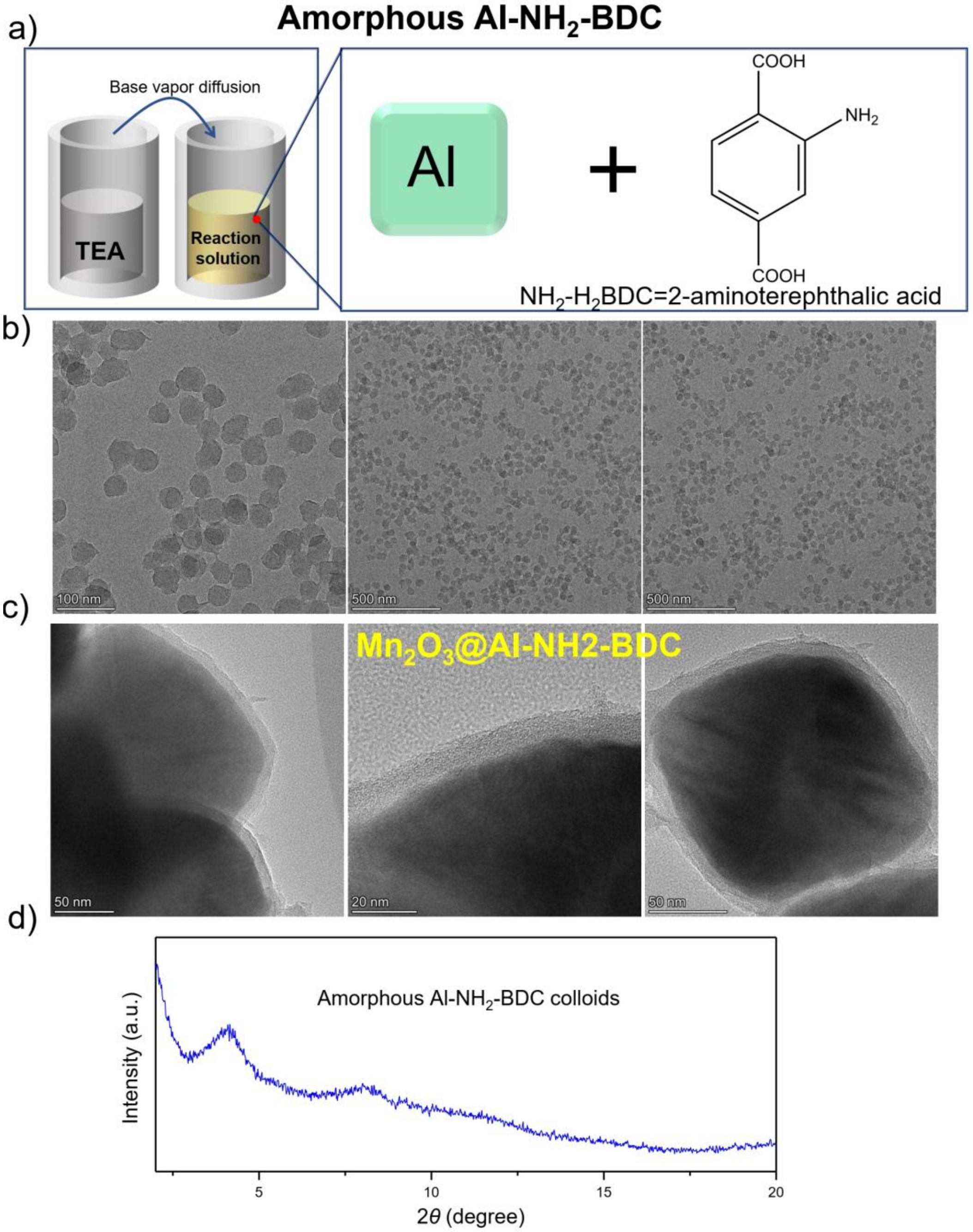

Supplementary Figure 68. (a) Schematic illustration of the preparation of amorphous Al-NH_2-BDC using the TEA diffusion method. (b) TEM images of a-Al-NH_2-BDC colloids. (c) TEM images of Mn_2O_3@a- Al-NH_2-BDC. (d) PXRD pattern of a-Al-NH_2-BDC colloids.

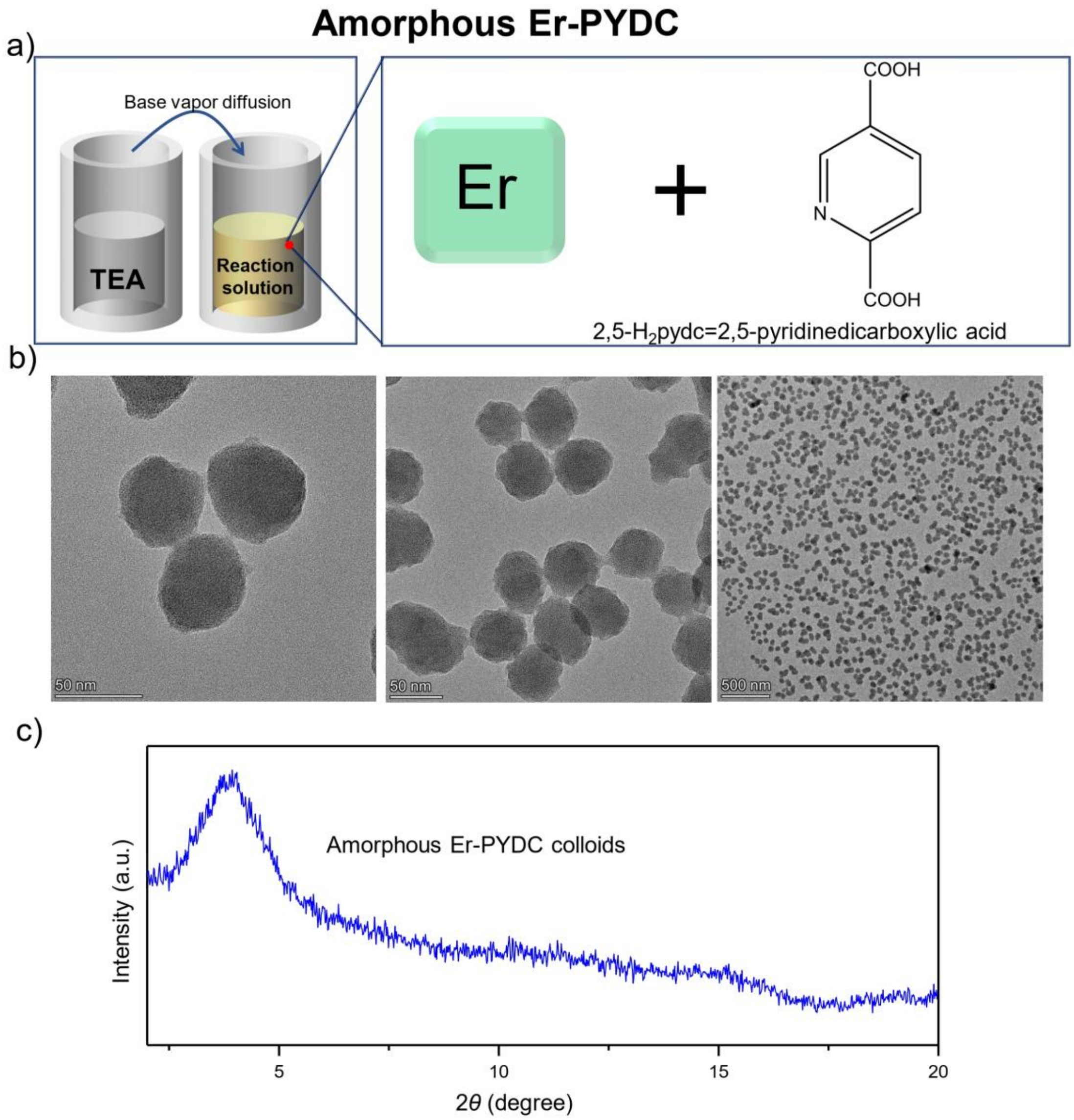

Supplementary Figure 69. (a) Schematic illustration of the preparation of amorphous Er-PYDC using the TEA diffusion method. (b) TEM images of a- Er-PYDC colloids. (c) PXRD pattern of a-Er-PYDC colloids.

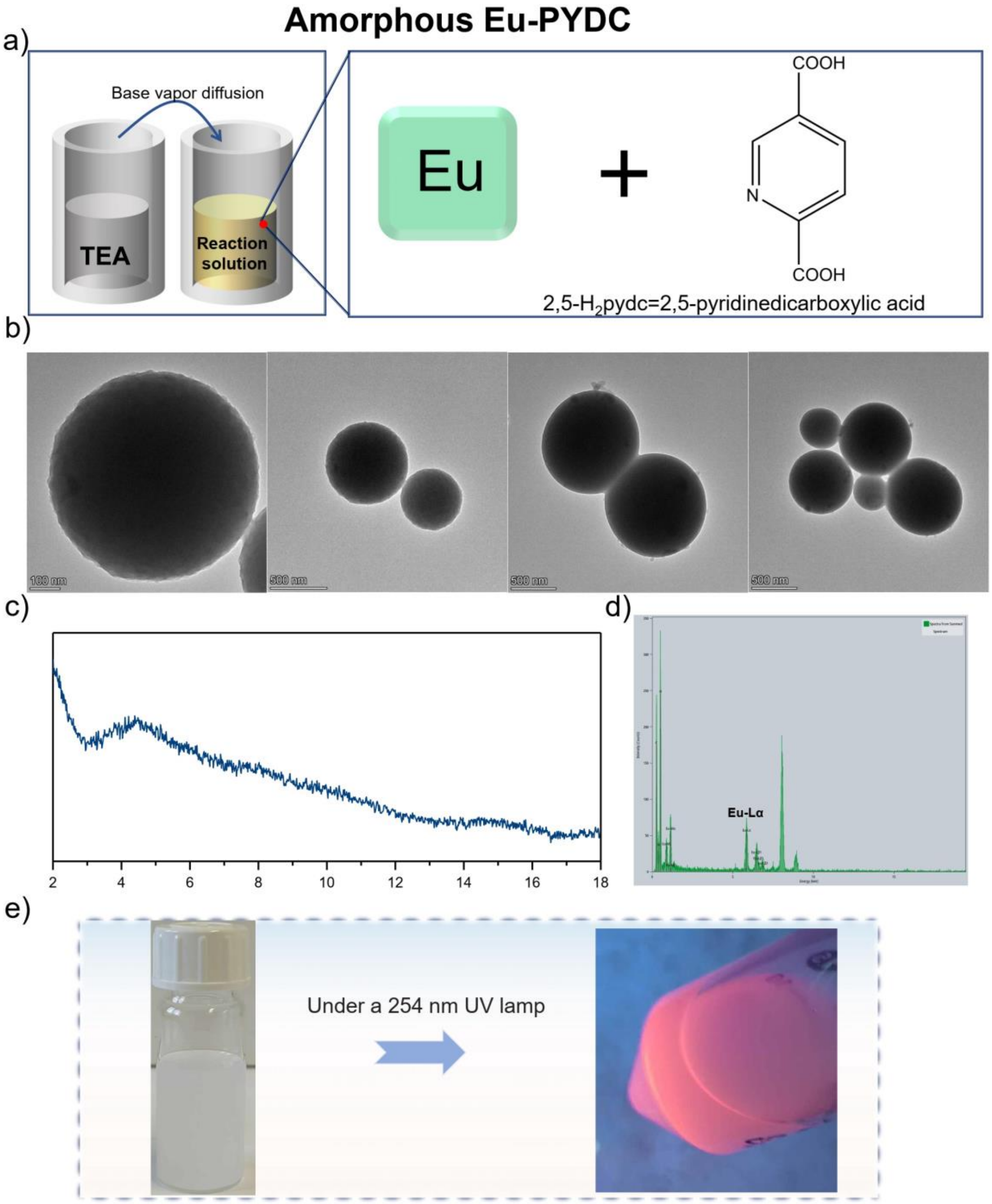

Supplementary Figure 70. (a) Schematic illustration of the preparation of amorphous Eu-PYDC using the TEA diffusion method. (b) TEM images of a-Eu-PYDC spheres. (c) PXRD pattern of a-Eu-PYDC, with an inset showing an optical image of a-Eu-PYDC colloidal solution. (d) Representative energy dispersive X-ray (EDX) spectrum of amorphous Eu-PYDC. (e) The corresponding optical images of a-Eu-PYDC colloidal solution under a 254 nm UV lamp.

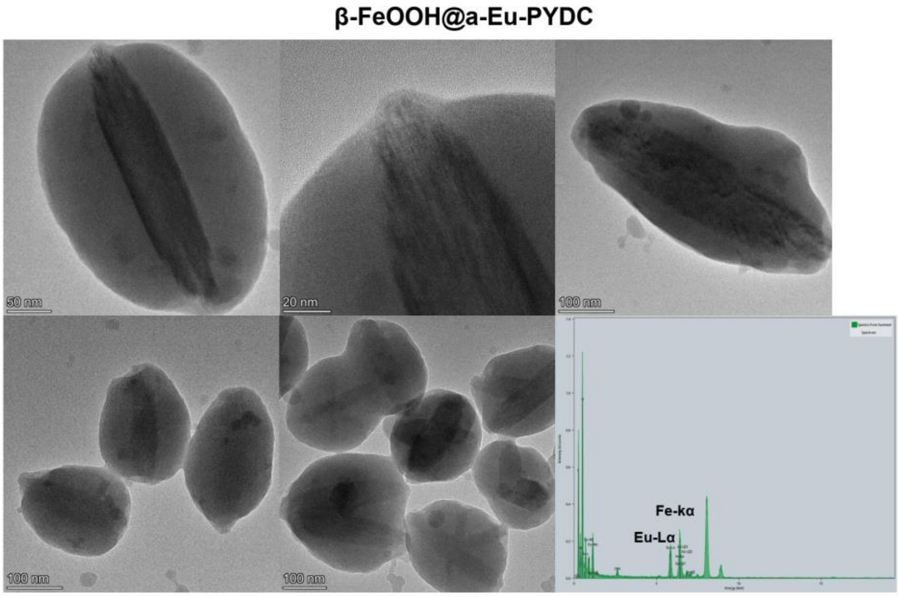

Supplementary Figure 71. TEM images of β-FeOOH@a-Eu-PYDC and the representative energy dispersive X-ray (EDX) spectrum of -FeOOH@a-Eu-PYDC.

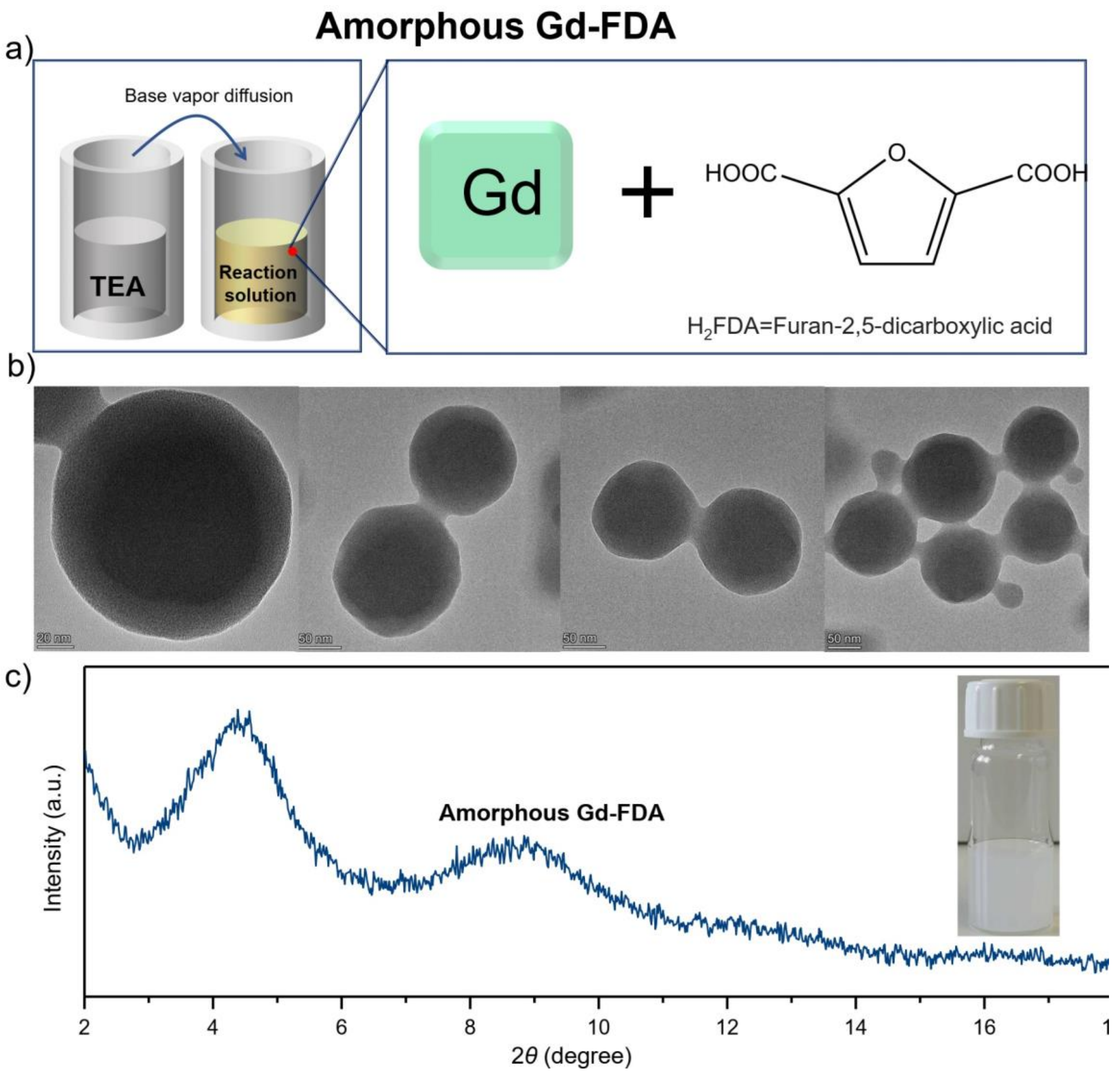

Supplementary Figure 72. (a) Schematic illustration of the preparation of amorphous Gd-FDA using the TEA diffusion method. (b) TEM images of a-Gd-FDA spheres. (c) PXRD pattern of a-Gd-FDA, with an inset showing an optical image of a-Gd-FDA colloidal solution.

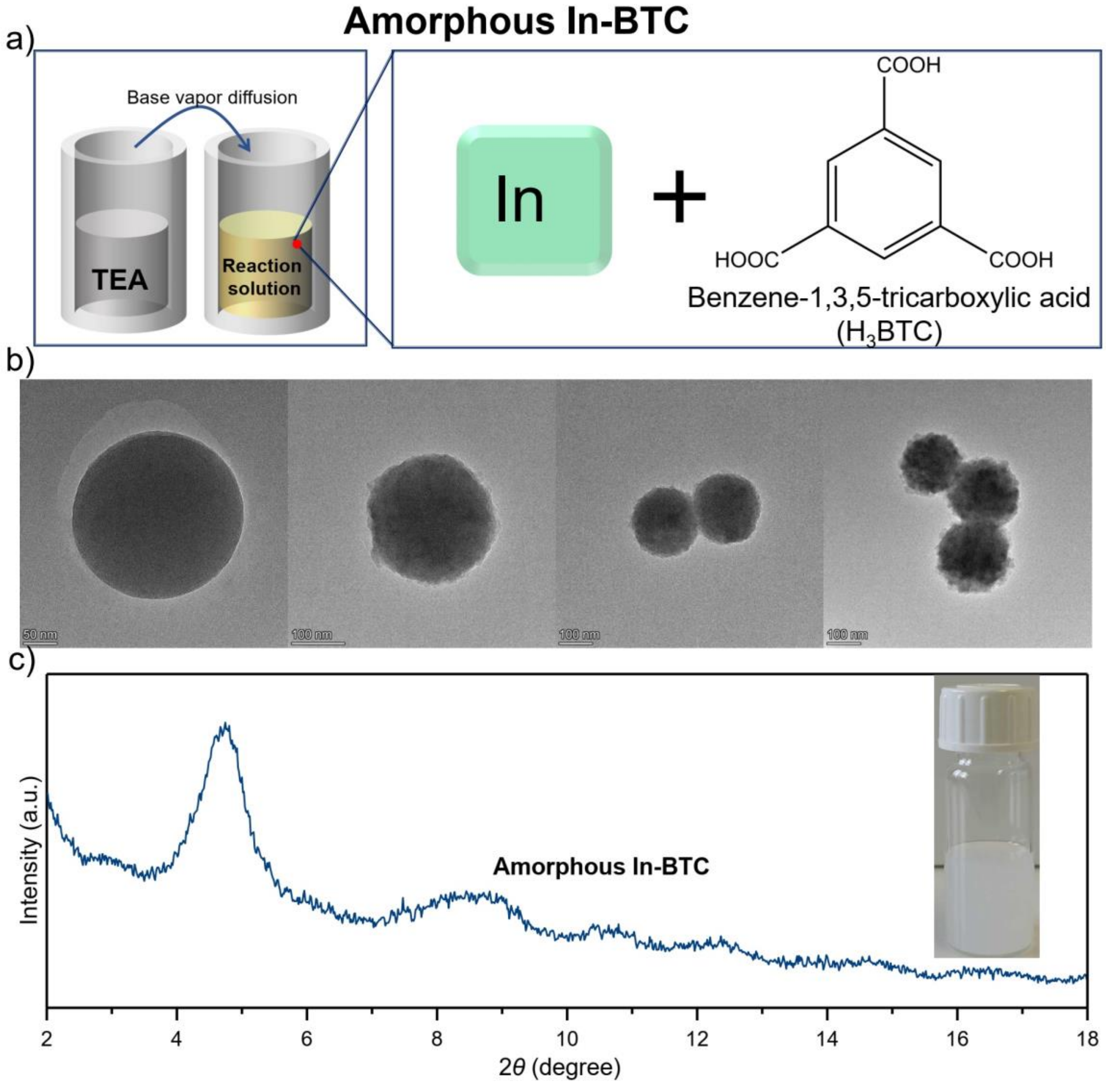

Supplementary Figure 73. (a) Schematic illustration of the preparation of amorphous In-BTC using the TEA diffusion method. (b) TEM images of a-In-BTC spheres. (c) PXRD pattern of a-In-BTC, with an inset showing an optical image of a-In-BTC colloidal solution.

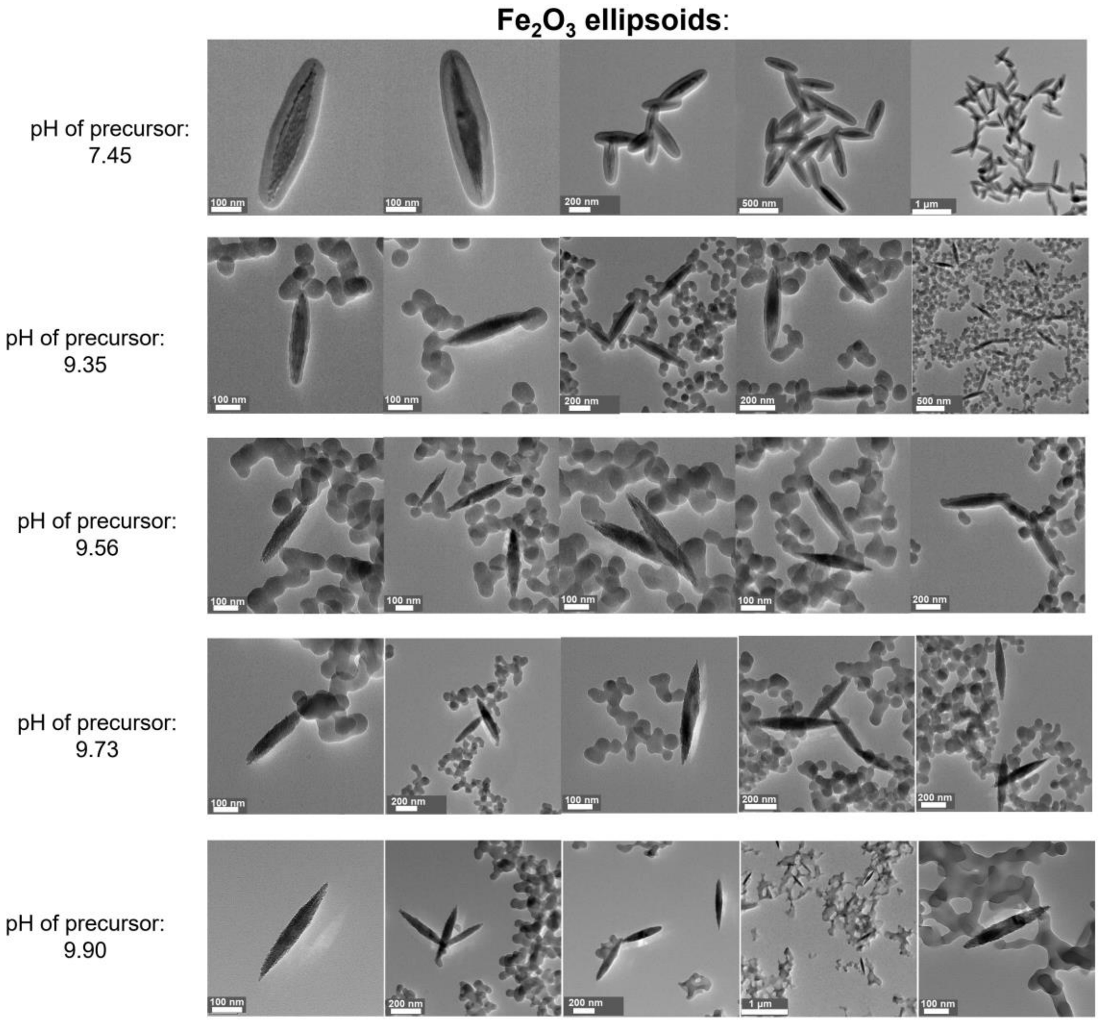

Supplementary Figure 74. TEM images of Fe_2O_3 ellipsoids@aZIF-7 obtained by varying the pH of the precursor solution.

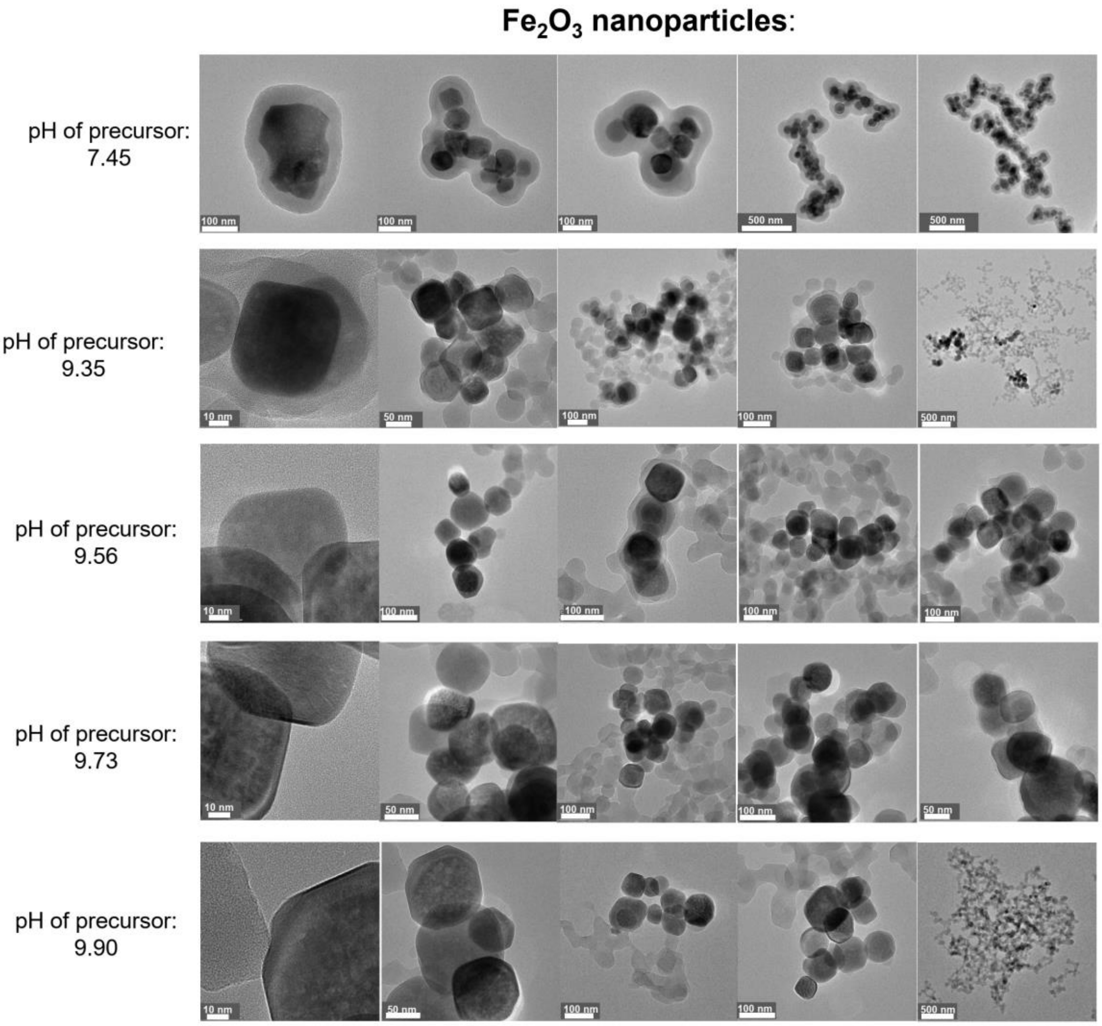

Supplementary Figure 75. TEM images of Fe_2O_3 NPs@aZIF-7 obtained by varying the pH of the precursor solution.

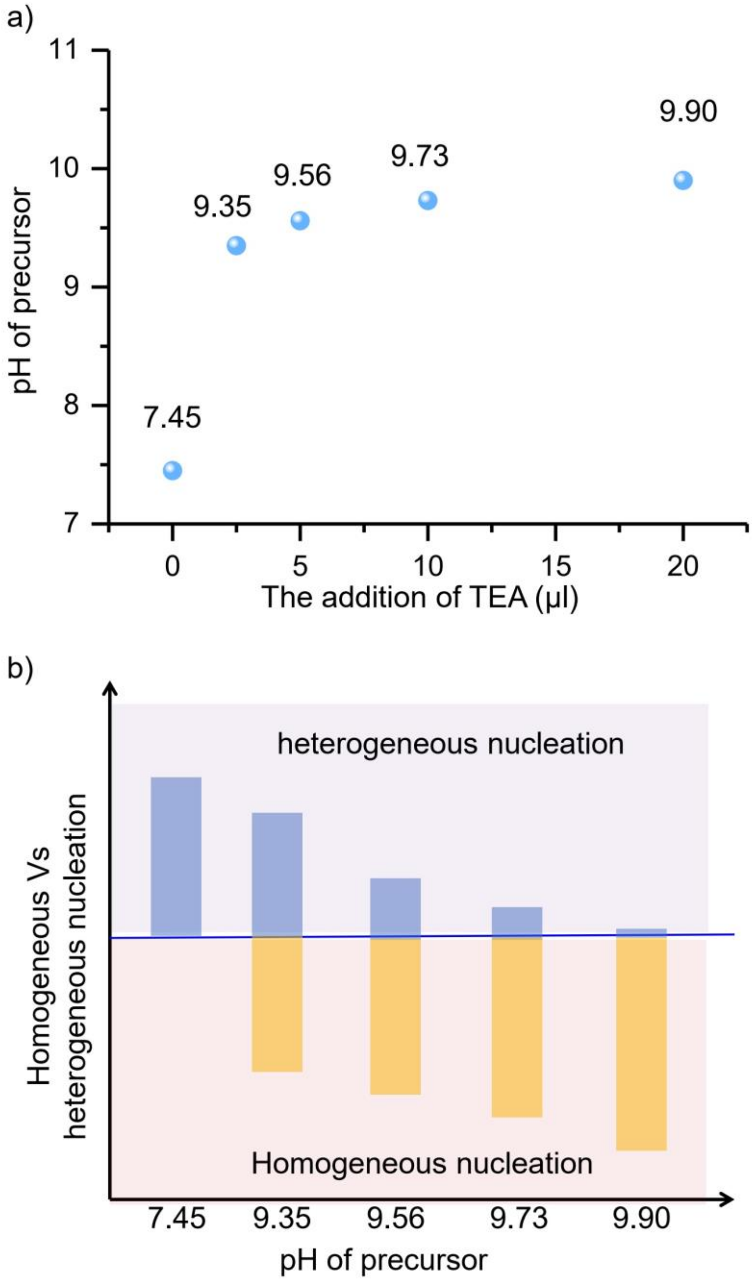

Supplementary Figure 76. (a) The plot of the addition of TEA versus pH of the precursor solution. (b) The schematic illustration of the pH effect on the types of nucleation.

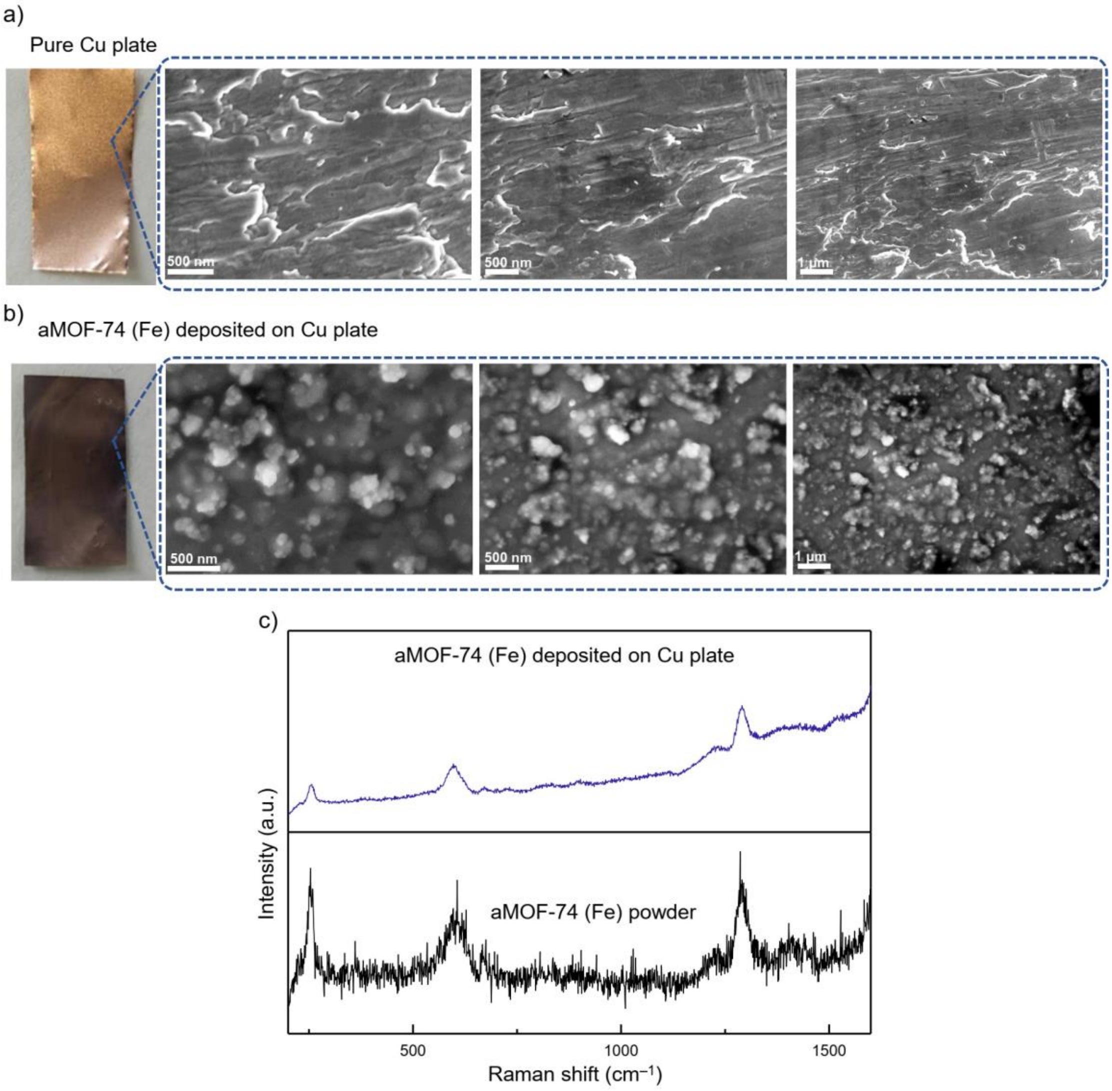

Supplementary Figure 77. (a) The optical and SEM images of pure Cu plate before. (b) The optical and SEM images of Cu plate after aMOF-74(Fe) deposition. (c) The Raman spectra of a-MOF-74(Fe) powder and Cu plate after aMOF-74(Fe) deposition.

These SEM images and Raman spectra indicate the a-MOF-74(Fe) was successfully deposited on Cu plate.

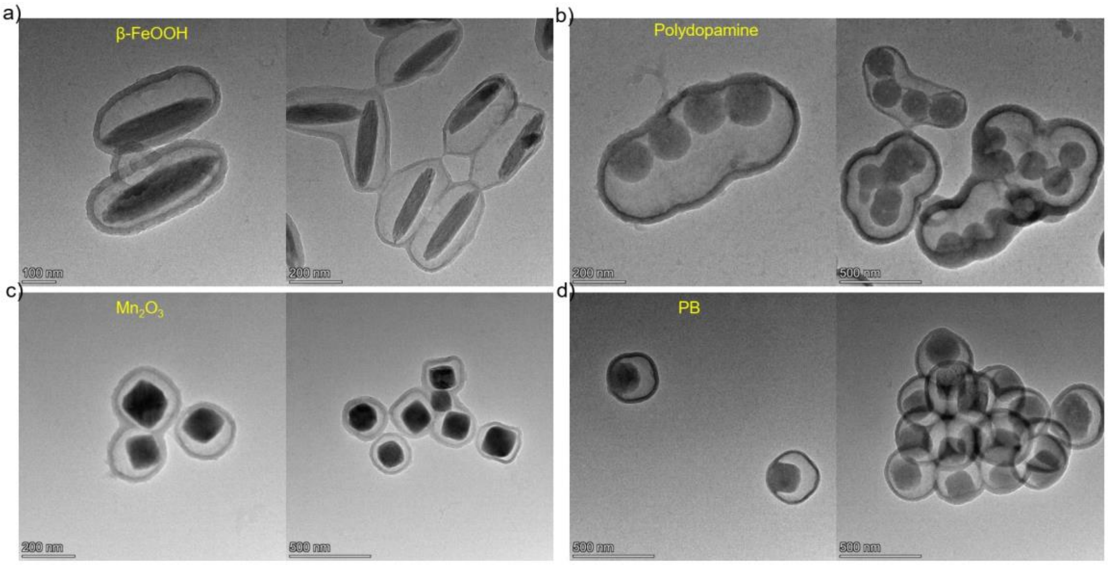

Supplementary Figure 78. TEM images of various yolk-shell structures. (a) Conformal β-FeOOH@void@aZIF-zni. (b) Hollow SiO_2@void@aZIF-zni. (c) Mn2O3@void@aZIF-zni. (d) polydopamine@void@-aZIF-zni.

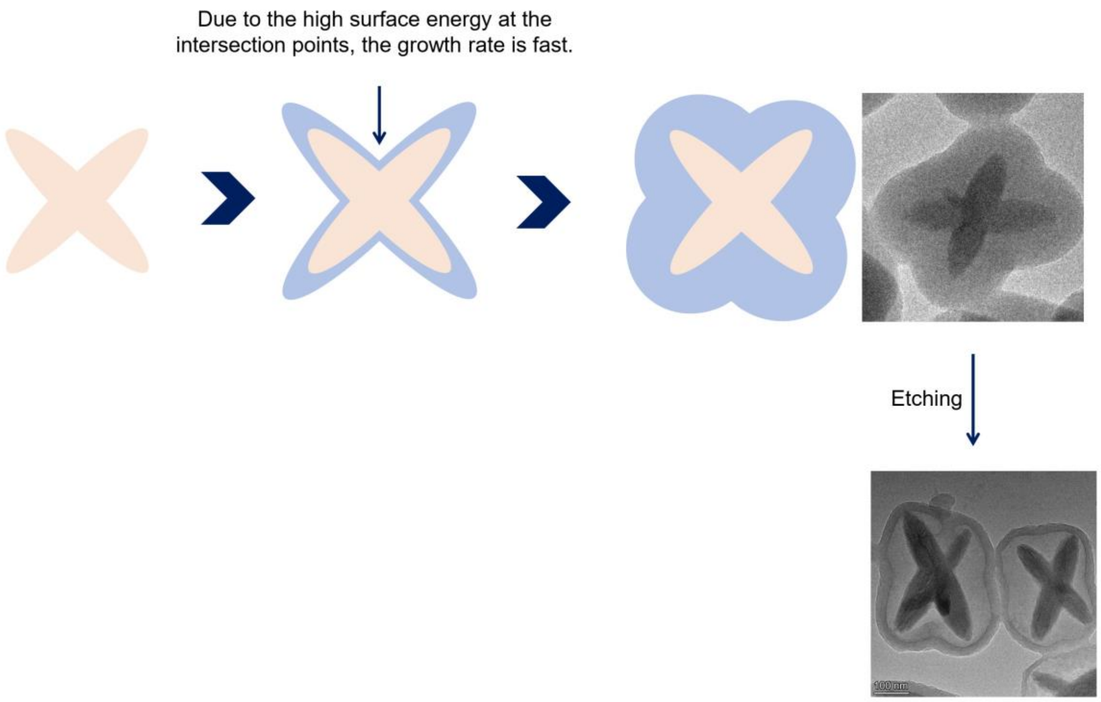

Supplementary Figure 79. Schematic illustration of the formation of yolk-shell structures of X-shape β-FeOOH@void@aZIF-zni. Due to the higher surface energy at intersection points, the growth rate of aMOFs is faster, leading to the formation of nearly square core-shell structures. After a controllable etching process, cubic-like yolk-shell structures can be obtained.

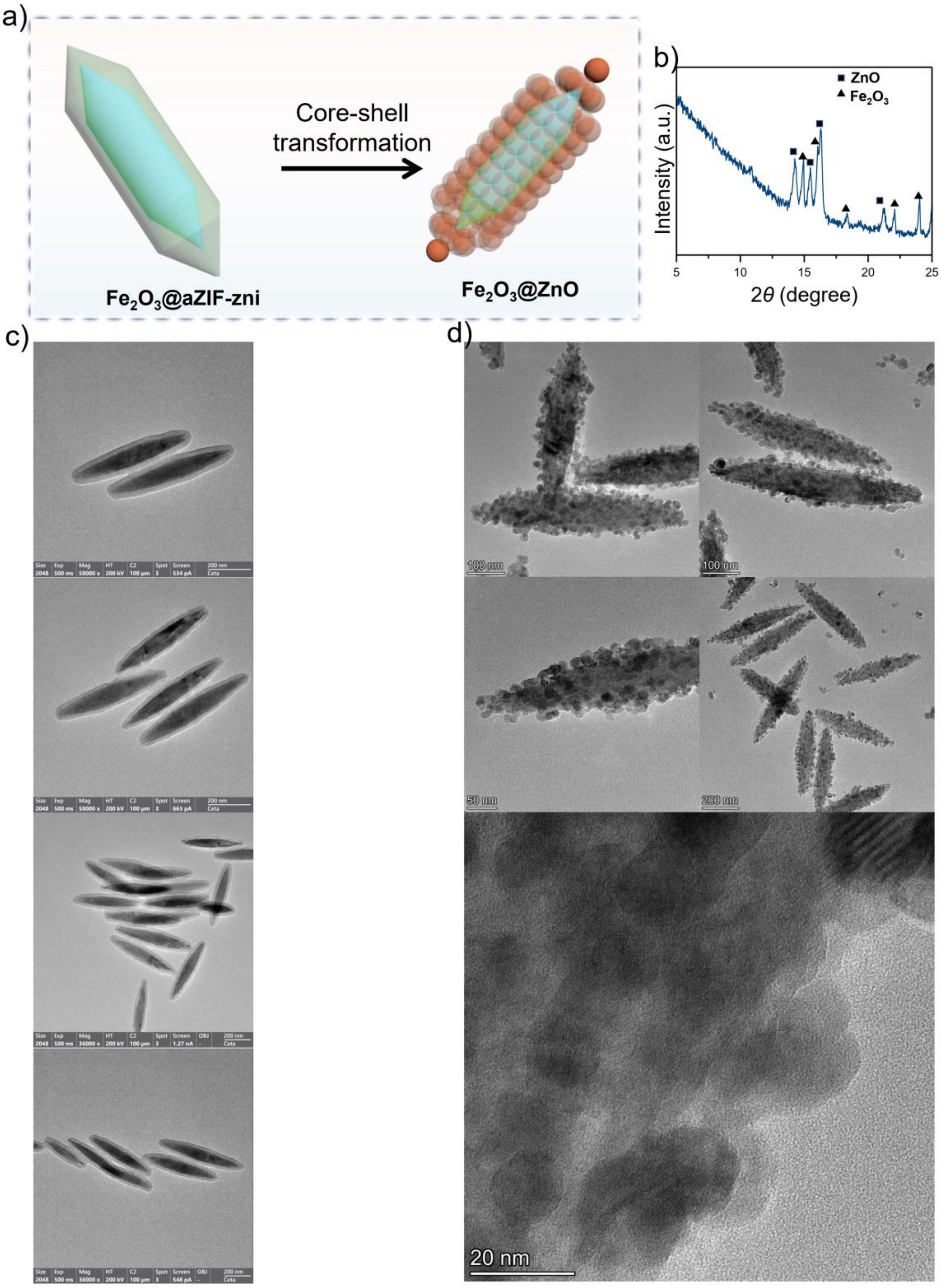

Supplementary Figure 80. (a) Schematic illustration of the transformation of Fe_2O_3@aZIF-zni into Fe_2O_3@ZnO. (b) PXRD pattern of the Fe_2O_3@ZnO colloids. (c) TEM images of the Fe_2O_3@aZIF-zni. (d) TEM images of the Fe_2O_3@ZnO.

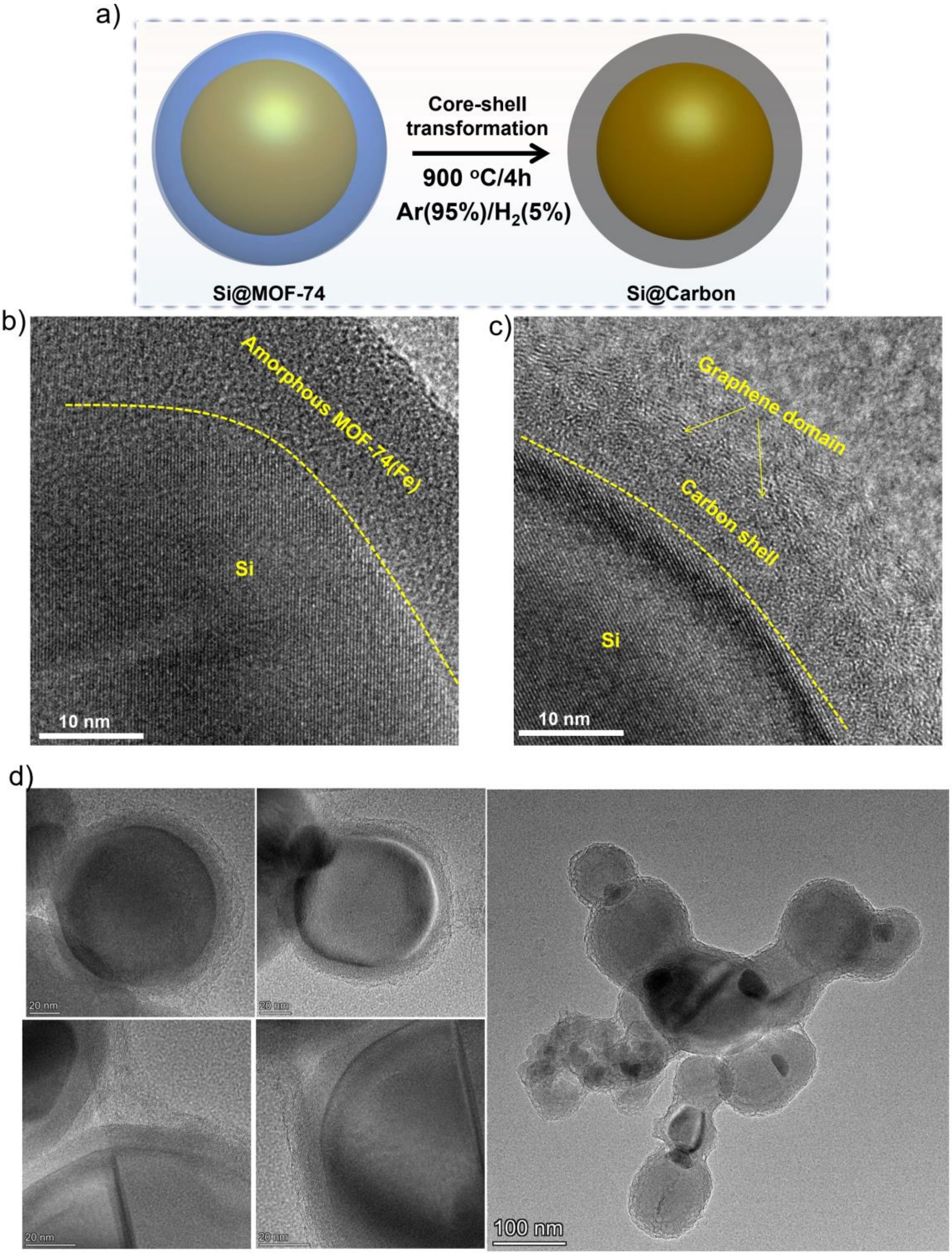

Supplementary Figure 81. (a) Schematic illustration of the transformation of Si@aMOF-74(Fe) into Si@Carbon core-shell colloids. (b) HRTEM images of the Si@aMOF-74(Fe). (c, d) (HR)TEM images of Si@Carbon.

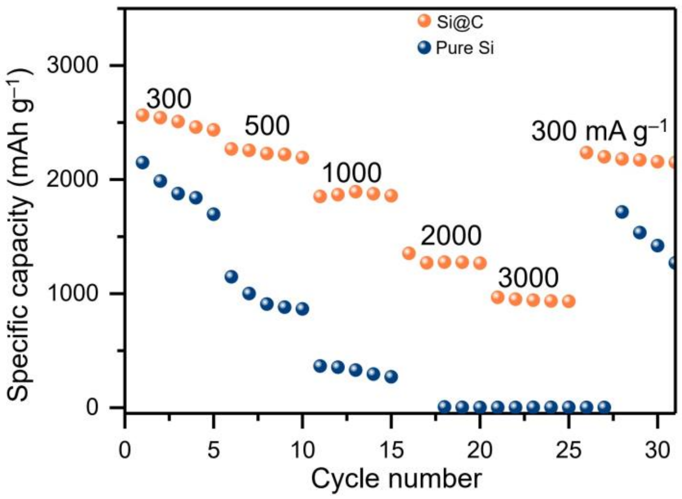

Supplementary Figure 82. Rate performances of Si@Carbon and pure Si. Compared with a pure Si anode, the Si@Carbon exhibits superior rate performance due to the enhanced conductivity of the carbon shell.

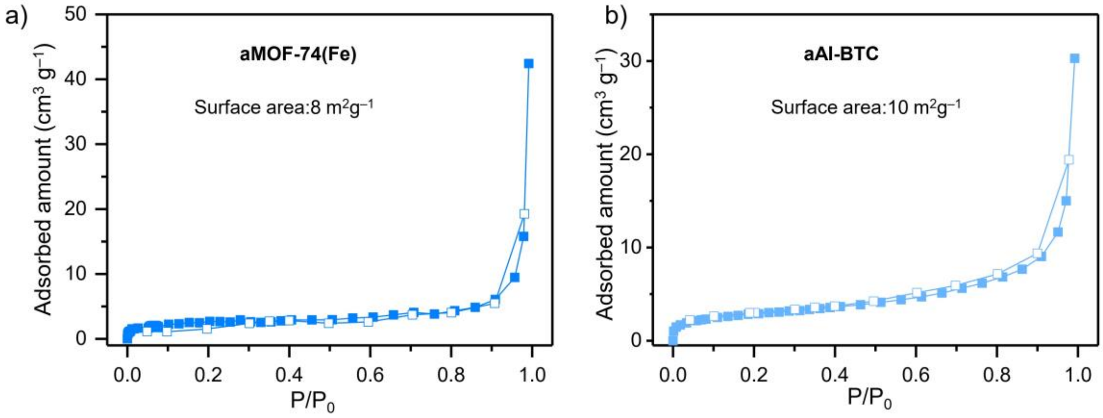

Supplementary Figure 83. The N_2 adsorption-desorption isotherm of a-MOF-74 (Fe) and a-Al-BTC sphere.

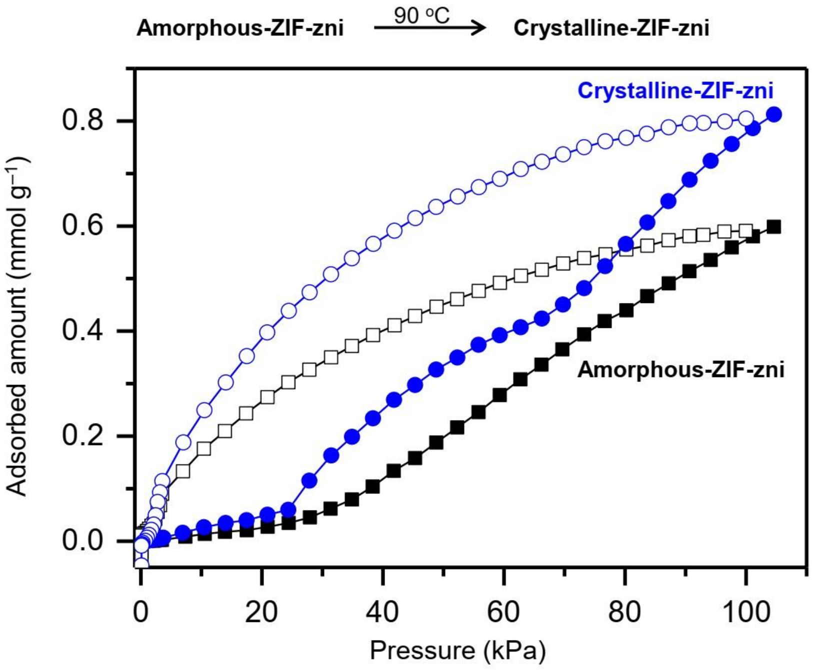

Supplementary Figure 84. The CO_2 adsorption-desorption isotherm collected at 273 K of a-ZIF-zni and its corresponding crystalline product under heat treatment (90 °C).

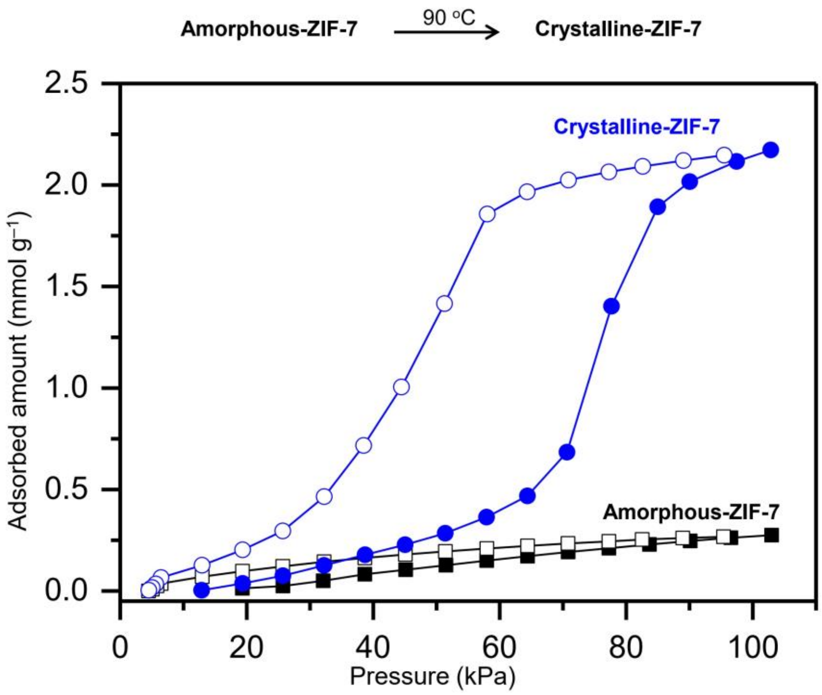

Supplementary Figure 85. The CO_2 adsorption-desorption isotherm collected at 298 K of a-ZIF-7 and its corresponding crystalline product under heat treatment (90 °C). The crystalline ZIF-7 exhibits a typical gate-open behavior in CO_2 adsorption-desorption.

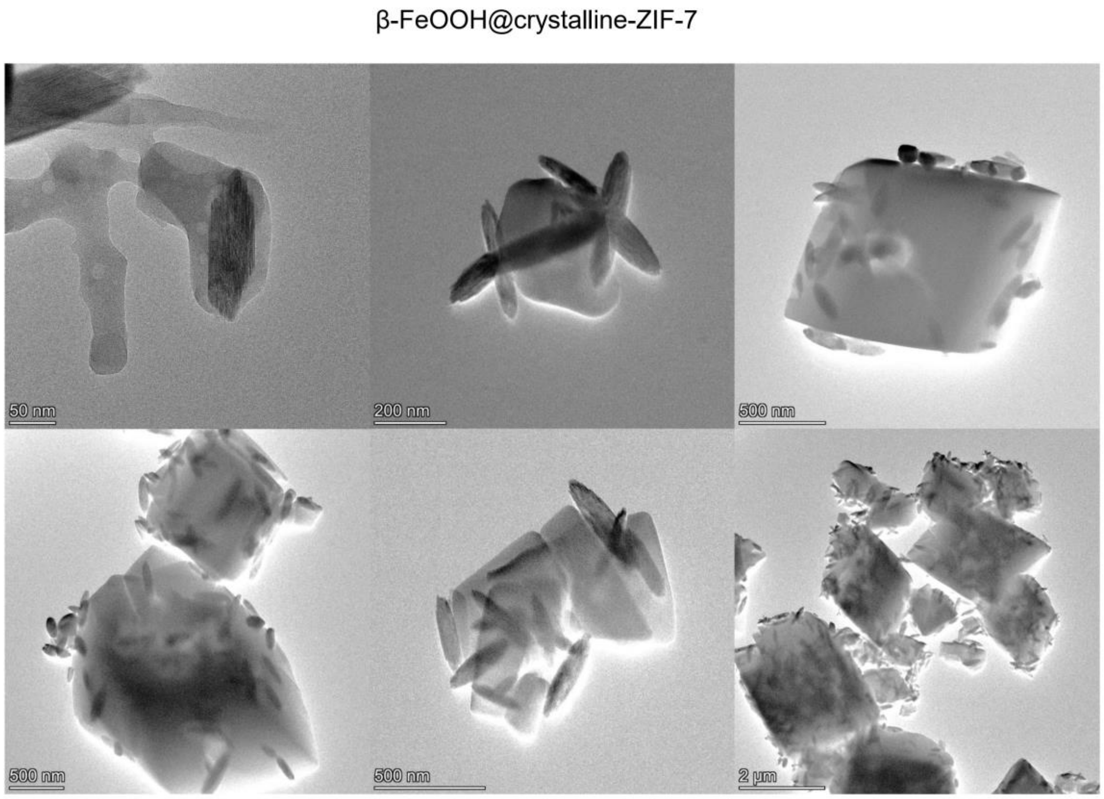

Supplementary Figure 86. The TEM images of corresponding β-FeOOH/crystalline ZIF-7 composites under heat treatment (50 ºC).

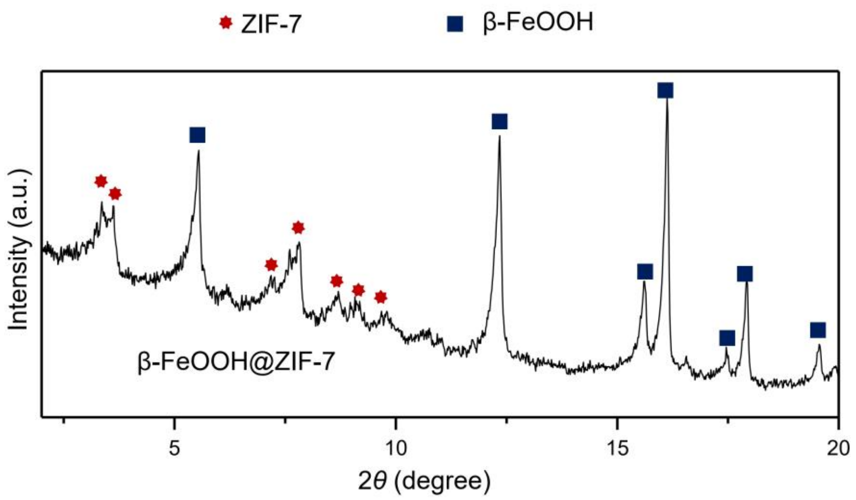

Supplementary Figure 87. The PXRD patterns of corresponding β-FeOOH/crystalline ZIF-7 composites under heat treatment (50 ºC).

	Maximum pore volume (p/p° = 0.016)
Amorphous ZIF-7	0.011 cm^3/g
Crystalline ZIF-7	0.089 cm^3/g
Amorphous ZIF-zni	0.024 cm^3/g
Crystalline ZIF-zni	0.033 cm^3/g

Supplementary Table 1. The maximum pore volume calculated based on CO_2 sorption.

3. Supplementary References

1. Wang, H.; Li, K.; Xu, C.; Xu, S.; Li, G., Large-scale solvothermal synthesis of Ag nanocubes with high SERS activity. *Journal of Alloys and Compounds* **2019,** *772*, 150-156.
2. Sau, T. K.; Rogach, A. L.; Döblinger, M.; Feldmann, J., One-step high-yield aqueous synthesis of size-tunable multispiked gold nanoparticles. *Small* **2011,** *7* (15), 2188-2194.
3. Liu, M.; Zheng, Y.; Zhang, L.; Guo, L.; Xia, Y., Transformation of Pd nanocubes into octahedra with controlled sizes by maneuvering the rates of etching and regrowth. *Journal of the American Chemical Society* **2013,** *135* (32), 11752-11755.
4. Hu, H.; Guan, B. Y.; Lou, X. W. D., Construction of complex CoS hollow structures with enhanced electrochemical properties for hybrid supercapacitors. *Chem* **2016,** *1* (1), 102-113.
5. Hu, M.; Furukawa, S.; Ohtani, R.; Sukegawa, H.; Nemoto, Y.; Reboul, J.; Kitagawa, S.; Yamauchi, Y., Synthesis of Prussian blue nanoparticles with a hollow interior by controlled chemical etching. *Angewandte Chemie International Edition* **2012,** *51* (4), 984-988.
6. Le, Z.; Li, W.; Dang, Q.; Jing, C.; Zhang, W.; Chu, J.; Tang, L.; Hu, M., A high-power seawater battery working in a wide temperature range enabled by an ultra-stable Prussian blue analogue cathode. *Journal of Materials Chemistry A* **2021,** *9* (13), 8685-8691.
7. Li, W.; Yang, J.; Wu, Z.; Wang, J.; Li, B.; Feng, S.; Deng, Y.; Zhang, F.; Zhao, D., A versatile kinetics-controlled coating method to construct uniform porous TiO_2 shells for multifunctional core–shell structures. *J. Am. Chem. Soc.* **2012**, 134, 11864-11867.
8. Mao, Y.; Jiang, W.; Xuan, S.; Fang, Q.; Leung, K. C.-F.; Ong, B. S.; Wang, S.; Gong, X., Rod-like β-FeOOH@ poly (dopamine)–Au–poly (dopamine) nanocatalysts with improved recyclable activities. *Dalton Transactions* **2015,** *44* (20), 9538-9544.
9. Henzie, J.; Etacheri, V.; Jahan, M.; Rong, H.; Hong, C. N.; Pol, V. G., Biomineralization-inspired crystallization of monodisperse α-Mn 2 O 3 octahedra and assembly of high-capacity lithium-ion battery anodes. *Journal of Materials Chemistry A* **2017,** *5* (13), 6079-6089.
10. Han, X.; Jin, M.; Xie, S.; Kuang, Q.; Jiang, Z.; Jiang, Y.; Xie, Z.; Zheng, L., Synthesis of tin dioxide octahedral nanoparticles with exposed high-energy {221} facets and enhanced gas-sensing properties. *Angewandte Chemie International Edition* **2009,** *48* (48), 9180-9183.
11. Armstrong, A. R.; Armstrong, G.; Canales, J.; Bruce, P. G., TiO2-B nanowires. *Angewandte Chemie International Edition* **2004,** *43* (17), 2286-2288.
12. Bai, Y.; Yang, T.; Gu, Q.; Cheng, G.; Zheng, R., Shape control mechanism of cuprous oxide nanoparticles in aqueous colloidal solutions. *powder technology* **2012,** *227*, 35-42.
13. Deng, H.; Li, X.; Peng, Q.; Wang, X.; Chen, J.; Li, Y., Monodisperse magnetic single-crystal ferrite microspheres. *Angewandte Chemie International Edition* **2005,** *44* (18), 2782-2785.
14. Weng, S.; Chen, B.; Xie, L.; Zheng, Z.; Liu, P., Facile in situ synthesis of a Bi/BiOCl nanocomposite with high photocatalytic activity. *Journal of Materials Chemistry A* **2013,** *1* (9), 3068-3075.
15. Hui, J.; Xiang, G.; Xu, X.; Zhuang, J.; Wang, X., Monodisperse F-substituted hydroxyapatite single-crystal nanotubes with amphiphilic surface properties. *Inorganic chemistry* **2009,** *48* (13), 5614-5616.
16. Zai, J.; Zhu, J.; Qi, R.; Qian, X., Nearly monodispersed In (OH) 3 hierarchical nanospheres and nanocubes: tunable ligand-assisted synthesis and their conversion into hierarchical In 2 O 3 for gas sensing. *Journal of Materials Chemistry A* **2013,** *1* (3), 735-745.
17. Wang, F.; Han, Y.; Lim, C. S.; Lu, Y.; Wang, J.; Xu, J.; Chen, H.; Zhang, C.; Hong, M.; Liu, X., Simultaneous phase and size control of upconversion nanocrystals through lanthanide doping. *nature* **2010,** *463* (7284), 1061-

1065.

18. Zhao, Z.; Zhang, K.; Zhang, J.; Yang, K.; He, C.; Dong, F.; Yang, B., Synthesis of size and shape controlled PbS nanocrystals and their self-assembly. *Colloids and Surfaces A: Physicochemical and Engineering Aspects* **2010,** *355* (1-3), 114-120.
19. Wang, H.; Lang, X.; Hao, R.; Guo, L.; Li, J.; Wang, L.; Han, X., Facet-defined AgCl nanocrystals with surface-electronic-structure-dominated photoreactivities. *Nano Energy* **2016,** *19*, 8-16.
20. Bao, X.; Zhao, J.; Sun, J.; Hu, M.; Yang, X., Polydopamine nanoparticles as efficient scavengers for reactive oxygen species in periodontal disease. *ACS nano* **2018,** *12* (9), 8882-8892.
21. Geng, P.; Wang, L.; Du, M.; Bai, Y.; Li, W.; Liu, Y.; Chen, S.; Braunstein, P.; Xu, Q.; Pang, H., MIL-96-Al for Li–S batteries: shape or size? *Advanced Materials* **2022,** *34* (4), 2107836.
22. Teng, Z.; Su, X.; Zheng, Y.; Sun, J.; Chen, G.; Tian, C.; Wang, J.; Li, H.; Zhao, Y.; Lu, G., Mesoporous silica hollow spheres with ordered radial mesochannels by a spontaneous self-transformation approach. *Chemistry of Materials* **2013,** *25* (1), 98-105.
23. Wee, L. H.; Lohe, M. R.; Janssens, N.; Kaskel, S.; Martens, J. A., Fine tuning of the metal–organic framework $Cu_3(BTC)_2$ HKUST-1 crystal size in the 100 nm to 5 micron range. *Journal of Materials Chemistry* **2012**, 22 (27), 13742.
24. Dippel, A.-C.; Liermann, H.-P.; Delitz, J. T.; Walter, P.; Schulte-Schrepping, H.; Seeck, O. H.; Franz, H., Beamline P02. 1 at PETRA III for high-resolution and high-energy powder diffraction. *Journal of synchrotron radiation* **2015,** *22* (3), 675-687.
25. Kieffer, J.; Valls, V.; Blanc, N.; Hennig, C., New tools for calibrating diffraction setups. *Journal of synchrotron radiation* **2020,** *27* (2), 558-566.
26. Juhás, P.; Davis, T.; Farrow, C. L.; Billinge, S. J., PDFgetX3: a rapid and highly automatable program for processing powder diffraction data into total scattering pair distribution functions. *Journal of applied crystallography* **2013,** *46* (2), 560-566.
